# Microwave-Assisted Synthesis of Aminophosphonic Derivatives and Their Antifungal Evaluation against *Lomentospora prolificans*

**DOI:** 10.3390/molecules28103995

**Published:** 2023-05-10

**Authors:** Zuleyma Martínez-Campos, Mariana Elizondo-Zertuche, Emanuel Hernández-Núñez, Eugenio Hernández-Fernández, Efrén Robledo-Leal, Susana T. López-Cortina

**Affiliations:** 1 Laboratorio de Química Industrial, Facultad de Ciencias Químicas, Universidad Autónoma de Nuevo León, Av. Universidad s/n Cd. Universitaria, San Nicolás de los Garza 66455, Nuevo León, Mexico; zuleyma.martinezc@uanl.edu.mx; 2 Departamento de Microbiología, Facultad de Medicina, Universidad Autónoma de Nuevo León, Madero y Dr. Aguirre Pequeño, Col. Mitras Centro, Monterrey 64460, Nuevo León, Mexico; melizondoz@uanl.edu.mx; 3 Departamento de Recursos del Mar, Centro de Investigación y de Estudios Avanzados del Instituto Politécnico Nacional, Unidad Mérida, Mérida 97310, Yucatán, Mexico; emanuel.hernandez@cinvestav.mx; 4 Laboratorio de Micología y Fitopatología, Facultad de Ciencias Biológicas, Universidad Autónoma de Nuevo León, Av. Universidad s/n Cd. Universitaria, San Nicolás de los Garza 66455, Nuevo León, Mexico

**Keywords:** antifungal activity, α-aminophosphonic derivatives, *L. prolificans*

## Abstract

*Lomentospora prolificans* is a pathogenic and multidrug-resistant fungus that can infect both immunocompetent and immunocompromised patients, with mortality rates up to 87%. The World Health Organization (WHO) included this fungal species in its first list of 19 priority fungal pathogens, which focused on fungal pathogens that can cause invasive acute and subacute systemic fungal infections. Therefore, there is a growing interest in finding new therapeutic alternatives. In this work, the synthesis of twelve α-aminophosphonates by the microwave-assisted Kabachnik–Fields reaction and twelve α-aminophosphonic acids by a monohydrolysis reaction is reported. All compounds were evaluated by the agar diffusion method as a preliminary screening in comparison with voriconazole, showing inhibition halos for compounds **7**, **11**, **13**, **22** and **27**. The five active compounds in the preliminary tests were evaluated against five strains of *L. prolificans* following protocol M38-A2 from CLSI. The results showed that these compounds exhibit antifungal activity in the concentration range of 900->900 μg/mL. Cytotoxicity against healthy COS-7 cells was also evaluated by the MTT assay, and it was shown that compound **22** was the least cytotoxic, with a viability of 67.91%, comparable to the viability exhibited by voriconazole (68.55%). Docking studies showed that the possible mechanism of action of the active compounds could be through the inhibition of the enzyme lanosterol-14-alpha-demethylase in an allosteric hydrophobic cavity.

## 1. Introduction

Fungal infections affect more than one billion people each year, of which 150 million develop severe symptoms and approximately 1.6 million die [1]. *Lomentospora prolificans* is a highly virulent filamentous fungus that can cause a wide spectrum of clinical manifestations [2]. *L. prolificans* can infect both immunocompetent and immunocompromised patients, the immunocompromised patients being the most susceptible, causing disseminated infections with mortality rates up to 87% [3,4]. In fact, the World Health Organization (WHO) included this fungal species in its first list of 19 priority fungal pathogens, selected due to their perceived public health importance [5].

Currently, there are three main classes of clinically approved antifungal compounds: azoles, which inhibit ergosterol synthesis (fluconazole, voriconazole, isavuconazole, itraconazole), polyenes, which directly target ergosterol in the fungal membrane (some formulations of amphotericin B), and echinocandins, which block *β*-glucan synthesis in the fungal cell wall [6]. However, *L. prolificans* is highly resistant to most available antifungal agents, making infections caused by this fungus difficult to treat [3,7]. In addition, most of the available drugs have high toxicity levels and several side effects, which significantly limit their use [6]. Although great advances have been made in the study of *L. prolificans*, there is an urgent need to develop new therapeutic alternatives to treat these infections with less toxic and more selective drugs.

α-aminophosphonates and α-aminophosphonic acids are organic compounds containing one or more C-P(O)(OR)_2_ or C-P(O)(OH)_2_ (R = alkyl or aryl) groups and are considered structural analogs of natural α-amino acids, which exhibit a broad pharmacological spectrum, including potent antibacterial, antiviral, and antifungal activity, among others [8,9,10]. Given these different biological applications, it becomes important to synthesize novel aminophosphonic derivatives. One of the most widely used methodologies for obtaining α-aminophosphonates is the Kabachnik–Fields (KF) reaction, a one-pot tricomponent reaction between a carbonyl compound (aldehyde or ketone), an amine and dialkyl or diaryl phosphite [11,12]. A wide range of catalysts has been employed for this reaction, from simple catalysts to structurally complex ones, some of which are not only expensive but also not environmentally friendly, such as scandium tris(dodecyl sulfate) [13], metal triflates [14,15], InCl_3_ [16], Ln(OTf)_3_ [17], SmI_2_ [18] and AlCl_3_ [19], among others [20,21,22,23,24]. However, over the years, several authors have reported that the KF reaction proceeds with good yields using biodegradable catalysts [25,26] or even catalyst-free and solvent-free conditions, offering an environmentally friendly reaction. In fact, the microwave (MW) promoted reaction catalyst-free and solvent-free has proven to be a remarkable alternative, given that, compared to conventional heating, it has multiple advantages such as shorter reaction times, high efficiency, cost-effectiveness and environmental friendliness [24,27,28,29,30,31,32,33,34].

Considering this, we report the synthesis of new α-aminophosphonic derivatives by the microwave-assisted Kabachnik–Fields reaction and their antifungal evaluation against five strains of the multidrug-resistant fungus *Lomentospora prolificans*.

In a previous work reported by our research group [35], a series of α-aminophosphonates and monohydrolyzed α-aminophosphonic acids was evaluated against different strains of the multi-resistant fungi *Scedosporium* spp. Compound **1** was the most active compound of the series, showing activity in the range of 648.76–700 μg/mL, in comparison to voriconazole, which presented an activity range of 0.44–2.64 μg/mL; it was less active but exhibited lower cytotoxicity. Prior to this work, there have been no reports of aminophosphonic derivatives with antifungal activity against multidrug-resistant *Scedosporium* fungi; thus, we consider the molecules reported in this work as the starting point for the design and synthesis of new α-aminophosphonic derivatives with antifungal potential. It is important to notice that *Lomentospora* is generally more resistant to antifungal drugs than *Scedosporium* [36]. 

**Figure 1:**
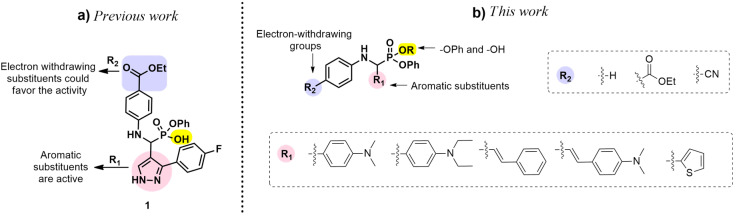
(**a**) Structure–activity relationships for compound **1**, (**b**) Structure proposal for new α-aminophosphonate derivatives.

Analyzing the scaffold of compound **1**, some structural modifications were made in an effort to uncover new structural features that could favor antifungal activity and be a starting point for new antifungal drug discovery. Analyzing the structural features of **1**, we hypothesized that maintaining aromatic substituents at R_1_ and electron-withdrawing groups at R_2_ could favor the activity (Figure 1a); therefore, in this work, we proposed the inclusion of different aromatic substituents at R_1_, keeping the ester group at R_2_, having a nitrile group or leaving it without a substituent (Figure 1b).

## 2. Results and Discussion

### 2.1. Chemistry

**Scheme 1:**
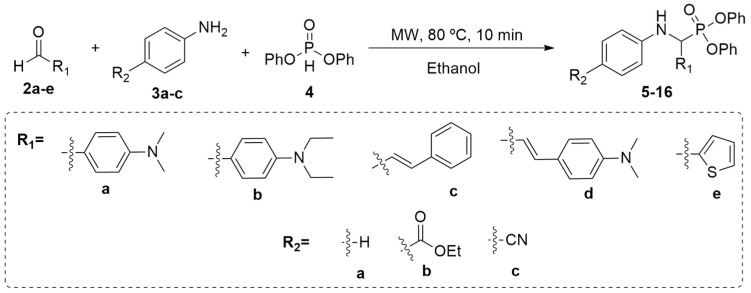
Synthesis of α-aminophosphonates **5**–**16** by MW-assisted KF reaction.

α-aminophosphonates **5**–**16** were synthetized by the microwave-assisted Kabachnik–Fields (KF) reaction without a catalyst (Scheme 1), in which the corresponding aldehydes **2a**–**e**, an amine **3a**–**c** and diphenyl phosphite **4** in ethanol were reacted at 80 °C for 10 min. The progress of the reaction was monitored by TLC.

**Scheme 2:**
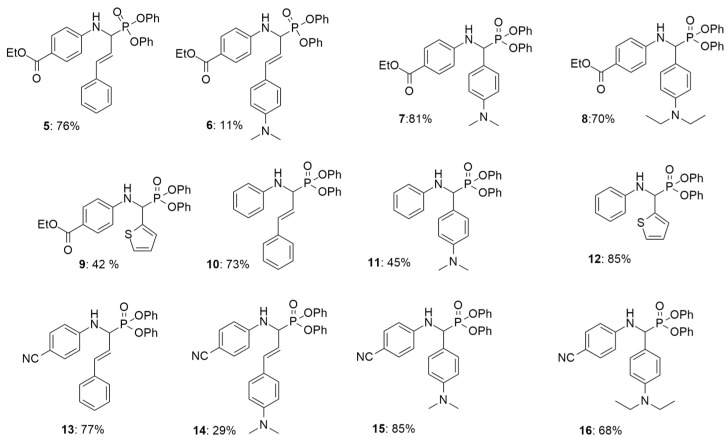
Yields of α-aminophosphonates **5**–**16**.

Once the reaction was completed, the reaction mixture reached room temperature and the product precipitated (Scheme 2) due to their low solubility in ethanol. Compounds **5**–**16** were isolated by vacuum filtration and washed with cold diethyl ether.

**Scheme 3:**
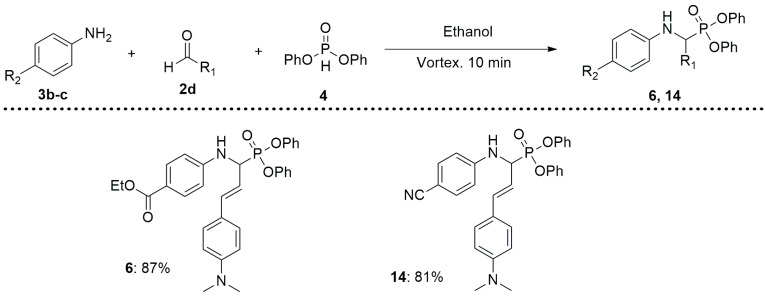
Synthesis of α-aminophosphonates **6** and **14** by vortex mixing.

Products **5**, **7**–**13** and **15**–**16** were isolated by filtration without any further purification, with good yields (42–85%). However, compounds **6** and **14** were obtained in low yields when the proposed reaction conditions were followed; the reason for this may be because 4-dimethylaminocinnamaldehyde **2d** is a very unstable compound [37], which favored the formation of undesirable by-products. For this reason, it was necessary to synthesize the aminophosphonates **6** and **14** through another methodology. Therefore, the KF reaction was carried out between 4-dimethylaminocinnamaldehyde **2d**, diphenyl phosphite **4** and the corresponding amines **3b**,**c** using vortex mixing (Scheme 3) [35]. Remarkably, there was an increase in the yields of compounds **6** and **14** from 11% and 29%, to 87% and 81%, respectively. All the spectra of synthesized aminophosphonates are supplied in the Supplementary Materials.

It is important to note that, in this work, it was not possible to carry out the KF reaction without a solvent due to the heterogeneity of the components. Some authors have suggested using water as a green alternative; however, it is common that the organic components are not water-soluble, which may limit its use [25,38,39,40]. Furthermore, Mu et al. reported a study in which ethanol was used as a solvent under conventional heating conditions; however, the reaction did not proceed, and without the solvent, the reaction time was 2 h [24]. Furthermore, Keglevich et al. studied the microwave-assisted KF reaction with different solvents and concluded that, among the investigated solvents, ethanol proved to be the most efficient, with good yields, but in comparison with the results observed in this work, the reaction time was 25 min, and it was necessary to use an excess of the phosphorous compound [33], while in this work, the reaction time under microwave irradiation was only 10 min, it was not necessary to use a large excess of the phosphorate compound and, as mentioned above, the aminophosphonates had the advantage of crystallizing in ethanol; therefore, it was not necessary to use column chromatography to purify them.

**Scheme 4:**
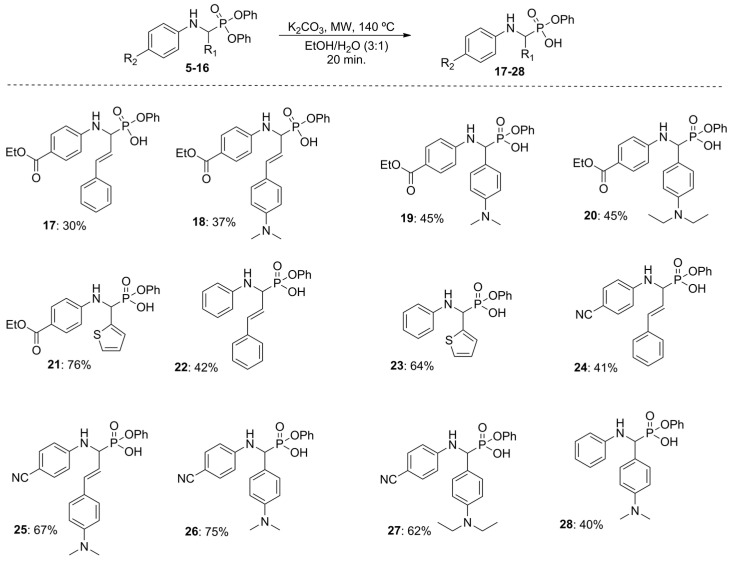
Synthesis of α-aminophosphonic acids **17**–**28**.

The next step in the synthesis involved basic monohydrolysis of the α-aminophosphonates **5**–**16** using potassium carbonate by microwave irradiation, in an ethanol/water mixture (3:1) as the reaction medium (Scheme 4). The reaction mixture was heated at 140 °C for 20 min. The progress of the reaction was monitored by TLC, and the solvents were evaporated under reduced pressure. The reaction crude product was purified by column chromatography in a gradient system, starting with ethyl acetate to remove the less polar impurities and ending with methanol/ethyl acetate (1:1) to obtain the monoacids as pure solids.

It is noteworthy that the hydrolysis reaction proceeded by selectively hydrolyzing one of the -O-phenyl substituents on the phosphonate group, which was confirmed by ^1^H NMR and ^13^C NMR, where the observed signals agreed with the expected ones for compounds **17**–**28** (Table 1). Potassium carbonate and water reacted to form a catalytic number of hydroxide ions, which are the species responsible for promoting the specific monohydrolysis reaction [35]. All the spectra of synthesized aminophosphonic acids are supplied in the Supplementary Materials.

Monoacids **18** and **25** were not obtained at 140 °C for 20 min because the α-aminophosphonates **6** and **14** are unstable at high temperatures. Therefore, to obtain these monoacids, the microwave reaction time was decreased from 20 min to 10 min, favoring the formation of **18** and **25** with yields of 37% and 67%, respectively.

The structures of the compounds **5**–**28** were confirmed by ^1^H and ^13^C NMR, as well as high-resolution mass spectroscopy. It is important to point out some specific coupling constants (*J*_H-P_ and *J*_C-P_), which were some of the key points in corroborating the union of the three components of the KF reaction (Table 1). In ^1^H NMR, the characteristic hydrogen-phosphorus coupling constants were observed for both the α-aminophosphonates **5**–**16** and the corresponding monoacids **17**–**28**, ranging between 20 and 26 Hz. On the other hand, characteristic C-P coupling constants corresponding to the C-P bond (between 138 and 164 Hz) were observed in ^13^C NMR for each of the α-aminophosphonates and α-aminophosphonic acids.

### 2.2. In Vitro Assays

Compounds **5**–**28** underwent biological testing to determine whether any of the compounds could inhibit the growth of different multidrug-resistant strains of *L. prolificans*.

The in vitro susceptibility of the 24 synthesized compounds described above was preliminarily evaluated by the agar diffusion method against *Lomentospora* strains 11-2242, 10-1167 and 09-1125; this method is based on the visual evaluation of the diameter of the growth inhibition zones. For this purpose, agar plates were inoculated with an inoculum of strains (11-2242), (10-1167) and (09-1125). Then, filter paper discs (5 mm in diameter) containing the test compounds at a concentration of 1 mg/mL were placed on the agar surface. Subsequently, the Petri dishes were incubated for 72 h. For this experiment, voriconazole (VRC) was used as a positive control, and it was observed that α-aminophosphonates **5**, **6**, **8**–**10**, **12**, **14** and **15** did not inhibit the growth of any of the *L. prolificans* strains tested, and only compounds **7**, **11** and **13** exhibited inhibition halos. On the other hand, within the series of α-aminophosphonic acids, compounds **22** and **27** also showed inhibition halos.

### 2.3. Antifungal Activity Evaluation (M38-A2 Protocol)

Compounds that exhibited inhibition halos in the preliminary assay (**7**, **11**, **13**, **22** and **27**) were evaluated against five strains of *L. prolificans* (11-2242, 05-21909, 10-1167, 13-196 and 09-1125) at different concentrations using the M38-A2 protocol, which challenges different concentrations of the drugs against a standard inoculum, visually registering changes in the fungal growth at 48 and 72 h of incubation. The drug concentration range was 600 μg/mL to 1000 μg/mL. The MIC_100_ are shown in Table 2, and these were determined for compounds **7**, **11**, **13**, **22** and **27** and antifungal VRC. It was observed that the compounds inhibited the growth of the five strains tested at concentrations of 900 μg/mL, while voriconazole inhibited growth at concentrations of 16 μg/mL.

### 2.4. Molecular Docking

Voriconazole acts by inhibiting lanosterol-14-alpha-demethylase (PDB ID: 5HS1), a protein involved in ergosterol biosynthesis, the main target of azole antifungal drugs [41]. Based on this, and given that VRC has been used as a control in the present study, it is proposed that active compounds **7**, **11**, **13**, **22** and **27** could exert their antifungal action through a similar mechanism of action; therefore, molecular docking studies were carried out to support this hypothesis.

The methodology for the validation of the active site of the lanosterol-14α-demethylase protein (PDB ID: 5HS1) was performed and described in a previous work by Cordero-Díaz et al. [35] where validation was performed with the native co-crystallized ligand Voriconazole, with an RMSD of less than 2.5 Å. The Autodock-Vina program (https://vina.scripps.edu/; accessed on 15 January 2023) was used to predict molecular docking [42,43], using 1000 modes, 1000 exhaustiveness and 10 replicates for each one, selecting the lowest energy value [35,44]. All figures were visualized with Pymol (Schrödinger, San Diego, CA, USA; http://www.pymol.org/; accessed on 15 December 2022) and Discovery Studio Visualizer 4.5 software.

**Figure 2:**
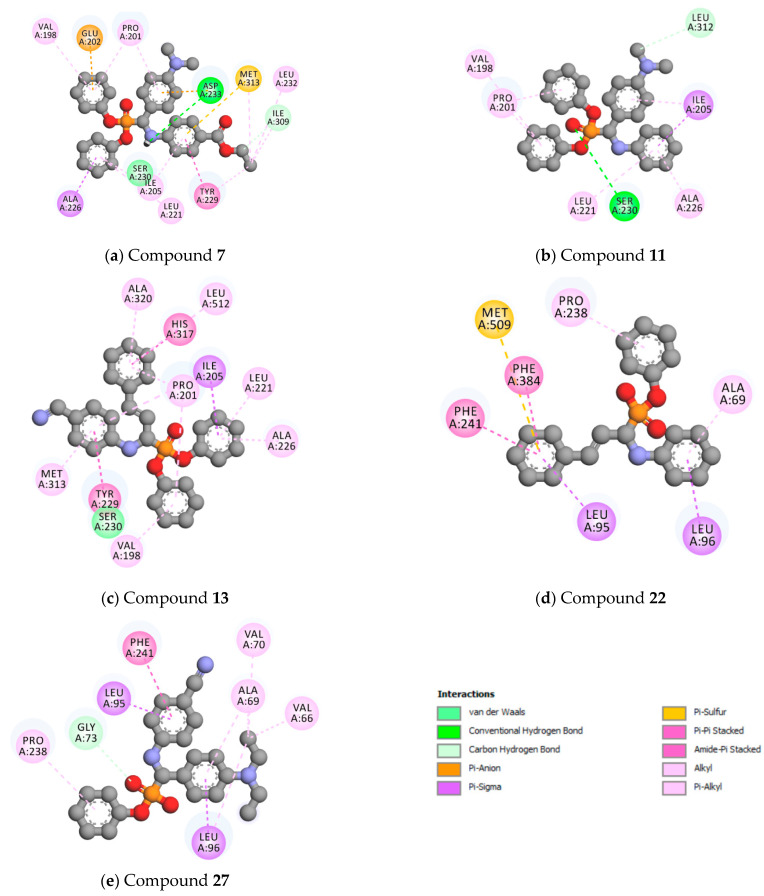
Docking interactions between active compounds and lanosterol-14-alpha-demethylase.

Molecular docking studies showed that compounds **7**, **11**, **13**, **22** and **27** interact with the lanosterol-14-alpha-demethylase protein in an allosteric hydrophobic cavity. In analyzing the docking results, it should be noticed that the active compounds **7**, **11** and **27** share the substituent at R_1_, which consists of an aromatic ring 4-aminoalkyl substituted. Both 4-dimethylamino and 4-diethylamino substituents are electro-donating groups that could concentrate the electron density in the aromatic ring, favoring different types of interactions. In this context, compound **7** displayed π-anion and π-alkyl interactions with Asp233 and Pro201, respectively (Figure 2a). Compound **27** presented π-sigma and π-alkyl interactions with Leu96 and Ala69, respectively (Figure 2e), and a π-alkyl interaction with Ile205 was observed for compound **11** (Figure 2b).

In the case of compounds **13** and **22**, even though the chain length supporting the phenyl group at R_1_ was increased, the in vitro inhibitory activity was preserved. The additional double bond containing a π bond did not favor additional interactions with the therapeutic target in this part of the molecule, but the aromatic ring interacted with a greater number of amino acids in this position. For compound **13**, interactions with Ala320, His317, Pro201 and Leu512 were observed (Figure 2c), while compound **22** showed interactions with Leu95, Phe241, Phe384 and Met509 (Figure 2d).

On the other hand, it was observed that electro-withdrawing groups at the R_2_ position favor a higher number of hydrophobic interactions with the aromatic ring. As shown in Figure 2a, compound **7** interacts with Ile205, Tyr229, Ile309, Leu232 and Met313, compound **13** interacts with Met313, Tyr229, Ser230 and Pro201 (Figure 2c) and compound **27** interacts with Leu96, Ala69, Val70 and Val66 (Figure 2e), and fewer interactions were observed for compounds **11** and **22** (without a substituent in the R_2_ position), which just showed interactions with Ala226 and Ile 205 and Ala69 and Leu96, respectively (Figure 2b,d).

Finally, it was observed that the less sterically hindered aminophosphonic acids **22** and **27** interact with the protein in a different region from the proposed active site, while compounds that are more sterically hindered, such as aminophosphonates **7**, **11** and **13**, remained at the proposed active site. Interestingly, as the size of the substitution in the phosphonate group decreases, the ligand interacts in a region of the protein that is not the proposed active site.

**Scheme 5:**
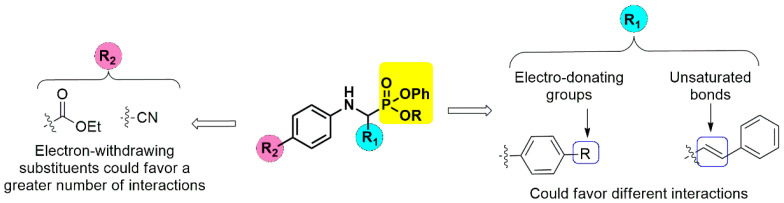
Structure–Activity Relationship.

Based on the previous analysis, it is recommended to consider some structural features in the design of future aminophosphonates and aminophosphonic acid derivatives. In the case of R_1_, it is important to include para-substituted phenyl rings with electro-donating groups. In addition, the R_1_ with higher steric hindrance, at least in the molecular docking, is relevant to remain in the proposed active site. On the other hand, electron-withdrawing substituents at R_2_ could favor a greater number of interactions with the biological target, and it is important to preserve the phosphonate group (Scheme 5).

### 2.5. Cytotoxicity

In order to compare the cytotoxicity at high concentrations of the active compounds **7**, **11**, **13**, **22** and **27** compared to VRC, cytotoxicity studies were performed using the MTT test in COS-7 cells at a concentration of 1000 µM (Table 3). In this study, compound **22** was found to be the least cytotoxic, with a viability of 67.91%, comparable to the viability exhibited by VRC: 68.55%.

## 3. Materials and Methods

### 3.1. Synthesis of α-Aminophosphonates **5**–**16**

In a 10 mL microwave (Anton Paar Monowave 300) vial with a magnetic stirrer, 1 eq. of the corresponding aldehyde **2a**–**e**, 1 eq. of the corresponding amine **3a**–**c**, 1.1 eq. of diphenyl phosphite **4** and 3 mL of absolute ethanol were added. The vial was placed in the microwave cavity, and the mixture reacted at 80 °C for 10 min. The reaction mixture was allowed to cool at room temperature to induce the precipitation of the product, which was isolated by vacuum filtration and washed with cold diethyl ether. This method was used for the synthesis of all the following molecules.

(E)-ethyl 4-((1-(diphenoxyphosphoryl)-3-phenylallyl)amino)benzoate (**5**)

The desired aminophosphonate **5** was obtained from ethyl 4-aminobenzoate **3b** (0.5 g, 3.03 mmol), cinnamaldehyde **2c** (0.4 g, 3.03 mmol) and diphenyl phosphite **4** (0.78 g, 3.3 mmol) as a pale yellow solid (1.18 g, 76%), mp 129–132 °C, ^1^H NMR (700 MHz, CDCl_3_) δ(ppm): 7.9 (d, *J =* 7.9 Hz, 2H, C*H*_arom_), 7.32–7.22 (m, 9H, C*H*_arom_), 7.18 (d, *J =* 7.7 Hz, 2H, C*H*_arom_), 7.16–7.12 (m, 2H, C*H*_arom_), 7.09 (d, *J =* 7.8 Hz, 2H, C*H*_arom_), 6.68 (dd, *J* = 16.1 Hz, 5.1 Hz, 1H, C*H*_vinylic_), 6.69 (d, *J =* 8.0 Hz, 2H, C*H*_arom_), 6.35 (dt, *J =* 15.8 Hz, 5.2 Hz, 1H, C*H*_vinylic_), 4.97 (s, 1H, N*H*), 4.92 (dd, *J =* 25.9 Hz, 6.1 Hz, 1H, C*H*-P), 4.32 (q, *J =* 7.0 Hz, 2H, C*H*_2_), 1.36 (t, *J =* 7.0 Hz, 3H, C*H*_3_). ^13^C NMR (151 MHz, CDCl_3_) δ(ppm): 166.6, 150.2 (d, *J*_C-P_ = 7.1 Hz), 150.1 (d, *J*_C-P_ = 7.1 Hz), 149.9 (d, *J*_C-P_ = 11.4 Hz),149.8 (d, *J*_C-P_ = 11.5 Hz) 135.7 (d, *J*_C-P_ = 3.2 Hz), 134.5, 134.4, 131.5, 129.9, 129.8, 128.6, 128.2, 126.7, 125.5, 121.2 (d, *J*_C-P_ = 3.6 Hz), 120.6 (d, *J*_C-P_ = 4.1 Hz), 120.5 (d, *J*_C-P_ = 4.2 Hz), 112.7, 60.4, 53.3 (d, *J*_C-P_ = 156.3 Hz), 14.4.^31^P NMR (243 MHz, CDCl_3_) δ(ppm): 14.1. HRMS (ESI^+^) *m*/*z*, calcd. for C_30_H_29_NO_5_P [M + H]^+^ 514.1783; found 514.1766.

(E)-ethyl 4-((3-(4-(dimethylamino)phenyl)-1-(diphenoxyphosphoryl)allyl)amino) benzoate (**6**)

The desired aminophosphonate **6** was obtained from ethyl 4-aminobenzoate **3b** (0.5 g, 3.03 mmol), 4-dimethylaminocinnamaldehyde **2d** (0.53 g, 3.03 mmol) and diphenyl phosphite **4** (0.78 g, 3.3 mmol) as a yellow solid (1.46 g, 87%), mp 119–121 °C, ^1^H NMR (600 MHz, CDCl_3_) δ(ppm): 7.89 (d, J = 8.9 Hz, 2H, CH_arom_), 7.31–7.24 (m, 4H, CH_arom_), 7.22 (d, J = 8.5 Hz, 2H, CH_arom_), 7.18–7.12 (m, 4H, CH_arom_), 7.09 (d, J = 8.0 Hz, 2H, CH_arom_), 6.71–6.65 (m, 3H, CH_arom_), 6.63 (d, J = 8.7 Hz, 2H, CH_arom_), 6.08 (dt, J = 15.8 Hz, 5.8 Hz, 1H, CH_vinylic_), 4.91–4.79 (m, 2H, CH-P, NH), 4.32 (q, J = 7.1 Hz, 2H, CH_2_), 2.95 (s, 6H, N(CH_3_)_2_), 1.36 (t, J = 7.1 Hz, 3H, CH_3_). ^13^C NMR (176 MHz, CDCl_3_) δ(ppm): 166.6, 150.3, 150.2 (t, J_C-P_ = 9.5 Hz), 150.0 (d, J_C-P_ = 11.5 Hz), 134.6 (d, J_C-P_ = 13.3 Hz), 131.5, 129.9, 129.8, 129.6, 128.7, 127.8, 125.4 (d, J_C-P_ = 3.2 Hz), 120.62 (d, J_C-P_ = 10.9 Hz), 120.6 (d, J_C-P_ = 2.6 Hz), 120.4, 116.2, 115.3, 112.7, 112.3, 60.3, 53.6 (d, J_C-P_ = 157.1 Hz),40.5, 14.4. ^31^P NMR (243 MHz, CDCl_3_) δ(ppm): 14.7. HRMS (ESI^+^) *m*/*z*, calcd. for C_32_H_34_N_2_O_5_P [M + H]^+^ 557.2205; found 557.2189.

Ethyl 4-(((4-(dimethylamino)phenyl)(diphenoxyphosphoryl)methyl)amino)benzoate (**7**)

The desired aminophosphonate **7** was obtained from ethyl 4-aminobenzoate **3b** (0.32 g, 1.94 mmol), 4-(dimethylamino)benzaldehyde **2a** (0.29 g, 1.94 mmol) and diphenyl phosphite **4** (0.5 g, 2.13 mmol) as a yellow solid (0.83 g, 81%), mp 154–156 °C, ^1^H NMR (600 MHz, CDCl_3_) δ(ppm): 7.82 (d, *J =* 8.6 Hz, 2H, C*H*_arom_), 7.36 (dd, *J* = 8.6 Hz, 2.03 Hz, 2H, C*H*_arom_), 7.28–7.23 (m, 2H, C*H*_arom_), 7.22–7.17 (m, 2H, C*H*_arom_), 7.16–12 (m, 1H, C*H*_arom_), 7.11–7.09 (m, 1H, C*H*_arom_), 7.08–7.056(m, 2H, C*H*_arom_), 6.86 (d, *J =* 8.3 Hz, 2H, C*H*_arom_), 6.67 (d, *J =* 8.5 Hz, 2H, C*H*_arom_), 6.63 (d, *J =* 8.6 Hz, 2H, C*H*_arom_), 5.35–5.26 (m, 1H, NH), 5.1 (dd, *J* = 23.5, 4.9 Hz, 1H, C*H*-P), 4.29 (q, *J* = 7.1 Hz, 2H, C*H*_2_), 2.91 (s, 6H, N(CH_3_)_2_), 1.33 (t, *J* = 7.1 Hz, 3H, C*H*_3_). ^13^C NMR (151 MHz, CDCl_3_) δ(ppm): 166.6, 150.7, 150.4 (d, *J*_C-P_ = 9.9 Hz), 150.3 (d, *J*_C-P_ = 9.9 Hz), 150.0 (d, *J*_C-P_ = 14.1 Hz), 131.4, 129.7, 129.6, 129.0 (d, *J*_C-P_ = 6.1 Hz), 125.4, 125.2, 121.0, 120.7 (d, *J*_C-P_ = 4.0 Hz), 120.5 (d, *J*_C-P_ = 4.2 Hz), 120.2, 112.9, 112.7, 60.3, 54.9 (d, *J*_C-P_ = 157.1 Hz), 40.4, 14.4. ^31^P NMR (243 MHz, CDCl_3_) δ(ppm): 14. 9. HRMS (ESI^+^) *m*/*z*, calcd. for C_30_H_32_N_2_O_5_P [M + H]^+^ 531.2049; found 531.2076.

Ethyl 4-(((4-(diethylamino)phenyl)(diphenoxyphosphoryl)methyl)amino)benzoate (**8**)

The desired aminophosphonate **8** was obtained from ethyl 4-aminobenzoate **3b** (0.32 g, 1.94 mmol), 4-(diethylamino)benzaldehyde **2b** (0.34 g, 1.94 mmol) and diphenyl phosphite **4** (0.5 g, 2.13 mmol) as a white solid (0.75 g, 70%), mp 139–141 °C, ^1^H NMR (600 MHz, CDCl_3_) δ(ppm): 7.83 (d, *J* = 8.4 Hz, 2H, C*H*_arom_), 7.31 (d, *J* = 6.7 Hz, 2H, C*H*_arom_), 7.28–7.23 (m, 2H, C*H*_arom_), 7.20–7.16 (m, 2H, C*H*_arom_), 7.16–7.12 (m, 1H, C*H*_arom_), 7.1–7.05 (m, 3H, C*H*_arom_), 6.82 (d, *J* = 8.0 Hz, 2H, C*H*_arom_), 6.65 (d, *J* = 8.4 Hz, 2H, C*H*_arom_), 6.59 (d, *J* = 8.4 Hz, 2H, C*H*_arom_), 5.34 (t, *J* = 8.4 Hz, 1H, N*H*), 5.08 (dd, *J* = 23.3 Hz, 8.5 Hz, 1H, C*H*-P), 4.29 (q, *J* = 6.9 Hz, 2H, C*H*_2_CH_3_), 3.31 (q, *J* = 6.9 Hz, 4H, N(C*H*_2_CH_3_)_2_), 1.33 (t, *J* = 7.1 Hz, 3H, CH_2_C*H*_3_), 1.12 (t, *J* = 7.0 Hz, 6H, N(C*H*_2_CH_3_)_2_). ^13^C NMR (151 MHz, CDCl_3_) δ(ppm): 166.8, 150.5 (d, *J*_C-P_ = 6.7 Hz), 150.4 (d, *J*_C-P_ = 6.2 Hz), 150.18 (d, *J*_C-P_ = 14.0 Hz), 148.01 (d, *J*_C-P_ = 2.0 Hz), 131.4, 129.8, 129.6, 129.3 (d, *J*_C-P_ = 6.2 Hz), 125.5, 125.2, 120.8 (d, *J*_C-P_ = 4.0 Hz), 120.5 (d, *J*_C-P_ = 4.15 Hz), 120.19, 119.5, 112.9, 112.0, 60.4, 54.9 (d, *J*_C-P_ = 157.5 Hz), 44.4, 14.5, 12.6. ^31^P NMR (243 MHz, CDCl_3_) δ(ppm): 15.32. HRMS (ESI^+^) *m*/*z*, calcd. for C_32_H_36_N_2_O_5_P [M + H]^+^ 559.2362; found 559.2331.

Ethyl 4-(((diphenoxyphosphoryl)(thiophen-2-yl)methyl)amino)benzoate (**9**)

The desired aminophosphonate **9** was obtained from ethyl 4-aminobenzoate **3b** (0.32 g, 1.94 mmol), 2-thiopenecarboxaldehyde **2e** (0.22 g, 1.94 mmol) and diphenyl phosphite **4** (0.5 g, 2.13 mmol) as a white solid (0.40 g, 42%), mp 99–102 °C, ^1^H NMR (600 MHz, CDCl_3_) δ(ppm): 7.87 (d, *J* = 8.5 Hz, 2H, C*H*_arom_), 7.29–7.22 (m, 6H, C*H*_arom_), 7.18–7.12 (m, 2H, C*H*_arom_), 7.07 (d, *J* = 7.7 Hz, 2H, C*H*_arom_), 6.99–6.96 (m, 1H, C*H*_arom_), 6.95 (d, *J* = 7.9 Hz, 2H, C*H*_arom_), 6.68 (d, *J* = 8.7 Hz, 2H, C*H*_arom_), 5.49 (dd, *J* = 24.0, 8.3 Hz, 1H, C*H*-P), 5.34–5.25 (m, 1H, N*H*), 4.31 (q, *J* = 7.1 Hz, 2H, C*H*_2_CH_3_), 1.35 (t, *J* = 7.1 Hz, 3H, CH_2_C*H*_3_).^13^C NMR (151 MHz, CDCl_3_) δ(ppm): 166.6, 150.3 (d, *J*_C-P_ = 9.3 Hz), 150.2 (d, *J*_C-P_ = 9.3 Hz), 149.5 (d, *J*_C-P_ = 12.6 Hz), 137.5, 131.6, 129.9 (d, *J*_C-P_ = 7.8 Hz), 127.6 (d, *J*_C-P_ = 2.8 Hz), 127.5, 127.4, 126.3 (d, *J*_C-P_ = 3.8 Hz), 125.7, 125.6, 121.1, 120.7 (d, *J*_C-P_ = 4.0 Hz), 120.4 (d, *J*_C-P_ = 4.4 Hz), 113.0, 60.5, 51.3 (d, *J*_C-P_ = 162.6 Hz), 14.5.^31^P NMR (243 MHz, CDCl_3_) δ(ppm): 12.5. HRMS (ESI^+^) *m*/*z*, calcd. for C_26_H_25_NO_5_PS [M + H]^+^ 494.1191; found 494.1171.

(E)-diphenyl (3-phenyl-1-(phenylamino)allyl)phosphonate (**10**)

The desired aminophosphonate **10** was obtained from aniline **3a** (0.18 g, 1.94 mmol), cinnamaldehyde **2c** (0.25 g, 1.94 mmol) and diphenyl phosphite **4** (0.5 g, 2.13 mmol) as a pale yellow solid (0.62 g, 73%), mp 119–122 °C, ^1^H NMR (700 MHz, CDCl_3_) δ(ppm): 7.34–7.3 (m, 2H, C*H*_arom_), 7.3–7.21 (m, 17H, C*H*_arom_), 7.21–7.09 (m, 8H, C*H*_arom_),6.84–6.76 (m, 2H, C*H*_arom_), 6.74–6.64 (m, 2H, C*H*_arom_, C*H*_vinylic_), 6.36 (d, *J =* 16.4 Hz, 1H, C*H*_vinylic_), 4.85 (dd, *J* = 26.1 Hz, 6.1 Hz, 1H, C*H*-P).^13^C NMR (176 MHz, CDCl_3_) δ(ppm): 150.4 (d, *J*_C-P_ = 9.9 Hz), 150.3 (d, *J*_C-P_ = 9.7 Hz), 146.1 (d, *J*_C-P_ = 12.4 Hz), 136.0 (d, *J*_C-P_ = 3.1 Hz), 134.2, 134.1, 129.8, 129.7, 129.4, 128.5, 128.1, 126.7, 125.4, 122.3 (d, *J*_C-P_ = 3.3 Hz), 120.7 (d, *J*_C-P_ = 4.0 Hz), 120.6 (d, *J*_C-P_ = 4.2 Hz), 119.0, 113.9, 54.1 (d, *J*_C-P_ = 155.7 Hz).^31^P NMR (243 MHz, CDCl_3_) δ(ppm): 14.8. HRMS (ESI^+^) *m*/*z*, calcd. for C_27_H_25_NO_3_P [M + H]^+^ 442.1572; found 442.1515.

Diphenyl ((4-(dimethylamino)phenyl)(phenylamino)methyl)phosphonate (**11**)

The desired aminophosphonate **11** was obtained from aniline **3a** (0.18 g, 1.94 mmol), 4-(dimethylamino)benzaldehyde **2a** (0.29 g, 1.94 mmol) and diphenyl phosphite **4** (0.5 g, 2.13 mmol) as a yellow solid (0.4 g, 45%), mp 116–120 °C, ^1^H NMR (600 MHz, CDCl_3_) δ(ppm): 7.4–7.35 (m, 2H, C*H*_arom_), 7.28–7.23 (m, 2H, C*H*_arom_), 7.22–7.18 (m, 2H, C*H*_arom_), 7.15–7.06 (m, 6H, C*H*_arom_), 6.88 (d, *J* = 8.5 Hz, 2H, C*H*_arom_), 6.73–6.67 (m, 3H, C*H*_arom_), 6.66 (d, *J* = 7.7 Hz, 2H, C*H*_arom_), 5.06 (d, *J* = 23.7 Hz, 1H, C*H*-P), 2.91 (s, 6H, N(C*H*_3_)_2_). ^13^C NMR (151 MHz, CDCl_3_) δ(ppm): 150.6, 150.6 (d, *J*_C-P_ = 6.6 Hz), 150.5 (d, *J*_C-P_ = 10.0 Hz), 146.3 (d, *J*_C-P_ = 15.4 Hz), 129.7, 129.7, 129.3, 129.1 (d, *J*_C-P_ = 6.3 Hz), 125.3, 125.2, 121.8, 120.9 (d, *J*_C-P_ = 4.1 Hz), 120.6 (d, *J*_C-P_ = 4.1 Hz), 118.7, 114.2, 112.8 (d, *J*_C-P_ = 2.1 Hz), 55.5 (d, *J*_C-P_ = 156.7 Hz), 40.63. ^31^P NMR (243 MHz, CDCl_3_) δ(ppm): 15.7. HRMS (ESI^+^) *m*/*z*, calcd. for C_27_H_28_N_2_O_3_P [M + H]^+^ 459.1838; found 459.1801.

Diphenyl ((phenylamino)(thiophen-2-yl)methyl)phosphonate (**12**)

The desired aminophosphonate **12** was obtained from aniline **3a** (0.5 g, 5.36 mmol), 2-thiophenecarboxaldehyde **2e** (0.6 g, 5.36 mmol) and diphenyl phosphite **4** (1.38 g, 5.9 mmol) as a white solid (1.92 g, 85%), mp 134–136 °C, ^1^H NMR (700 MHz, DMSO-*d*_6_) δ(ppm): 7.5–7.47 (m, 1H, C*H*_arom_), 7.4–7.37 (m, 1H, C*H*_arom_), 7.36–7.31 (m, 4H, C*H*_arom_), 7.2–7.16 (m, 2H, C*H*_arom_), 7.11–7.07 (m, 4H, C*H*_arom_), 7.04–7.02 (m, 1H, C*H*_arom_), 6.99–6.94 (m, 4H, C*H*_arom_), 6.66–6.60 (m, 2H, C*H*_arom_), 5.93 (dd, *J* = 25.1 Hz, 10.2 Hz, 1H, C*H*-P). ^13^C NMR (176 MHz, DMSO-*d*_6_) δ(ppm): 150.1 (d, *J*_C-P_ = 10.1 Hz), 1450.0 (d, *J*_C-P_ = 10.0 Hz), 146.8 (d, *J*_C-P_ = 13.3 Hz), 138.7, 129.8 (d, *J*_C-P_ = 13.4 Hz), 128.8, 127.6 (d, *J*_C-P_ = 7.7 Hz), 126.9 (d, *J*_C-P_ = 2.0 Hz), 126.35 (d, *J*_C-P_ = 3.3 Hz), 125.3, 120.6 (d, *J*_C-P_ = 3.7 Hz), 120.4 (d, *J*_C-P_ = 3.8 Hz), 117.6, 113.8, 50.5 (d, *J*_C-P_ = 164.3 Hz). ^31^P NMR (243 MHz, DMSO-*d*_6_) δ(ppm): 19.6. HRMS (ESI^+^) *m*/*z*, calcd. for C_23_H_21_NO_3_PS [M + H]^+^ 422.0980; found 422.0954.

(E)-diphenyl (1-((4-cyanophenyl)amino)-3-phenyl allyl)phosphonate (**13**)

The desired aminophosphonate **13** was obtained from 4-aminobenzonitrile **3c** (0.23 g, 1.95 mmol), cinnamaldehyde **2c** (0.26 g, 1.95 mmol) and diphenyl phosphite **4** (0.5 g, 2.14 mmol) as a white solid (0.71 g, 77%), mp 160–163 °C, ^1^H NMR (600 MHz, CDCl_3_) δ(ppm): 7.41 (d, *J* = 8.7 Hz, 2H, C*H*_arom_), 7.32–7.23 (m, 9H, C*H*_arom_), 7.20–7.13 (m, 4H, C*H*_arom_), 7.07 (d, *J* = 8.3 Hz, 2H, C*H*_arom_), 6.77 (dd, *J* = 15.9 Hz, 4.0 Hz, 1H, C*H*_vinylic_), 6.68 (d, *J* = 8.7 Hz, 2H, C*H*_arom_), 6.31 (dt, *J* = 15.9 Hz, 5.7 Hz, 1H, C*H*_vinylic_), 5.37–5.29 (m, 1H, N*H*), 4.87 (dt, *J =* 25.4 Hz, 6.7 Hz, 1H, C*H*-P). ^13^C NMR (151 MHz, CDCl_3_) δ(ppm): 150.2 (d, *J*_C-P_ = 9.9 Hz), 149.6 (d, *J*_C-P_ = 11.4 Hz), 135.6 (d, *J*_C-P_ = 2.9 Hz), 134.9, 134.8, 133.9, 130.1, 130.0, 128.8, 128.6, 126.8, (d, *J*_C-P_ = 1.8 Hz), 125.8, 120.8, 120.6 (d, *J*_C-P_ = 4.1 Hz), 120.6 (d, *J*_C-P_ = 4.3 Hz), 120.1, 113.5, 100.9, 53.3 (d, *J*_C-P_ = 156.8 Hz).^31^P NMR (243 MHz, CDCl_3_) δ(ppm): 13.7. HRMS (ESI^+^) *m*/*z*, calcd. for C_28_H_24_N_2_O_3_P [M + H]^+^ 467.1525; found 467.1496.

(E)-diphenyl(1-((4-cyanophenyl)amino)-3-(4-(dimethylamino)phenyl)allyl)phosphonate (**14**)

The desired aminophosphonate **14** was obtained from 4-aminobenzonitrile **3c** (0.16 g, 1.35 mmol), 4-(dimethylamino)cinnamaldehyde **2d** (0.2 g, 1.35 mmol) and diphenyl phosphite **4** (0.35 g, 1.49 mmol) as a purple solid (0.55 g, 81%), mp 150–154 °C, ^1^H NMR (600 MHz, CDCl_3_) δ(ppm): 7.44 (d, *J* = 8.7 Hz, 2H, C*H*_arom_), 7.31–7.25 (m, 4H, C*H*_arom_), 7.21 (d, *J* = 8.7 Hz, 2H, C*H*_arom_), 7.19–7.13 (m, 4H, C*H*_arom_), 7.09 (d, *J* = 8.3 Hz, 2H, C*H*_arom_), 6.71–6.61 (m, 5H, C*H*_arom_), 6.05 (dt, *J* = 15.8 Hz, 12.3 Hz, 1H, C*H*_vinylic_), 4.98 (t, *J* = 7.7 Hz, 1H, N*H*), 4.82 (dt, *J* = 24.4 Hz, 7.4 Hz, 1H, C*H*-P), 2.96 (s, 6H, N(C*H*_3_)_2_). ^13^C NMR (151 MHz, CDCl_3_) δ(ppm): 150.6, 150.2 (d, *J*_C-P_ = 12.2 Hz), 149.6 (d, *J*_C-P_ = 11.4 Hz), 135.1, 135.0, 133.7, 129.9, 129.8, 127.8, 125.6, 125.5, 123.7, 120.6 (d, *J*_C-P_ = 3.4 Hz), 120.5 (d, *J*_C-P_ = 3.6 Hz), 119.9, 115.4 (d, *J*_C-P_ = 3.8 Hz), 113.5, 112.1, 100.7, 53.4 (d, *J*_C-P_ = 157.5 Hz), 40.3. ^31^P NMR (243 MHz, CDCl_3_) δ(ppm): 10.7. HRMS (ESI^+^) *m*/*z*, calcd. for C_30_H_29_N_3_O_3_P [M + H]^+^ 510.1947; found 510.1939.

Diphenyl (((4-cyanophenyl)amino)(4-(dimethylamino)phenyl)methyl)phosphonate (**15**)

The desired aminophosphonate **15** was obtained from 4-aminobenzonitrile **3c** (0.22 g, 1.86 mmol), 4-(dimethylamino)benzaldehyde **2a** (0.28 g, 1.86 mmol) and diphenyl phosphite **4** (0.5 g, 2.06 mmol) as a yellow solid (0.76 g, 85%), mp 165–168 °C, ^1^H NMR (600 MHz, CDCl_3_) δ(ppm): 7.37–7.34 (m, 2H, C*H*_arom_), 7.32 (d, *J* = 8.8 Hz, 2H, C*H*_arom_), 7.28–7.24 (m, 2H, C*H*_arom_), 7.21–7.17 (m, 2H, C*H*_arom_), 7.17–7.13 (m, 1H, C*H*_arom_), 7.12–7.07 (m, 3H, C*H*_arom_), 6.79 (d, *J* = 8.5 Hz, 2H, C*H*_arom_), 6.65 (d, *J* = 8.6 Hz, 2H, C*H*_arom_), 6.6 (d, *J* = 8.8 Hz, 2H, C*H*_arom_), 5.86 (t, *J* = 8.1 Hz, 1H, N*H*), 5.05 (dd, *J* = 23.7 Hz, 8.0 Hz, 1H, C*H*-P), 2.92 (s, 6H, N(CH_3_)_2_). ^13^C NMR (151 MHz, CDCl_3_) δ(ppm): 150.7, 150.2 (d, *J*_C-P_ = 5.5 Hz), 150.2 (d, *J*_C-P_ = 5.1 Hz), 149.8 (d, *J*_C-P_ = 14.5 Hz), 133.5, 129.8, 129.6, 129.0 (d, *J*_C-P_ = 6.1 Hz), 125. 5, 125.2, 120.6 (d, *J*_C-P_ = 4.1 Hz), 120.4 (d, *J*_C-P_ = 4.2 Hz), 120.3, 120.1, 113.5, 112.6, 100.1, 54.6 (d, *J*_C-P_ = 157.9 Hz), 40.4. ^31^P NMR (243 MHz, CDCl_3_) δ(ppm): 11.4. HRMS (ESI^+^) *m*/*z*, calcd. for C_28_H_27_N_3_O_3_P [M + H]^+^ 484.1790; found 484.1794.

Diphenyl (((4-cyanophenyl)amino)(4-(diethylamino)phenyl)methyl)phosphonate (**16**)

The desired aminophosphonate **16** was obtained from 4-aminobenzonitrile **3c** (0.22 g, 1.86 mmol), 4-(diethylamino)benzaldehyde **2b** (0.34 g, 1.86 mmol) and diphenyl phosphite **4** (0.5 g, 2.06 mmol) as a pale yellow solid (0.67, 68%), mp 168–171 °C, ^1^H NMR (600 MHz, CDCl_3_) δ(ppm): 7.32 (d, *J* = 8.4 Hz, 4H, C*H*_arom_), 7.28–7.23 (m, 2H, C*H*_arom_), 7.19–7.12 (m, 3H, C*H*_arom_), 7.12–7.07 (m, 3H, C*H*_arom_), 6.74 (d, *J* = 8.0 Hz, 2H, C*H*_arom_), 6.61 (d, *J* = 8.6 Hz, 2H, C*H*_arom_), 6.57 (d, *J* = 8.4 Hz, 2H, C*H*_arom_), 5.92 (t, *J* = 8.2 Hz, 1H, N*H*), 5.04 (dd, *J* = 23.4 Hz, 8.6 Hz, 1H, C*H*-P), 3.31 (q, *J* = 6.9 Hz, 4H, N(C*H*_2_CH_3_)_2_), 1.1 (t, *J* = 7.0 Hz, 6H, N(CH_2_C*H*_3_)_2_). ^13^C NMR (151 MHz, CDCl_3_) δ(ppm): 150.4 (d, *J*_C-P_ = 5.9 Hz), 150.3 (d, *J*_C-P_ = 6.2 Hz), 149.9 (d, *J*_C-P_ = 14.2 Hz), 148.1, 133.6, 129.9, 129.6, 129.4 (d, *J*_C-P_ = 6.2 Hz), 125.5, 125.3, 120.7 (d, *J*_C-P_ = 4.2 Hz), 120.5 (d, *J*_C-P_ = 4.3 Hz), 120.3, 119.1, 113.6, 112.0, 100.1, 54.8 (d, *J*_C-P_ = 158.1 Hz), 44.4, 12.6. ^31^P NMR (243 MHz, CDCl_3_) δ(ppm): 11.7. HRMS (ESI^+^) *m*/*z*, calcd. for C_30_H_31_N_3_O_3_P [M + H]^+^ 512.2103; found 512.2077.

### 3.2. Synthesis of α-Aminophosphonic Acids **17**–**28**

In a 30 mL microwave (Anton Paar Monowave 300) vial with a magnetic stirrer, 1 eq. of the corresponding α-aminophosphonate **5**–**16**, 1.1 eq. of potassium carbonate (K_2_CO_3_) and 3 mL of an ethanol/water mixture (3:1) were added. The vial was placed in the microwave cavity and reacted at 140 °C for 20 min. The solvents were evaporated under reduced pressure, and the product was purified by column chromatography with AcOEt/MeOH (3:1). This method was used for the synthesis of all the following molecules.

(E)-ethyl 4-((1-(hydroxy(phenoxy)phosphoryl)-3-phenylallyl)amino)benzoate (**17**)

The desired aminophosphonic acid **17** was obtained from compound **5** (0.5 g, 0.97 mmol) and potassium carbonate (0.15 g, 1.1 mmol) as a pale yellow solid (0.13 g, 30%), mp 220–223 °C, ^1^H NMR (600 MHz, DMSO-*d*_6_) δ(ppm): 7.6 (d, *J* = 8.0 Hz, 2H, C*H*_arom_), 7.29–7.21 (m, 4H, C*H*_arom_), 7.20–7.12 (m, 5H, C*H*_arom_, C*H*_vinylic_), 6.98–6.93 (m, 1H, C*H*_vinylic_), 6.66 (d, *J* = 7.9 Hz, 2H, C*H*_arom_), 6.47–6.40 (m, 2H, C*H*_arom_), 6.28 (s, 1H, N*H*), 4.28 (d, *J* = 24.6 Hz, 1H, C*H*-P), 4.17 (q, *J* = 6.9 Hz, 2H, C*H*_2_CH_3_), 1.24 (t, *J* = 7.1 Hz, 3H, CH_2_C*H*_3_). ^13^C NMR (151 MHz, DMSO-*d*_6_) δ(ppm): 165.9, 153.4 (d, *J*_C-P_ = 5.3 Hz), 152.3 (d, *J*_C-P_ = 11.3 Hz), 137.0, 130.7, 128.9, 128.7, 128.5, 128.0 (d, *J*_C-P_ = 8.8 Hz), 127.0, 126.0, 122.4, 120.8 (d, *J*_C-P_ = 3.7 Hz), 116.4, 111.8, 59.5, 53.9 (d, *J*_C-P_ = 143.0 Hz), 14.38. ^31^P NMR (243 MHz, DMSO-*d*_6_) δ(ppm): 10.7. HRMS (ESI^+^) *m*/*z*, calcd. for C_24_H_25_NO_5_P [M + H]^+^ 438.1470; found 438.1445.

(E)-ethyl 4-((3-(4-(dimethylamino)phenyl)-1-(hydroxy(phenoxy)phosphoryl)allyl) amino)benzoate (**18**)

The desired aminophosphonic acid **18** was obtained from compound **6** (0.4 g, 0.72 mmol) and potassium carbonate (0.11 g, 0.79 mmol) as a red solid (0.12 g 37%), mp 316–320 °C, ^1^H NMR (600 MHz, DMSO-*d*_6_) δ(ppm): 7.39–7.17 (m, 3H, C*H*_arom_), 6.79 (m, 4H, C*H*_arom_, C*H*_vinylic_), 6.65–6.56 (m, 2H, C*H*_arom_), 6.31–6.17 (m, 3H, C*H*_arom_), 6.01–5.75 (m, 3H, C*H*_arom_, C*H*_vinylic_). ^13^C NMR (151 MHz, DMSO-*d*_6_) δ(ppm): 165.9, 153.5 (d, *J*_C-P_ = 9.7 Hz), 152.5 (d, *J*_C-P_ = 11.2 Hz), 149.5, 131.1, 130.7, 128.9, 126.9, 125.3 (d, *J*_C-P_ = 2.5 Hz), 122.9, 122.2, 120.8, 116.1, 112.7, 112.2, 59.5, 53.9 (d, *J*_C-P_ = 140.9 Hz), 40.1, 14.4. ^31^P NMR (243 MHz, DMSO-*d*_6_) δ(ppm): 9.31. HRMS (ESI^+^) *m*/*z*, calcd. for C_26_H_30_N_2_O_5_P [M + H]^+^481.1892, found 481.1870.

Ethyl 4-(((4-(dimethylamino)phenyl)(hydroxy(phenoxy)phosphoryl)methyl)amino)benzoate (**19**)

The desired aminophosphonic acid **19** was obtained from compound **7** (0.3 g, 0.56 mmol) and potassium carbonate (0.085 g, 0.62 mmol) as a pale yellow solid (0.12 g, 45%), mp 210–214 °C, ^1^H NMR (600 MHz, DMSO-*d*_6_) δ(ppm): 7.54 (d, *J* = 8.5 Hz, 2H, C*H*_arom_), 7.19–7.12 (m, 4H, C*H*_arom_), 7.02 (d, *J* = 7.9 Hz, 2H, C*H*_arom_), 6.93–6.87 (m, 1H, C*H*_arom_), 6.58 (d, *J* = 8.3 Hz, 2H, C*H*_arom_), 6.53 (d, *J* = 7.3 Hz, 2H, C*H*_arom_), 6.4–6.34 (m, 1H, N*H*), 4.31 (dd, *J* = 21.3 Hz, 6.2 Hz, 1H, C*H*-P), 4.16 (q, *J* = 6.2 Hz, 2H, C*H*_2_CH_3_), 2.81 (s, 6H, N(C*H*_3_)_2_), 1.23 (t, *J* = 7.0 Hz, 3H, CH_2_C*H*_3_). ^13^C NMR (151 MHz, DMSO-*d*_6_) δ(ppm): 165.9, 152.5, 152.4, 149.1, 130.6, 128.7, 128.4 (d, *J*_C-P_ = 4. 3 Hz), 127.9, 121.6, 120.7 (d, *J*_C-P_ = 3.7 Hz), 115.9, 112.1, 111.7, 59.5, 55.1 (d, *J*_C-P_ = 138.4 Hz), 40.5, 14.4. ^31^P NMR (243 MHz, DMSO-*d*_6_) δ(ppm): 10.9. HRMS (ESI^+^) *m*/*z*, calcd. for C_24_H_28_N_2_O_5_ [M + H]^+^ 455.1736; found 455.1707.

Ethyl 4-(((4-(diethylamino)phenyl)(hydroxy(phenoxy)phosphoryl)methyl)amino) benzoate (**20**)

The desired aminophosphonic acid **20** was obtained from compound **8** (0.3 g, 0.54 mmol) and potassium carbonate (0.082 g, 0.59 mmol) as a pale yellow solid (0.12 g, 45%), mp 218–220 °C, ^1^H NMR (600 MHz, DMSO-*d*_6_) δ(ppm): 7.55–748 (m, 2H, C*H*_arom_), 7.17–7.07 (m, 4H, C*H*_arom_), 7.01–6.93 (m, 2H, C*H*_arom_), 6.91–6.84 (m, 1H, C*H*_arom_), 6.54–6.47 (m, 2H, C*H*_arom_), 6.46 (d, *J* = 8.5 Hz, 2H, C*H*_arom_), 6.4–6.36 (m, 1H, N*H*), 4.29 (d, *J* = 20.6 Hz, 1H, C*H*-P), 4.12 (m, 3H,-O*H*, C*H*_2_CH_3_), 3.21 (m, 4H, N(C*H*_2_CH_3_)_2_), 1.2 (t, *J* = 7.0 Hz, 3H, CH_2_C*H*_3_), 0.98 (m, 6H, N(CH_2_C*H*_3_)_2_). ^13^C NMR (151 MHz, DMSO-*d*_6_) δ(ppm): 167.0, 152.4 (d, *J*_C-P_ = 3.3 Hz), 145.9, 131.7, 131.6, 131.1, 130.6, 126.2 (d, *J*_C-P_ = 4.0 Hz), 121.7, 120.7, 115.9, 112.7, 112.4 (d, *J*_C-P_ = 4.1 Hz), 59.4, 55.0 (d, *J*_C-P_ = 140.0 Hz), 43.7, 14.4, 12.6. ^31^P NMR (243 MHz, DMSO-*d*_6_) δ(ppm): 8.6. HRMS (ESI^+^) *m*/*z*, calcd. for C_26_H_32_N_2_O_5_P [M + H]^+^ 483.2049; found 483.2028

Ethyl 4-(((hydroxy(phenoxy)phosphoryl)(thiophen-2-yl)methyl)amino)benzoate (**21**)

The desired aminophosphonic acid **21** was obtained from compound **9** (0.2 g, 0.41 mmol) and potassium carbonate (0.062 g, 0.44 mmol) as a pale yellow solid (0.13 g, 76%), mp 232–236 °C, ^1^H NMR (600 MHz, DMSO-*d*_6_) δ(ppm): 7.72 (d, *J* = 8.6 Hz, 1H, C*H*_arom_), 7.37–7.3 (m, 3H, C*H*_arom_), 7.23 (d, *J* = 8.6 Hz, 2H, C*H*_arom_), 7.18–7.11 (m, 3H, C*H*_arom_), 7.09–7.06 (m, 1H, C*H*_arom_), 6.96 (d, *J* = 8.7 Hz, 1H, C*H*_arom_), 6.68 (d, *J* = 8.7 Hz, 1H, C*H*_arom_), 6.11 (t, *J* = 6.2 H, 1H, N*H*), 5.31 (dt, *J* = 21.2 Hz, 8.0 Hz, 1H, C*H*-P), 4.21 (q, *J* = 7.2 Hz, 2H, C*H*_2_CH_3_), 1.27 (t, *J* = 7.1 Hz, 3 H, CH_2_C*H*_3_). ^13^C NMR (176 MHz, DMSO-*d*_6_) δ(ppm): 165. 7, 151.3 (d, *J*_C-P_ = 7.4 Hz), 150.1 (d, *J*_C-P_ = 3.2 Hz), 133.7 (d, *J*_C-P_ = 13.6 Hz), 131.0, 130.7, 129.9 (d, *J*_C-P_ = 17.3 Hz), 127.4, 120.6 (d, *J*_C-P_ = 3.6 Hz), 117.7, 115.2, 112.38, 112.21, 59.7, 52.8 (d, *J*_C-P_ = 157.7 Hz), 14.35. ^31^P NMR (243 MHz, DMSO-*d*_6_) δ(ppm): 10.86. HRMS (ESI^+^) *m*/*z*, calcd. for C_20_H_21_NO_5_PS [M + H]^+^ 418.0878; found 418.0870.

(E)-phenyl hydrogen (3-phenyl-1-(phenylamino)allyl)phosphonate (**22**)

The desired aminophosphonic acid **22** was obtained from compound **10** (0.3 g, 0.68 mmol) and potassium carbonate (0.1 g, 0.75 mmol) as a brown solid (0.1 g, 42%), mp 240–243 °C, ^1^H NMR (700 MHz, DMSO-*d*_6_) δ(ppm): 7.29 (m, 4H, C*H*_arom_), 7.21–7.12 (m, 5H, C*H*_arom_) 7.01–6.92 (m, 3H, C*H*_arom_), 6.6–6.54 (m, 2H, C*H*_arom_), 6.52–6.54 (m, 1H, C*H*_arom_), 6.45–6.41(m, 2H, C*H*_arom_) 5.29 (s, 1H, N*H*), 4.16 (d, *J* = 23.39 Hz, 1H, C*H*-P). ^13^C NMR (176 MHz, DMSO-*d*_6_) δ(ppm): 153.5, 148.2 (d, *J*_C-P_ = 12.1 Hz), 137.2, 129.1, 128.8, 128.7, 128.4, 128.2, 126.8, 126.0, 122.3, 120.9 (d, *J*_C-P_ = 3.5 Hz), 115.9, 112.7, 54.5 (d, *J*_C-P_ = 142.8 Hz). ^31^P NMR (243 MHz, DMSO-*d*_6_) δ(ppm): 14.3. HRMS (ESI^+^) *m*/*z*, calcd. for C_21_H_21_NO_3_P [M + H]^+^ 366.1259; found 366.1266.

Phenyl hydrogen ((phenylamino)(thiophen-2-yl)methyl)phosphonate (**23**)

The desired aminophosphonic acid **23** was obtained from compound **12** (0.5 g, 1.2 mmol) and potassium carbonate (0.18 g, 1.3 mmol) as a white solid (0.26 g, 64%), mp 240–244 °C, ^1^H NMR (600 MHz, DMSO-*d*_6_) δ(ppm): 7.21–7.12 (m, 3H, C*H*_arom_), 7.08–7.02 (m, 3H, C*H*_arom_), 7.01–6.96 (m, 2H, C*H*_arom_), 6.96–6.91 (m, 1H, C*H*_arom_), 6.88–6.83 (m, 1H, C*H*_arom_), 6.56 (d, *J* = 7.4 Hz, 2H, C*H*_arom_), 6.53–6.48 (m, 1H, C*H*_arom_), 5.6–5-52 (m, 1H, N*H*), 4.68 (dd, *J* = 23.6 Hz, 6.1 Hz, 1H, C*H*-P). ^13^C NMR (176 MHz, DMSO-*d*_6_) δ(ppm): 153.6 (d, *J*_C-P_ = 5.9 Hz), 148.0 (d, *J*_C-P_ = 11.9 Hz), 145.4, 128.8, 128.7, 126.3, 124.6, 123.3, 122.1, 120.7 (d, *J*_C-P_ = 3.4 Hz) 116.4, 112.9, 52.7 (d, *J*_C-P_ = 144.0 Hz). ^31^P NMR (243 MHz, DMSO-*d*_6_) δ(ppm): 9.2 HRMS (ESI^+^) *m*/*z*, calcd. for C_17_H_17_NO_3_PS [M + H]^+^ 346.0667; found 346.0642.

(E)-phenyl hydrogen (1-((4-cyanophenyl)amino)-3-phenylallyl)phosphonate (**24**)

The desired aminophosphonic acid **24** was obtained from compound **13** (0.3 g, 0.64 mmol) and potassium carbonate (0.097 g, 0.71 mmol) as a yellow solid (0.1 g, 41%), mp 290–294 °C, ^1^H NMR (600 MHz, DMSO-*d*_6_) δ(ppm): 7.36–7.30 (m, 2H, C*H*_arom_), 7.29–7.21 (m, 4H, C*H*_arom_), 7.20–7.12 (m, 4H, C*H*_arom_), 6.98–6.94 (m, 1H, C*H*_arom_), 6.72 (d, *J* = 8.3 Hz, 2H, C*H*_arom_), 6.65–6.54 (m, 1H, C*H*_arom_) 6.47–6.38 (m, 2H, C*H*_arom_), 4.25 (d, *J* = 24.3 Hz, 1H, C*H*-P). ^13^C NMR (151 MHz, DMSO-*d*_6_) δ(ppm): 153.4 (d, *J*_C-P_ = 7.2 Hz), 151.9 (d, *J*_C-P_ = 10.9 Hz), 136.9 (d, *J*_C-P_ = 2.1 Hz), 133.1, 128.9, 128.6, 128.5, 127.8, 127.0, 126.0, 122.3, 120.8 (d, *J*_C-P_ = 3.8 Hz), 120.6, 112.6, 95.7, 53.8 (d, *J*_C-P_ = 142.4 Hz). ^31^P NMR (243 MHz, DMSO-*d*_6_) δ(ppm): 8.1 HRMS (ESI^+^) *m*/*z*, calcd. for C_22_H_20_N_2_O_3_P [M + H]^+^ 391.1212; found 391.1206.

(E)-phenyl hydrogen (1-((4-cyanophenyl)amino)-3-(4-(dimethylamino)phenyl)allyl) phosphonate (**25**)

The desired aminophosphonic acid **25** was obtained from compound **14** (0.18 g, 0.35 mmol) and potassium carbonate (0.054 g, 0.38 mmol) as a yellow solid (0.1 g, 67%), mp 270–273 °C, ^1^H NMR (600 MHz, DMSO-*d*_6_) δ(ppm): 7.33 (d, *J* = 8.4 Hz, 2H, C*H*_arom_), 7.21–7.15 (m, 2H, C*H*_arom_), 7.14–7.08 (m, 4H, C*H*_arom_), 6.98–6.93 (m, 1H, C*H*_arom_), 6.7 (d, *J* = 8.3 Hz, 2H, C*H*_arom_), 6.59 (d, *J* = 8.3 Hz, 2H, C*H*_arom_), 6.48 (s, 1H, N*H*), 6.29 (dd, *J* = 16.2 Hz, 3.9 Hz, 1H, C*H*_vinylic_), 6.13 (dd, *J* = 15.7 Hz, 4.6 Hz, 1H, C*H*_vinylic_), 4.17 (dt, *J* = 24.2 Hz, 7.9 Hz, 1H, C*H*-P), 2.85 (s, 6H, N(C*H*_3_)_2_). ^13^C NMR (151 MHz, DMSO-*d*_6_) δ(ppm): 153.5, 152.0 (d, *J*_C-P_ = 10.6 Hz), 149.5, 133.0, 128.9, 126.9, 125.1, 122.5, 122.2, 120.8 (d, *J*_C-P_ = 3.7 Hz), 120.7, 112.6, 112.6, 112.2, 95.5, 53.8 (d, *J*_C-P_ = 143.3 Hz), 39.9. ^31^P NMR (243 MHz, DMSO-*d*_6_) δ(ppm): 9.1. HRMS (ESI^+^) *m*/*z*, calcd. for C_24_H_25_N_3_O_3_P [M + H]^+^ 434.1634; found 434.1619.

Phenyl hydrogen (((4-cyanophenyl)amino)(4-(dimethylamino)phenyl)methyl)phosphonate (**26**)

The desired aminophosphonic acid **26** was obtained from compound **15** (0.3 g, 0.62 mmol) and potassium carbonate (0.094 g, 0.68 mmol) as a white solid (0.2 g, 75%), mp 258–261 °C, ^1^H NMR (600 MHz, CD_3_OD/DMSO-*d*_6_) δ(ppm): 7.3 (d, *J* = 7.8 Hz, 2H, C*H*_arom_), 7.22–7.12 (m, 4H, C*H*_arom_), 7.03–6.97 (m, 2H, C*H*_arom_), 6.95–6.89 (m, 1H, C*H*_arom_), 6.62–6.52 (m, 4H, C*H*_arom_), 4.36 (d, *J* = 22.0 Hz, 1H, C*H*-P), 2.81 (s, 6H, N(C*H*_3_)_2_). ^13^C NMR (151 MHz, CD_3_OD/DMSO-*d*_6_) δ(ppm): 153.9 (d, *J*_C-P_ = 7.3 Hz), 151.9 (d, *J*_C-P_ = 12.9 Hz), 149.5, 133.2, 129.0, 128.7 (d, *J*_C-P_ = 4.2 Hz), 127.0, 122.2, 120.9 (d, *J*_C-P_ = 3.5 Hz), 120.8, 112.8, 112.3, 96.0, 55.1 (d, *J*_C-P_ = 142.1 Hz), 40.5. ^31^P NMR (243 MHz, CD_3_OD/DMSO-*d*_6_) δ(ppm): 10.5. HRMS (ESI^+^) *m*/*z*, calcd. for C_22_H_23_N_3_O_3_P [M + H]^+^ 408.1477; found 408.1452.

Phenyl hydrogen (((4-cyanophenyl)amino)(4-(diethylamino)phenyl)methyl) phosphonate (**27**)

The desired aminophosphonic acid **27** was obtained from compound **16** (0.15 g, 0.29 mmol) and potassium carbonate (0.044 g, 0.32 mmol) as a brown solid (0.079 g, 62%), mp 248–250 °C, ^1^H NMR (600 MHz, DMSO-*d*_6_) δ(ppm): 7.98 (d, *J* = 8.5 Hz, 2H, C*H*_arom_), 7.87 (d, *J* = 8.2 Hz, 2H, C*H*_arom_), 7.85–7.80 (m, 2H, C*H*_arom_), 7.71 (d, *J* = 8.0 Hz, 2H, C*H*_arom_), 7.63–7.58 (m, 1H, C*H*_arom_), 7.33–7.26 (m, 2H, C*H*_arom_), 7.16 (d, *J* = 8.3 Hz, 2H, C*H*_arom_), 5.03 (dd, *J* = 22.1 Hz, 7.3 Hz, 1H, C*H*-P), 3.93 (q, *J* = 6.7 Hz, 4H, N(C*H*_2_CH_3_)_2_), 1.72 (t, *J* = 7.0 Hz, 6H, N(CH_2_C*H*_3_)_2_). ^13^C NMR (151 MHz, DMSO-*d*_6_) δ(ppm): 153.7 (d, *J*_C-P_ = 8.0 Hz), 152.0 (d, *J*_C-P_ = 12.7 Hz), 146.0, 132.93, 128.9 (d, *J*_C-P_ = 4.1 Hz), 128.7, 125.6, 121.9, 120.8, 120.7 (d, *J*_C-P_ = 3.7 Hz), 112.7, 111.0, 95.3, 55.0 (d, *J*_C-P_ = 143.3 Hz), 43.6, 12.6. ^31^P NMR (243 MHz, DMSO-*d*_6_) δ(ppm): 10.46. HRMS (ESI^+^) *m*/*z*, calcd. for C_24_H_27_N_3_O_3_P [M + H]^+^ 436.1790; found 436.1772.

Phenyl hydrogen ((4-(dimethylamino)phenyl)(phenylamino)methyl)phosphonate (**28**)

The desired aminophosphonic acid **28** was obtained from compound **11** (0.2 g, 0.43 mmol) and potassium carbonate (0.066 g, 0.47 mmol) as a white solid (0.066, 40%), mp 256–260 °C, ^1^H NMR (600 MHz, DMSO-*d*_6_) δ(ppm): 7.73–7.64 (m, 1H, C*H*_arom_), 7.22 (d, *J* = 8.1 Hz, 2H, C*H*_arom_), 7.18–7.12 (m, 2H, C*H*_arom_), 7.06 (d, *J* = 7.9 Hz, 2H, C*H*_arom_), 6.95–6.89 (m, 3H, C*H*_arom_), 6.57 (d, *J* = 8.2 Hz, 2H, C*H*_arom_), 6.45 (d, *J* = 8.2 Hz, 1H, C*H*_arom_), 6.44–6.39 (m, 1H, C*H*_arom_), 5.52 (s, 1H, N*H*), 4.3 (d, *J* = 20.4 Hz, 1H, N*H*-P), 2.8 (s, 6H, N(C*H*_3_)_2_). ^13^C NMR (151 MHz, DMSO-*d*_6_) δ(ppm): 167.0, 149.0, 148.2 (d, *J*_C-P_ = 13.4 Hz), 131.7, 131.6, 128.7, 128.6, 121.8, 120.7 (d, *J*_C-P_ = 3.5 Hz), 115.5, 112.8, 112.1, 55.5 (d, *J*_C-P_ = 142.2 Hz), 40.5. ^31^P NMR (243 MHz, DMSO-*d*_6_) δ(ppm): 10.86.

### 3.3. In Vitro Antifungal Activity

A total of five isolates of the *L. prolificans* were tested: 11-2242, 05-2190, 10-1167, 13-196 and 09-1125. All isolates were obtained from the Microbiology Department of the School of Medicine of Universidad Autónoma de Nuevo León and were previously identified by Elizondo-Zertuche et al. [45]. The strains were incubated on potato dextrose agar (PDA) at 35 °C for their metabolic reactivation.

For the in vitro susceptibility studies, a preliminary test was carried out by the agar diffusion method, and all synthesized compounds (α-aminophosphonates **5**–**16** and monohydrolyzed α-aminophosphonic acids **17**–**28**) were evaluated against three strains of the *Lomentospora* (11-2242, 10-1167, 09-1125); thus, the most active compounds were selected based on the diameter of the growth inhibition zones. For this, conidia were inoculated as a lawn on Müller–Hinton medium (MCD Lab) with a concentration of 0.4 × 10^4^–5 × 10^4^ conidia/mL, and the disks were impregnated with each of the 24 compounds at a concentration of 1.0 mg/mL. Inhibition halos were compared to VRC. Subsequently, the most active compounds, **7**, **11**, **13**, **22** and **27**, were evaluated at different concentrations using the liquid medium susceptibility method against the three *Lomentospora* strains previously mentioned (11-2242, 10-1167, 09-1125). Then, 5 mL snap tubes and 900 mL of nutrient broth (BD Bioxon) were added at a concentration of 0.4 × 10^4^–5 × 10^4^ conidia/mL, and 100 mL of the compounds was added at concentrations between 100 and 1000 mg/mL. The tubes were vortexed and incubated at 35 °C for 72 h, and the concentration range was determined for subsequent evaluation by the CLSI method M38-A2.

Finally, the in vitro antifungal susceptibility of the five strains of *Lomentospora* species was determined by the macrodilution method of the CLSI M38-A2 protocol. The reference antifungal agent used was VRC, which was obtained as pure reagent grade powder. The final drug concentrations ranged from 0.125 to 64 μg/mL for VRC and from 650 to 1000 μg/mL for compounds **7**, **11**, **13**, **22** and **27**. The tubes were incubated at 37 °C for 72 h, and the minimal inhibitory concentration (MIC) for all antifungals was registered visually. The MIC was defined as the drug concentration for which a 100% reduction in turbidity compared with the drug-free control for voriconazole and the compounds **7**, **11**, **13**, **22** and **27** were observed. Assays were carried out in duplicate using *Candida parapsilosis* ATCC 22019 as quality control organisms.

### 3.4. Evaluation of Cytotoxic Activity

#### 3.4.1. Cell Culture

The monkey kidney cell line (COS-7) used for biological tests was obtained from Centro Médico Siglo XXI.

The cell line was cultured in RPMI-1640 culture medium supplemented with fetal bovine serum (10%) and an antibiotic-antifungal mixture (1%).

#### 3.4.2. MTT Assay

Active compounds (**7**, **11**, **13**, **22** and **27**) against *L. prolificans* and VRC were evaluated by an MTT assay to evaluate their cytotoxicity in healthy COS-7 cells. Briefly, the cells were harvested with EDTA trypsin solution, already detached from the base of the culture flask, harvested and diluted with supplemented medium to inactivate trypsin. An aliquot of cells was taken to perform the viability count through the trypan blue technique using an electronic counter. The inoculum density was adjusted to 10 × 10^4^ cells/mL, and they were deposited at a volume of 100 µL in 96-well plates. The cell cultures were incubated for 24 h to promote their adherence to the substrate at the bottom of the well. Next, the compounds were added in the solution with supplemented medium and their corresponding solvent at a volume of 100 µL. The compounds were prepared at a concentration of 40 mM in DMSO and/or ethanol; the final concentration was 1000 μM for the test drugs and ≤1% for DMSO. The etoposide was prepared at 20 μM in DMSO. At 24 h, the cell was removed from the medium and washed with a phosphate buffer, 100 µL of an MTT solution (3-(4,5-dimethylthiazol-2-yl)-2,5-diphenyltetrazolium) was immediately added and they were left for 4 h in the incubator. Afterwards, the MTT solution was removed, and 100 µL of DMSO was added to favor the solubility of formazan produced during the cell metabolism; optical density (OD) was measured in a microplate reader at a wavelength of 470 nm.

Cell viability was calculated from the following expression% Viability = (OD treatment/OD vehicle) × 100. Data are processed individually or by an independent experiment, obtaining the average of these, plus the standard error of the mean.

## 4. Conclusions

The microwave-assisted KF reaction proceeded in 10 min with moderate to good yields and without employing catalysts. The characteristic H-P and C-P coupling constants corroborated the formation of the 12 new α-aminophosphonates and 12 new α-aminophosphonic acids. When evaluating the antifungal activity of the 24 synthesized compounds against *L. prolificans* by the CLSI protocol M38-A2, compounds **7**, **11**, **13**, **22** and **27** exhibited in vitro antifungal activity against five strains of *L. prolificans*, and the molecular docking studies with the enzyme lanosterol-14-alpha-demethylase suggest that these compounds interact in an allosteric cavity. From the docking and SAR analysis, it was concluded that, in the design of new α-aminophosphonates and α-aminophosphonic acids with possible antifungal activity, it is recommended to consider the inclusion of certain structural features such as the phosphate group, aromatic rings substituted with electron-donating and sterically hindered groups at the R_1_ position and electro-withdrawing groups at the R_2_ position.

Compounds **7**, **11**, **13**, **22** and **27** are active only at higher concentrations if compared with voriconazole. On the other hand, they are still promising structures as starting scaffolds in the design of future derivatives with potential antifungal activity.

## Figures and Tables

**Table 1:** Coupling constants for compounds **5**–**28**.

Comp	*J*_H-P_	*J*_C-P_	Comp	*J*_H-P_	*J*_C-P_	Comp	*J*_H-P_	*J*_C-P_	Comp	*J*_H-P_	*J*_C-P_
***5***	25.85	156.26	**11**	23.73	156.74	**17**	24.6	142.97	**23**	20.36	142.24
***6***	25.72	157.1	**12**	25.12	164.28	**18**	22.2	140.86	**24**	23.6	144.0
***7***	23.5	157.07	**13**	25.36	156.82	**19**	21.31	138.44	**25**	24.26	142.4
***8***	23.31	157.47	**14**	24.37	157.53	**20**	20.62	140.06	**26**	24.25	143.34
***9***	24.02	162.6	**15**	23.65	157.9	**21**	21.23	157.7	**27**	21.98	142.1
***10***	26.11	155.71	**16**	23.44	158.11	**22**	23.39	142.8	**28**	20.36	142.24

**Table 2:** In vitro susceptibilities of five strains of *L. prolificans* against compounds **7**, **11**, **13**, **22** and **27** and antifungal VRC.

Strain	MIC_100_ Range (μg/mL) Compound 7	MIC_100_ Range (μg/mL) Compound 11	MIC_100_ Range (μg/mL) Compound 13	MIC_100_ Range (μg/mL) Compound 22	MIC_100_ Range (μg/mL) Compound 27	MIC_100_ Range (μg/mL) VRC
*L. prolificans (5)*	900->900	900->900	900->900	900->900	900->900	16->16

**Table 3:** Cytotoxicity profile of compounds **7**, **11**, **13**, **22** and **27** and the antifungal voriconazole.

Compound (1000 µM)	Viability (%)
**7**	39.01 ± 2.45
**11**	38.72 ± 1.31
**13**	41.85 ± 3.13
**22**	67.91 ± 4.85
**27**	60.26 ± 3.15
Voriconazole	68.55 ± 2.05
Etoposide *	40.79 ± 2.56

* Control (100 μM).

## Data Availability

Data available on request due to restrictions privacy. The data presented in this study are available on request from the corresponding author.

## Supplementary Materials

The following supporting information can be downloaded at: https://www.mdpi.com/article/10.3390/molecules28103995/s1, Figure S1: Comparison of 3D Structural similarities of voriconazole and active compounds **7**, **11**, **13**, **22** and **27**; Figure S2–S93: The characterization spectrum of synthesized compounds.

## Sample Availability

Samples of the compounds **5**–**28** are available from the authors.
