# Supplementary material for: Microwave-Assisted Synthesis of Aminophosphonic Derivatives and Their Antifungal Evaluation against Lomentospora prolificans
Source: Molecules. 2023 May 10;28(10):3995. doi: 10.3390/molecules28103995 (PMC10224343; doi:10.3390/molecules28103995)
Supplement: Supplementary file 1 [file molecules-28-03995-s001.zip › molecules-2332038-supplementary.pdf]

Supporting Information

Microwave-Assisted Synthesis of Aminophosphonic Derivatives and Their Antifungal Evaluation against *Lomentospora prolificans.*

Zuleyma Martínez-Campos[1], Mariana-Elizondo-Zertuche[2], Emanuel Hernández-Núñez[3], Eugenio Hernández-Fernández[1*], Efrén Robledo-Leal[4*], and Susana T. López-Cortina[1*]

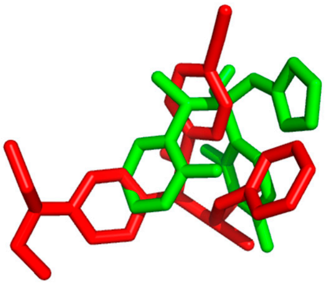

a)

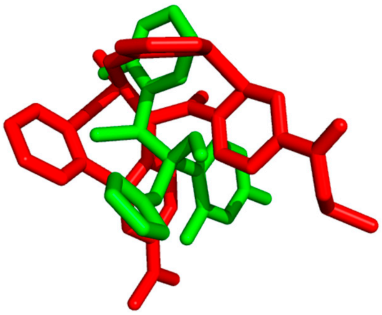

b)

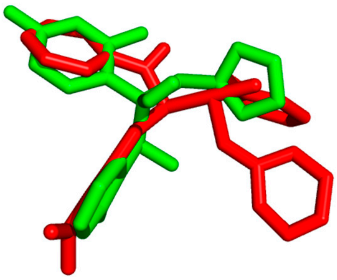

c)

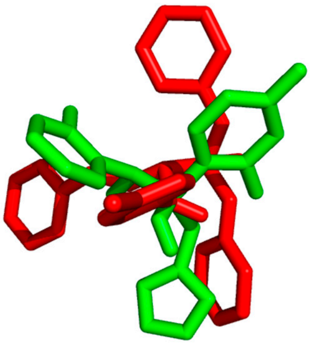

d)

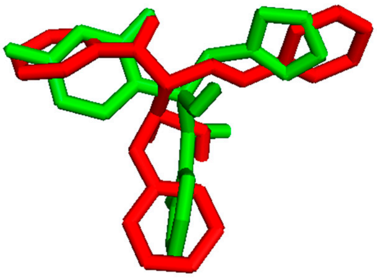

e)

Figure S1. Comparison of 3D Structural similarities of Voriconazole and active compounds, **a)** Voriconazole (green) vs **27** (red), **b)** Voriconazole (green) vs **7** (red), **c)** Voriconazole (green) vs **11** (red), **d)** Voriconazole (green) vs **13** (red), **e)** Voriconazole (green) vs **22** (red)

*All figures were visualized with Pymol (Schrödinger, San Diego, CA, USA; http://www.pymol.org/)

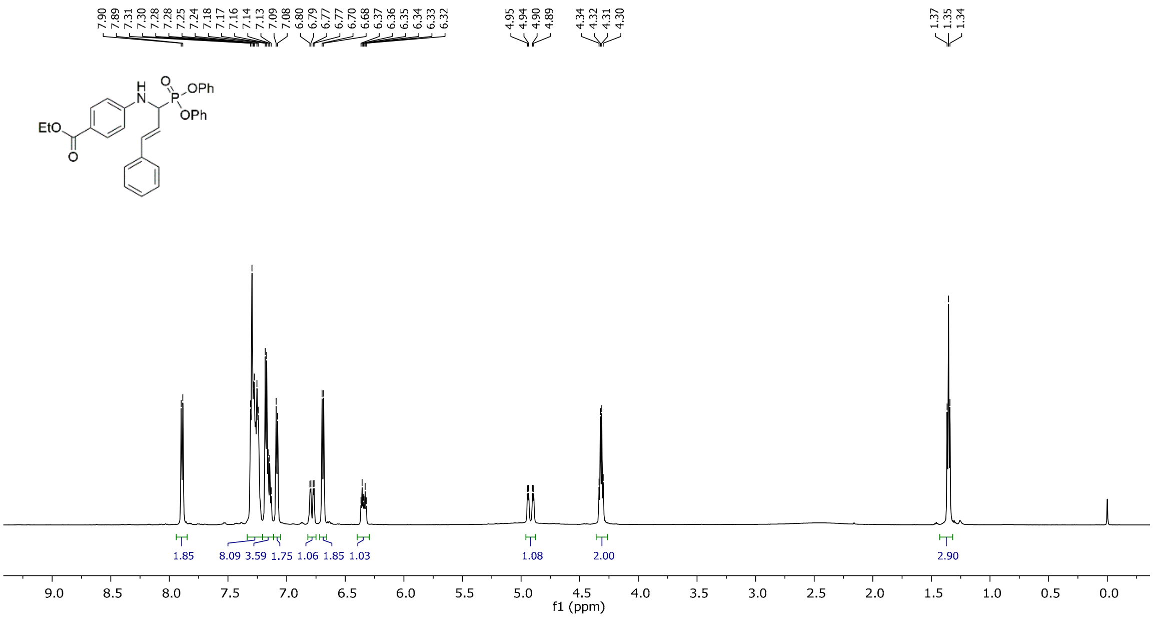

Figure S2. ^{1}H NMR (700 MHz, $CDCl_3$) of (*E*)-ethyl 4-((1-(diphenoxyphosphoryl)-3-phenylallyl)amino)benzoate (**5**)

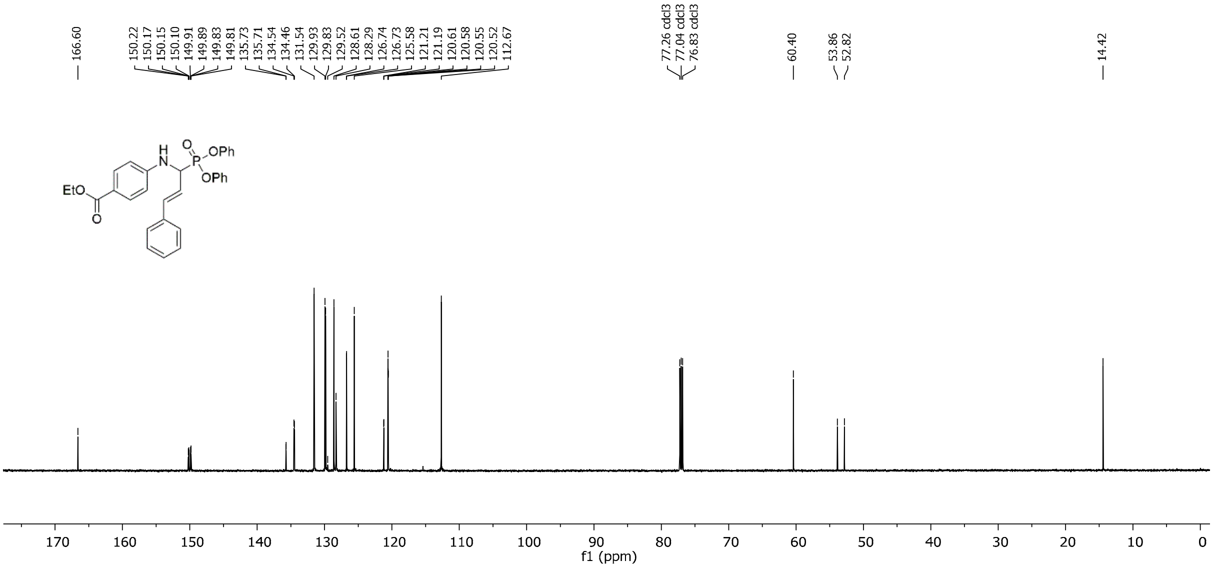

Figure S3. ^{13}C NMR (176 MHz, $CDCl_3$) of (*E*)-ethyl 4-((1-(diphenoxyphosphoryl)-3-phenylallyl)amino)benzoate (**5**)

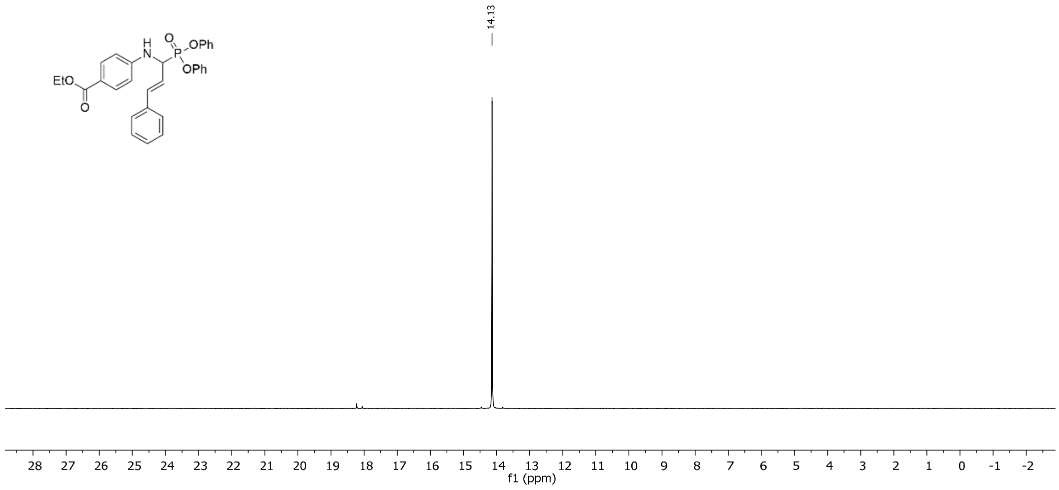

Figure S4. ^{31}P NMR (243 MHz $CDCl_3$) of (*E*)-ethyl 4-((1-(diphenoxyphosphoryl)-3-phenylallyl)amino)benzoate (**5**)

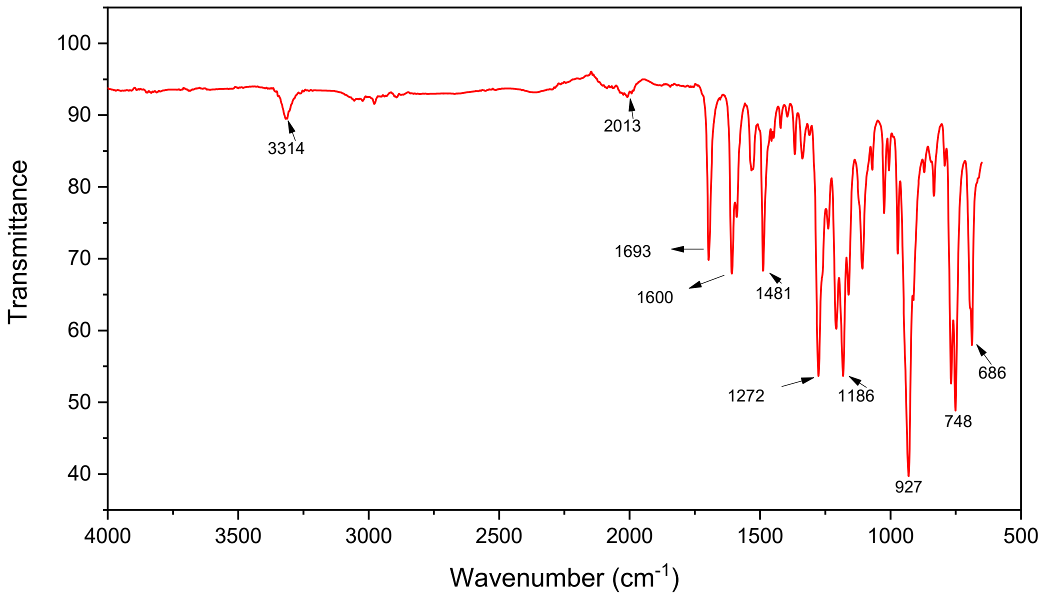

Figure S5. IR spectrum of (*E*)-ethyl 4-((1-(diphenoxyphosphoryl)-3-phenylallyl)amino)benzoate (**5**)

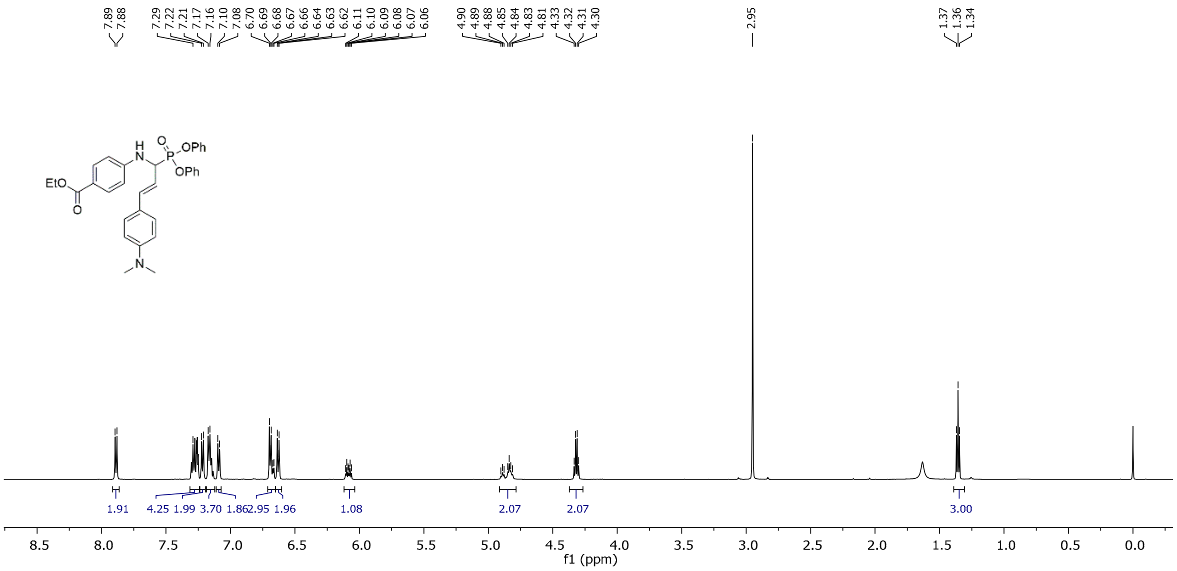

Figure S6. ^{1}H NMR (600 MHz, $CDCl_3$) of (*E*)-ethyl 4-((3-(4-(dimethylamino)phenyl)-1-(diphenoxyphosphoryl)allyl)amino)benzoate (**6**)

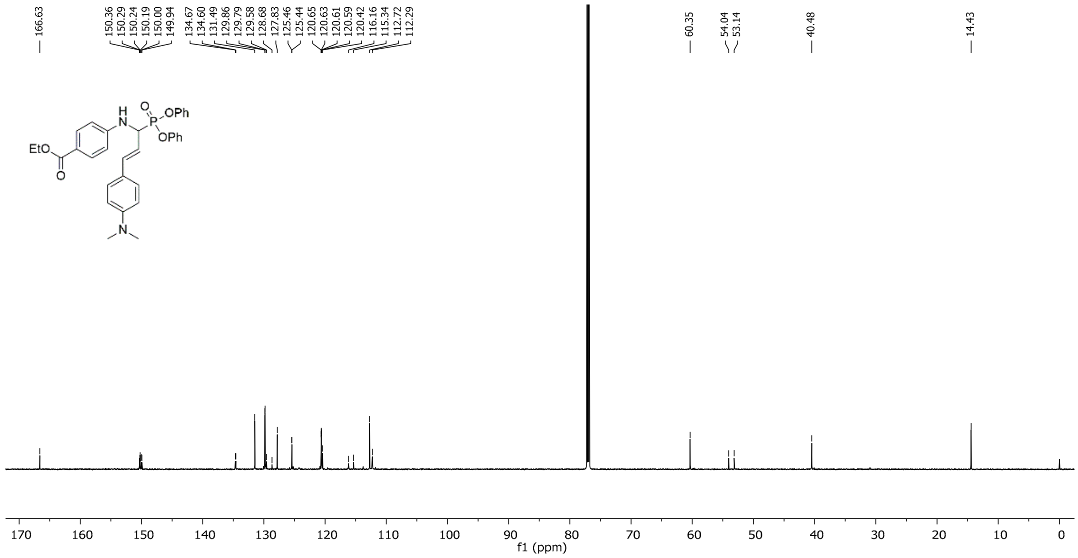

Figure S7. ^{13}C NMR (176 MHz, $CDCl_3$) of (*E*)-ethyl 4-((3-(4-(dimethylamino)phenyl)-1-(diphenoxyphosphoryl)allyl)amino)benzoate (**6**)

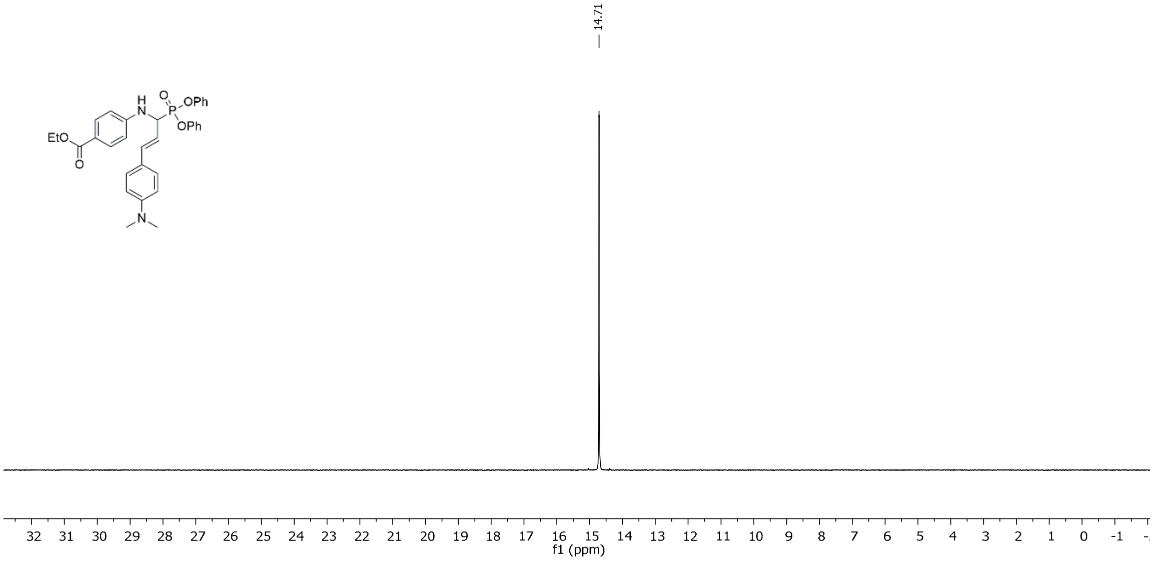

Figure S8. ^{31}P NMR (243 MHz, $CDCl_3$) of (*E*)-ethyl 4-((3-(4-(dimethylamino)phenyl)-1-(diphenoxyphosphoryl)allyl)amino)benzoate (**6**)

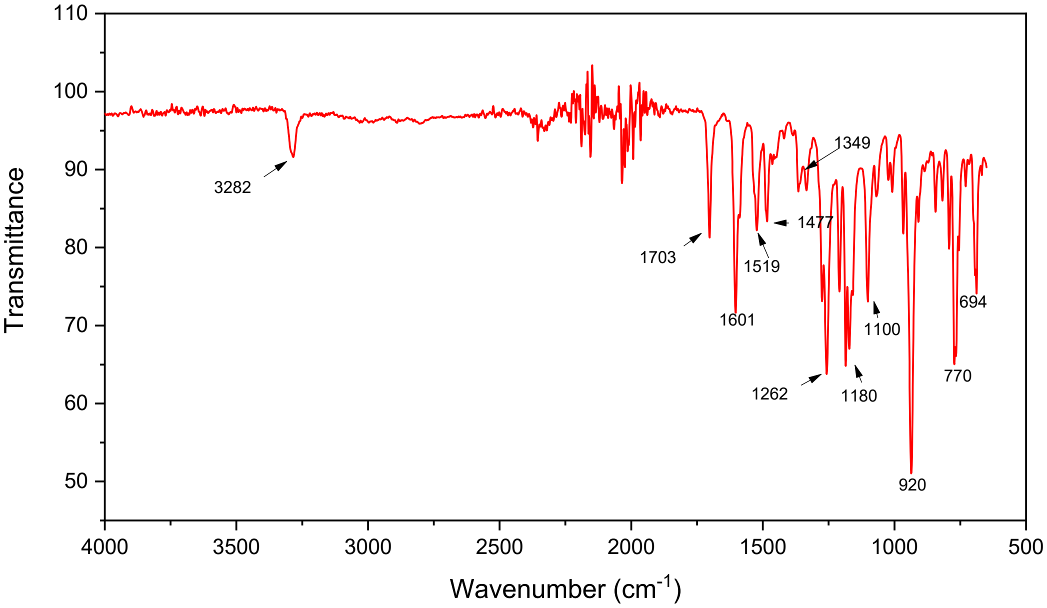

Figure S9. IR spectrum of (*E*)-ethyl 4-((3-(4-(dimethylamino)phenyl)-1-(diphenoxyphosphoryl)allyl)amino)benzoate (**6**)

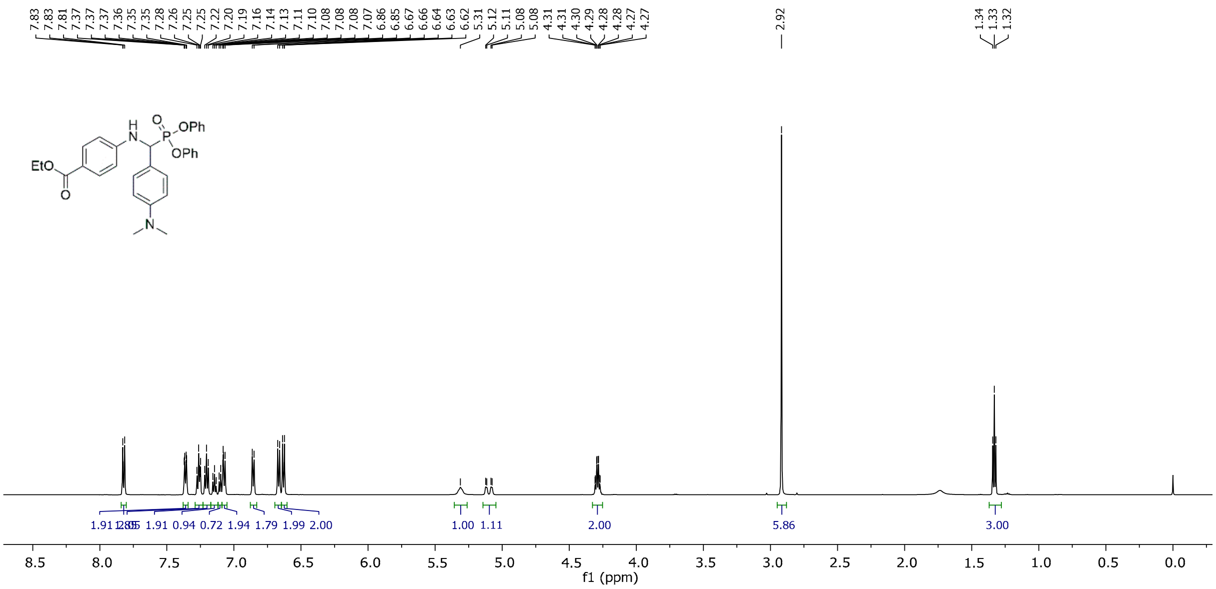

Figure S10. ^{1}H NMR (600 MHz, $CDCl_3$) of ethyl 4-(((4-(dimethylamino)phenyl)(diphenoxyphosphoryl)methyl)amino)benzoate **(7)**

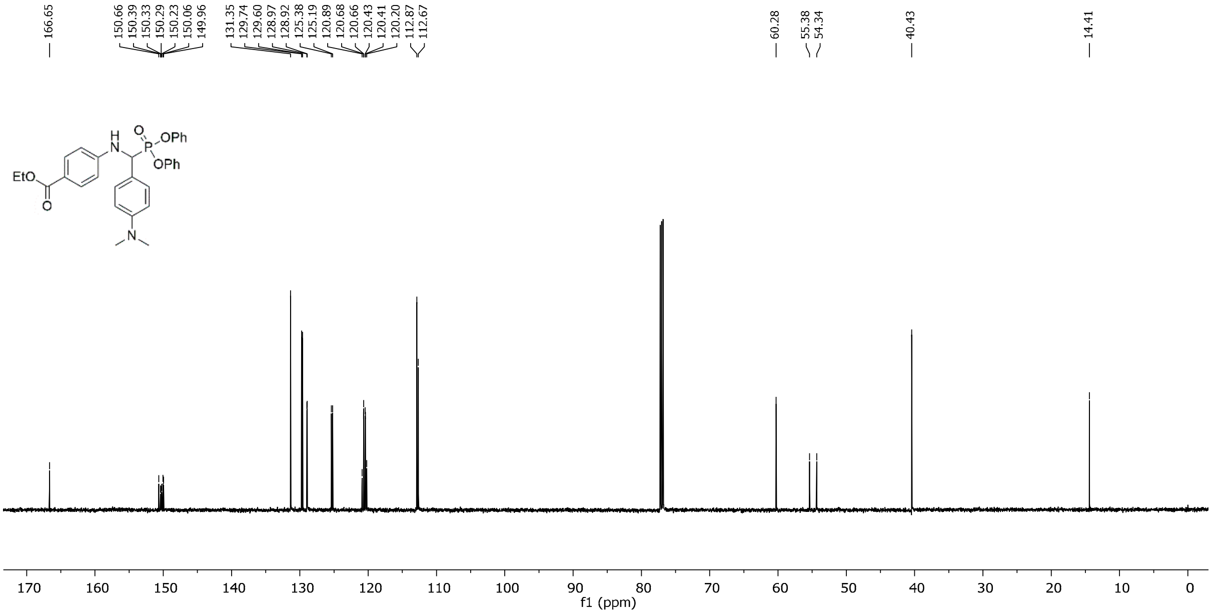

Figure S11. ^{13}C NMR (151 MHz, $CDCl_3$) of ethyl 4-(((4-(dimethylamino)phenyl)(diphenoxyphosphoryl)methyl)amino)benzoate **(7)**

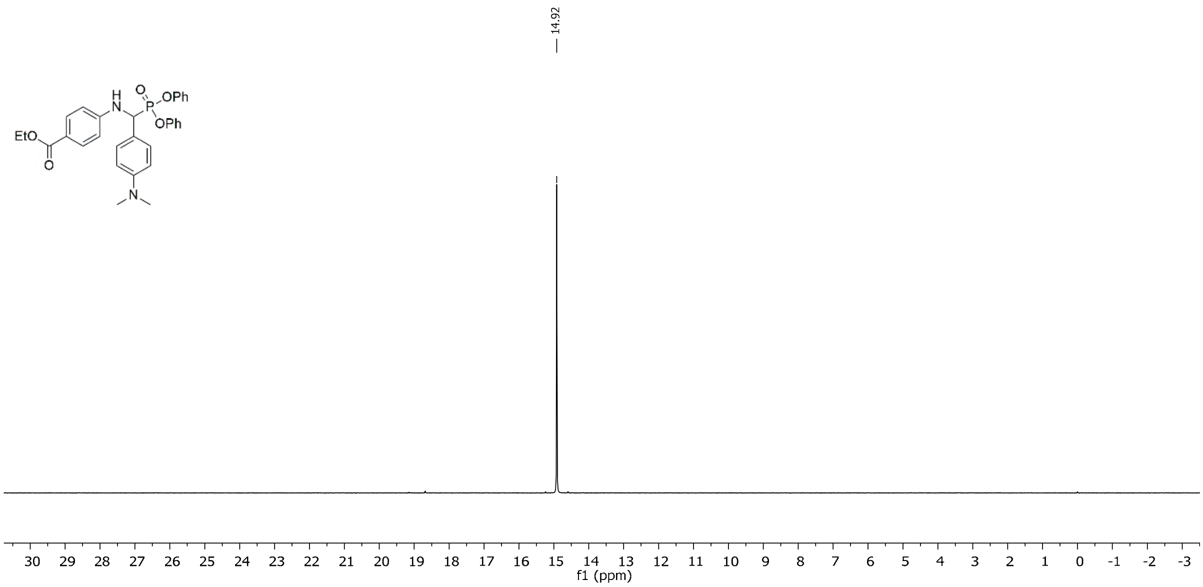

Figure S12. ^{31}P NMR (243 MHz, $CDCl_3$) of ethyl 4-(((4-(dimethylamino)phenyl)(diphenoxyphosphoryl)methyl)amino)benzoate **(7)**

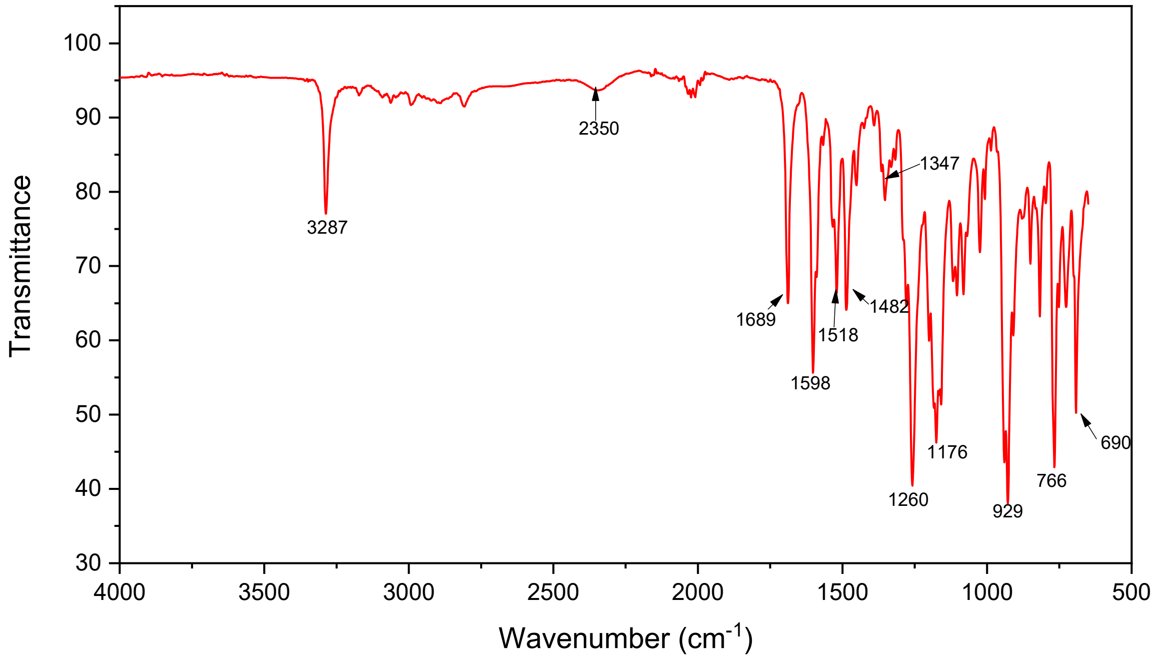

Figure S13. IR spectrum of ethyl 4-(((4-(dimethylamino)phenyl)(diphenoxyphosphoryl)methyl)amino)benzoate **(7)**

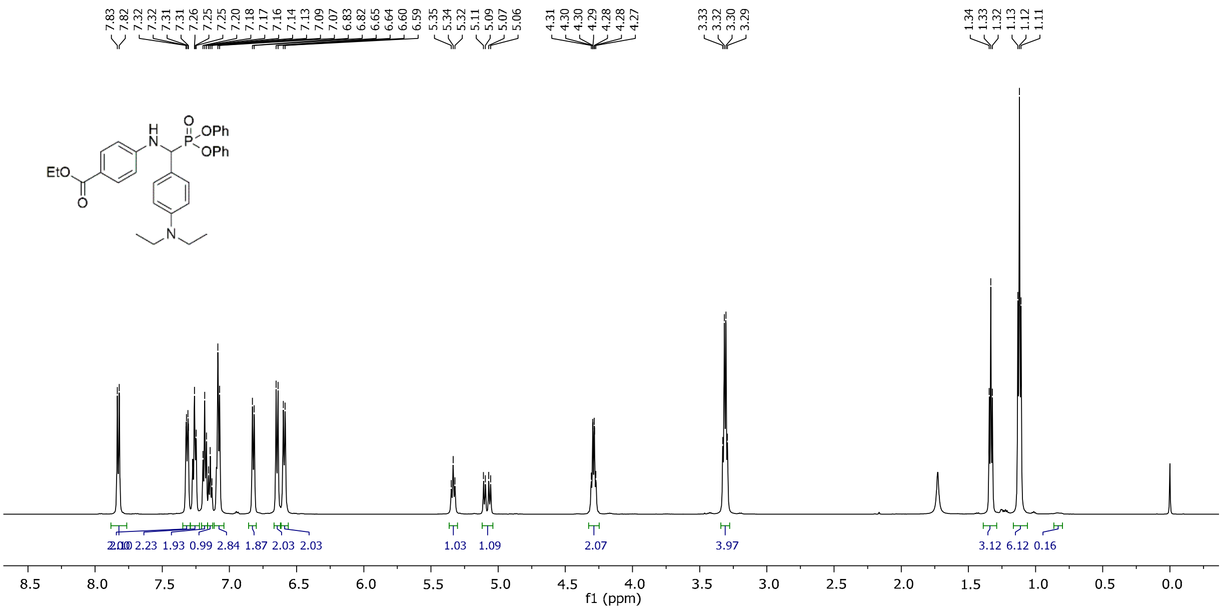

Figure S14. ^{1}H NMR (600 MHz, $CDCl_3$) of ethyl 4-(((4-(diethylamino)phenyl)(diphenoxyphosphoryl)methyl)amino)benzoate (**8**)

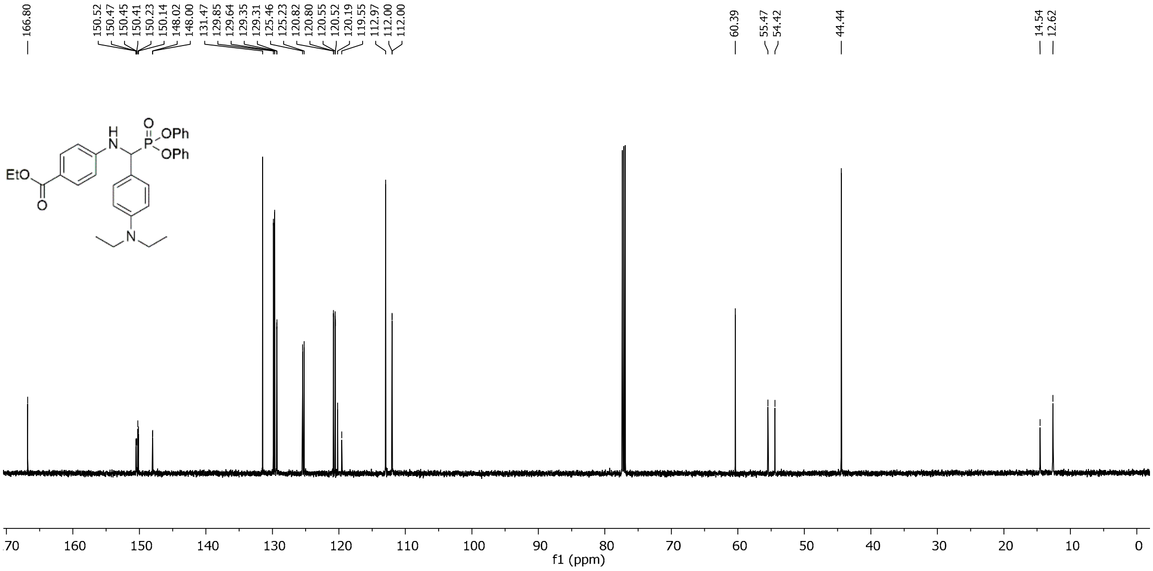

Figure S15. ^{13}C NMR (151 MHz, $CDCl_3$) of ethyl 4-(((4-(diethylamino)phenyl)(diphenoxyphosphoryl)methyl)amino)benzoate (**8**)

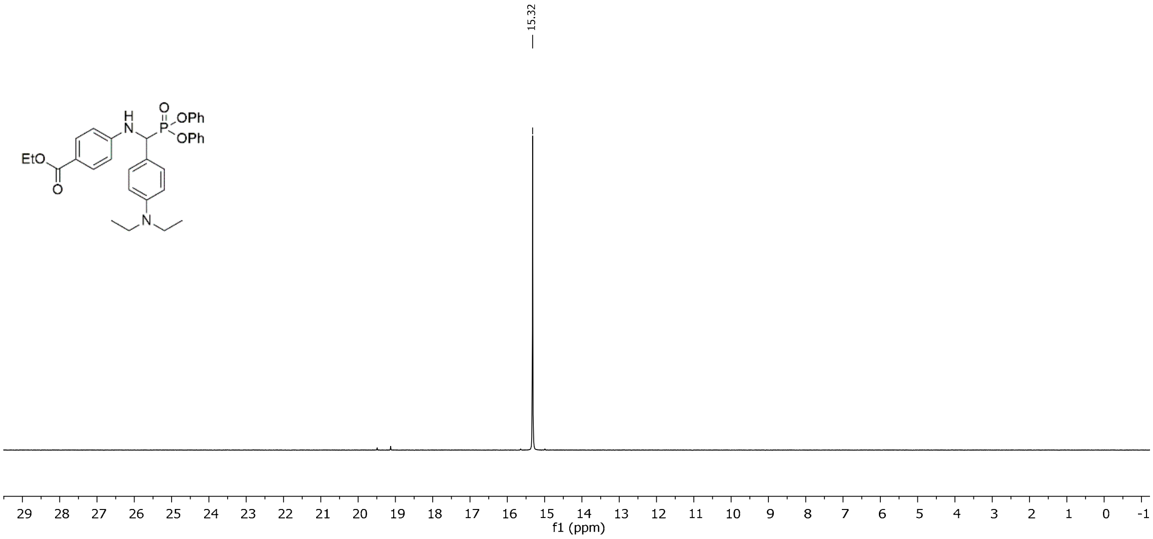

Figure S16. ^{31}P NMR (243 MHz, $CDCl_3$) of ethyl 4-(((4-(diethylamino)phenyl)(diphenoxyphosphoryl)methyl)amino)benzoate (**8**)

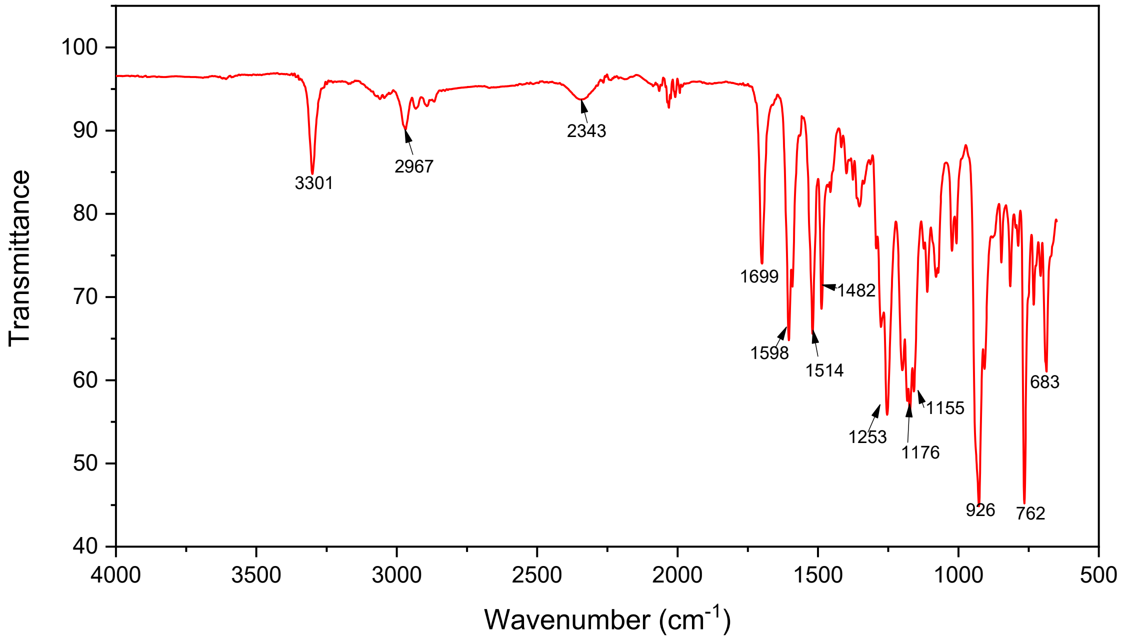

Figure S17. IR spectrum of ethyl 4-(((4-(diethylamino)phenyl)(diphenoxyphosphoryl)methyl)amino)benzoate (**8**)

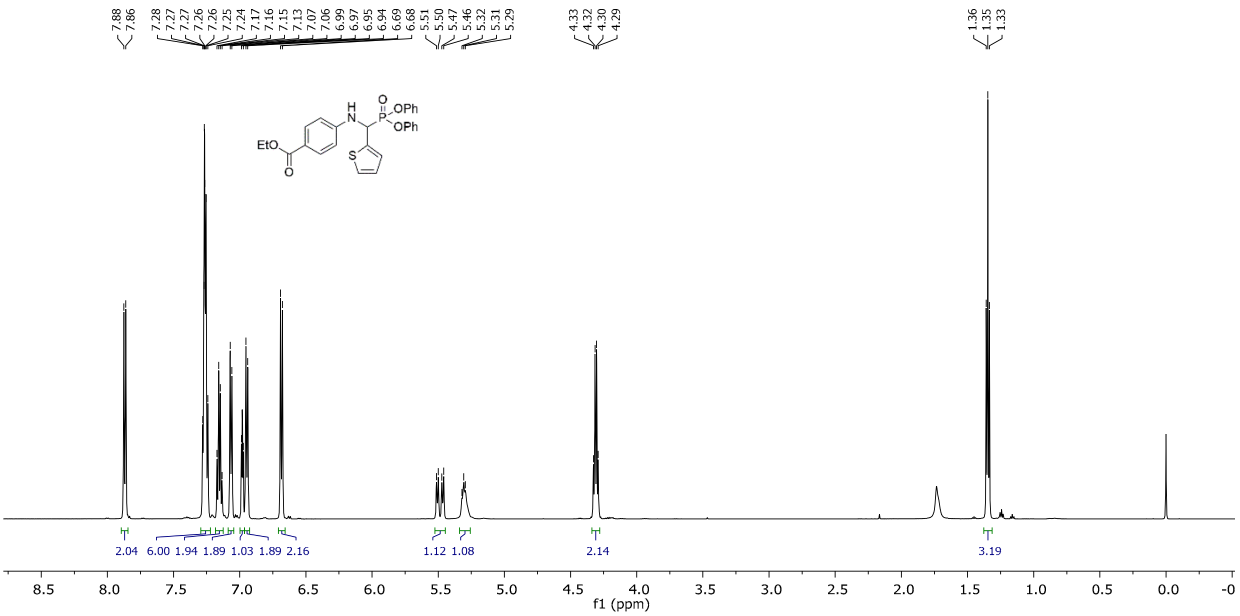

Figure S18. ^{1}H NMR (600 MHz, $CDCl_3$) of ethyl 4-(((diphenoxyphosphoryl)(thiophen-2-yl)methyl)amino)benzoate (**9**)

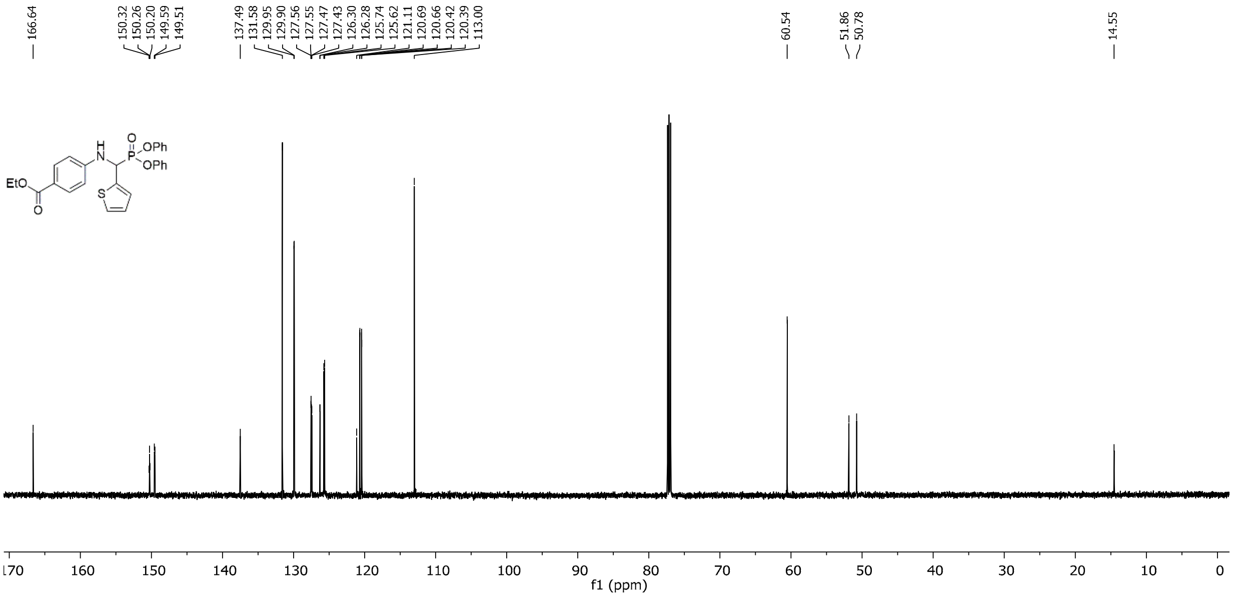

Figure S19. ^{13}C NMR (151 MHz, $CDCl_3$) of ethyl 4-(((diphenoxyphosphoryl)(thiophen-2-yl)methyl)amino)benzoate (**9**)

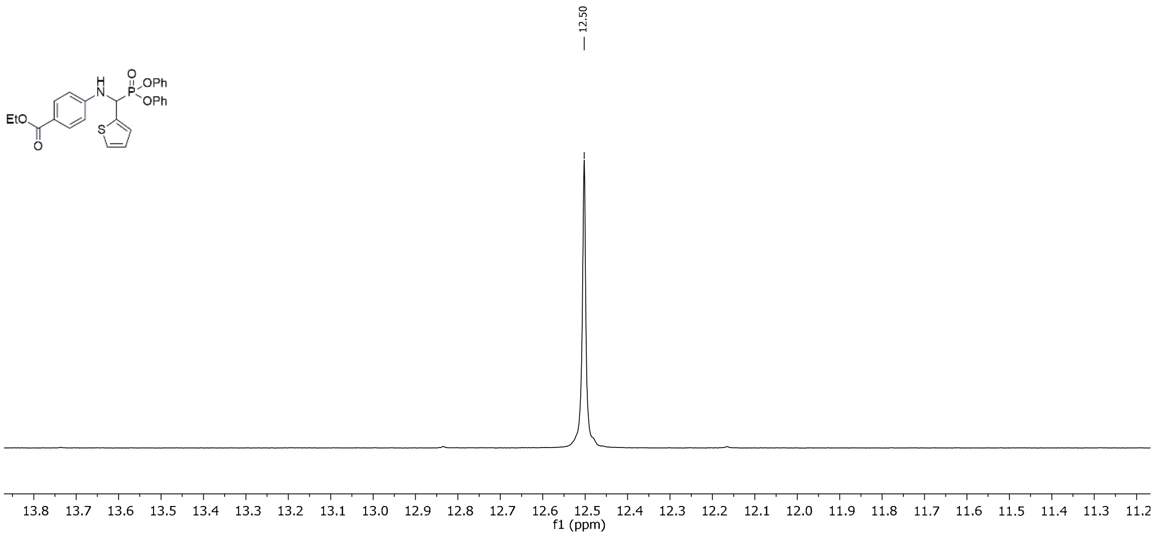

Figure S20. ^{31}P NMR (243 MHz, $CDCl_3$) of ethyl 4-(((diphenoxyphosphoryl)(thiophen-2-yl)methyl)amino)benzoate (**9**)

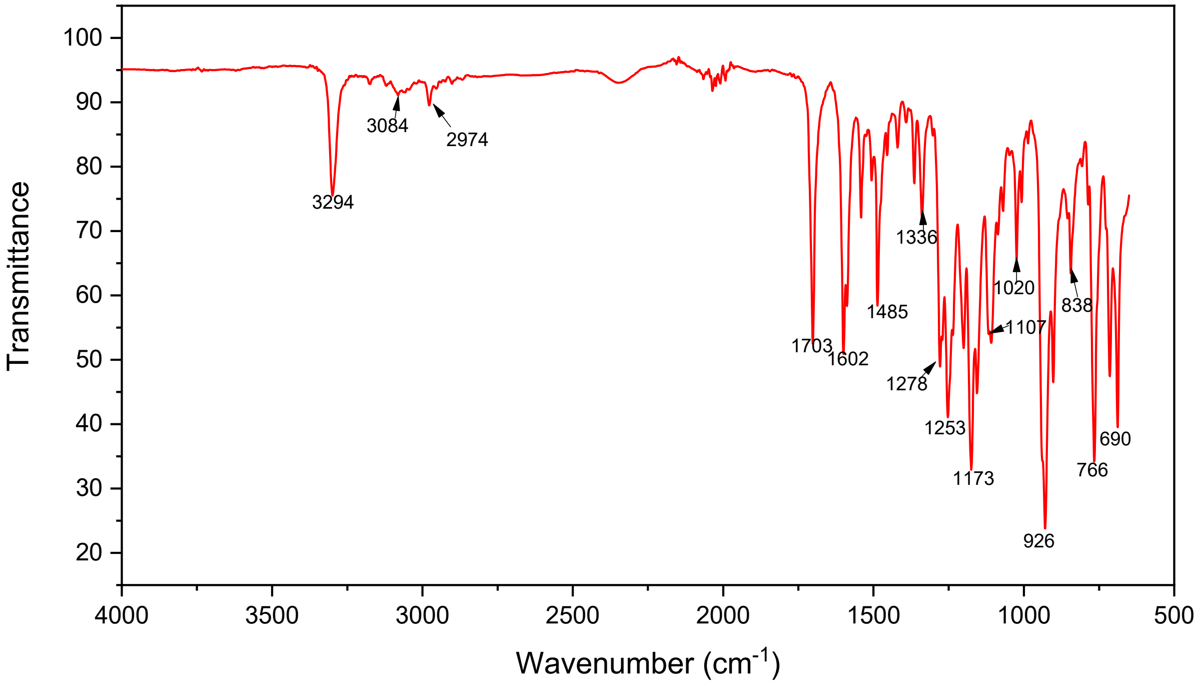

Figure S21. IR spectrum of ethyl 4-(((diphenoxyphosphoryl)(thiophen-2-yl)methyl)amino)benzoate (**9**)

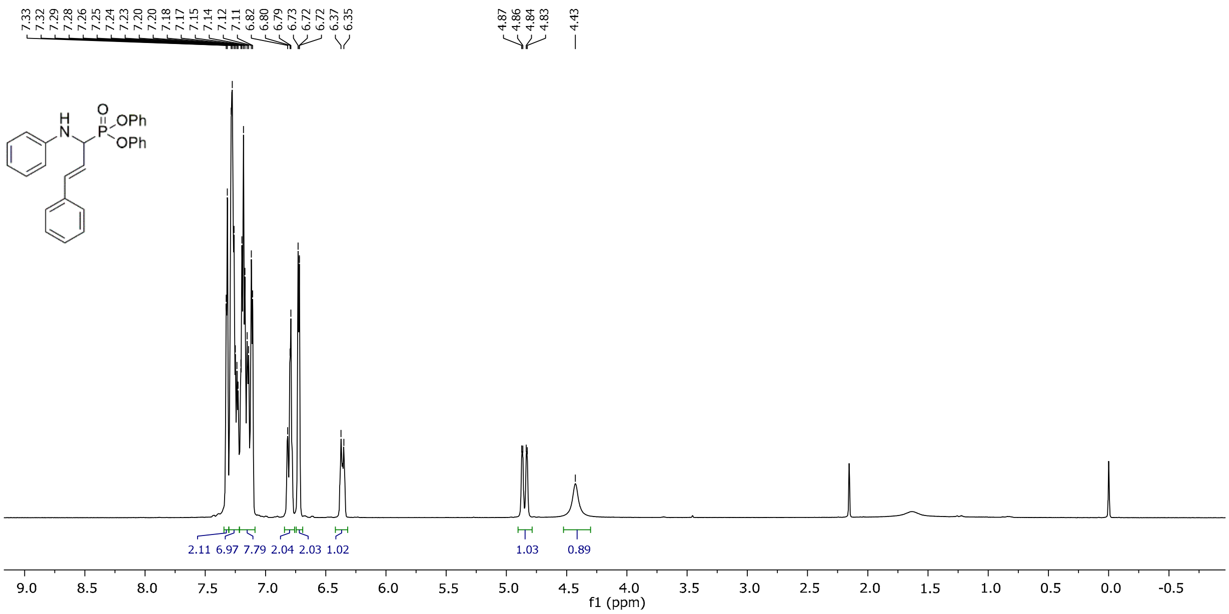

Figure S22. ^{1}H NMR (700 MHz, $CDCl_3$) of (*E*)-diphenyl (3-phenyl-1-(phenylamino)allyl)phosphonate (**10**)

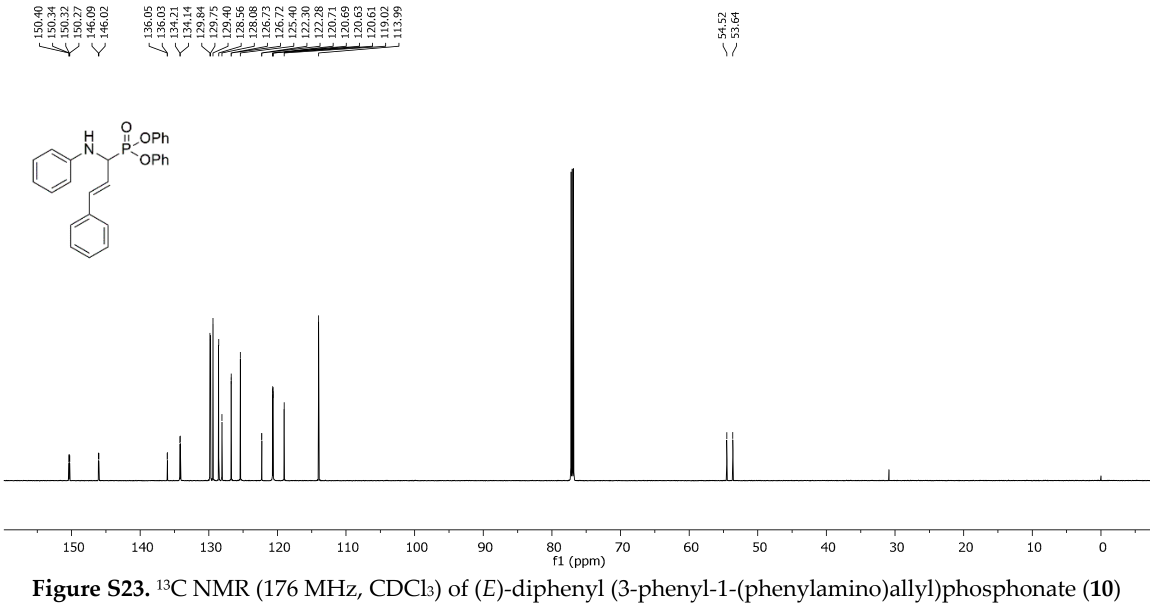

Figure S23. ^{13}C NMR (176 MHz, $CDCl_3$) of (*E*)-diphenyl (3-phenyl-1-(phenylamino)allyl)phosphonate (**10**)

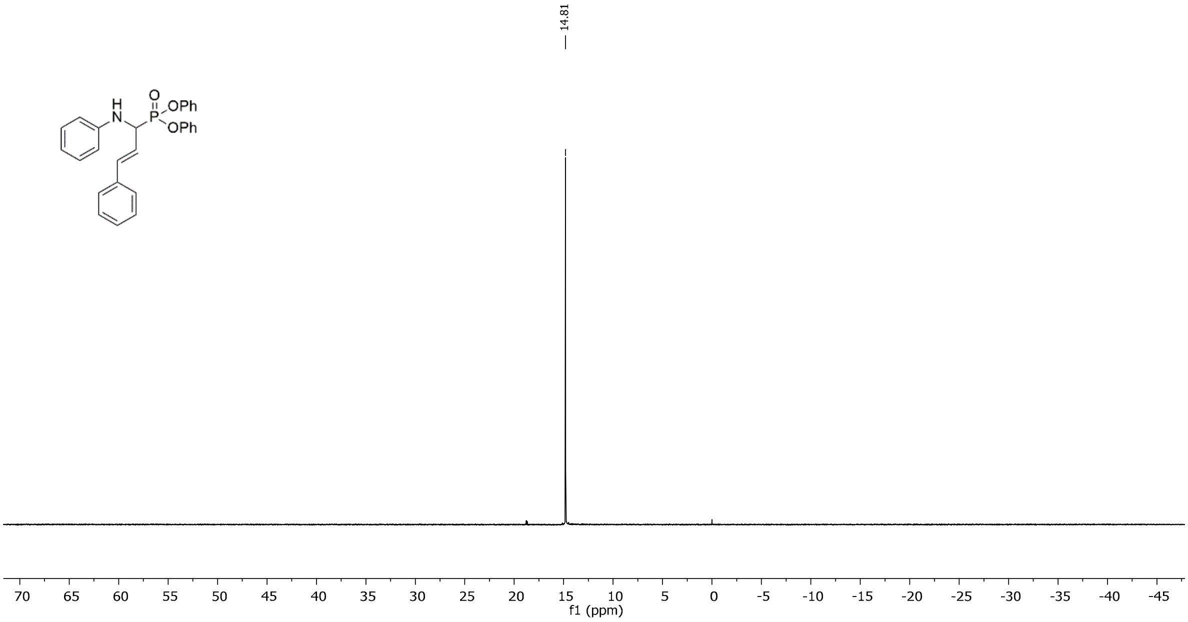

Figure S24. ^{31}P NMR (243 MHz, $CDCl_3$) of (*E*)-diphenyl (3-phenyl-1-(phenylamino)allyl)phosphonate (**10**)

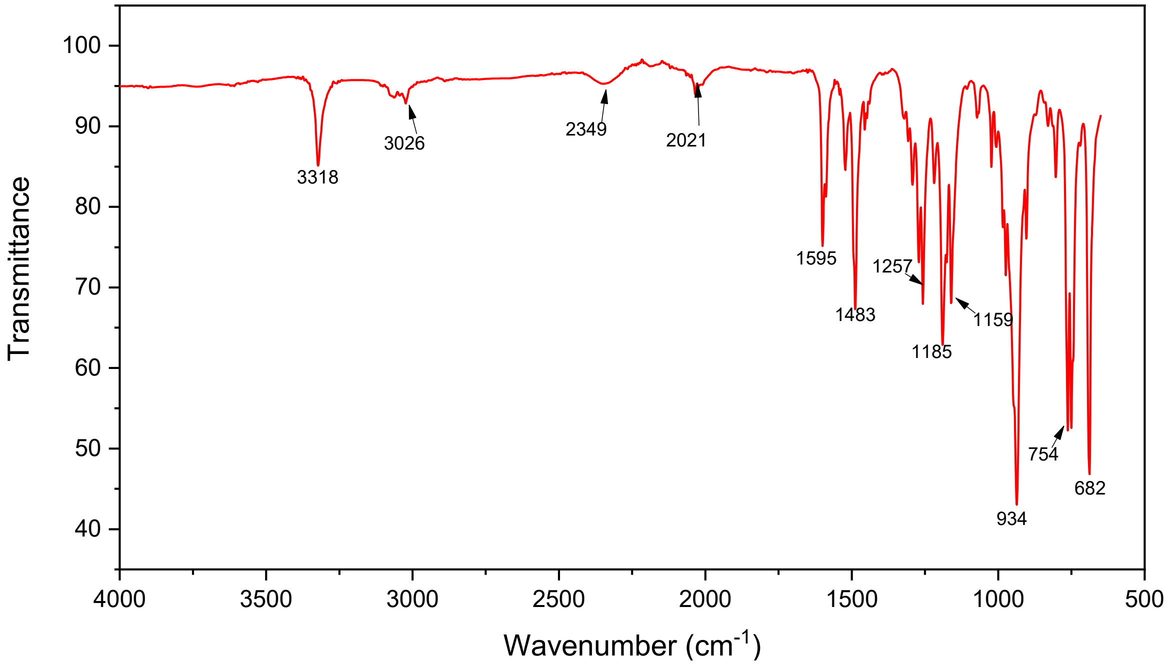

Figure S25. IR spectrum of (*E*)-diphenyl (3-phenyl-1-(phenylamino)allyl)phosphonate (**10**)

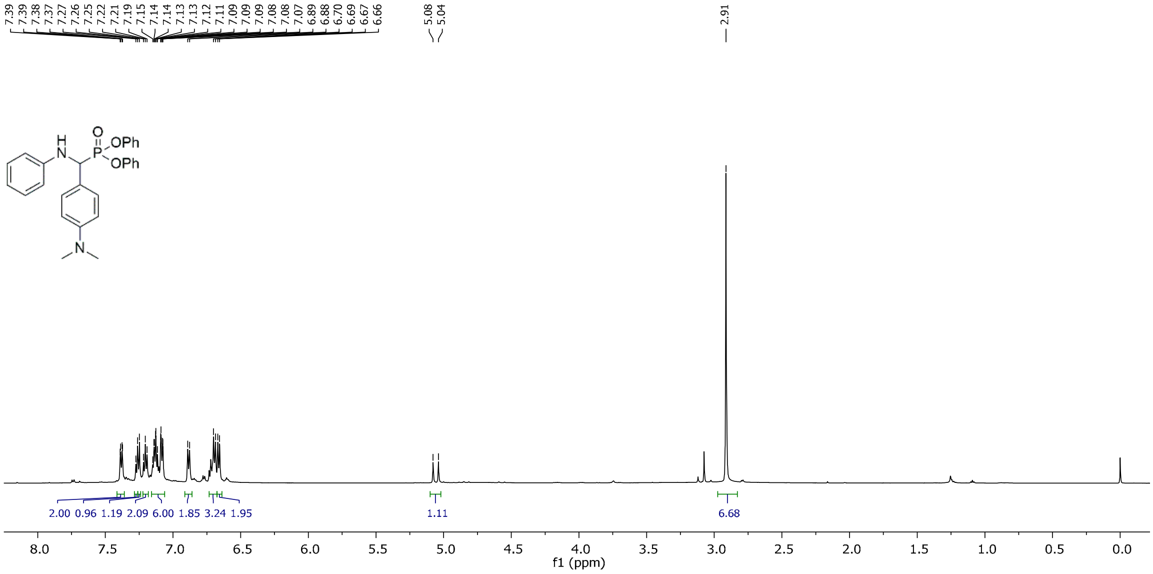

Figure S26. ^{1}H NMR (600 MHz, $CDCl_3$) of diphenyl ((4-(dimethylamino)phenyl)(phenylamino)methyl)phosphonate (**11**)

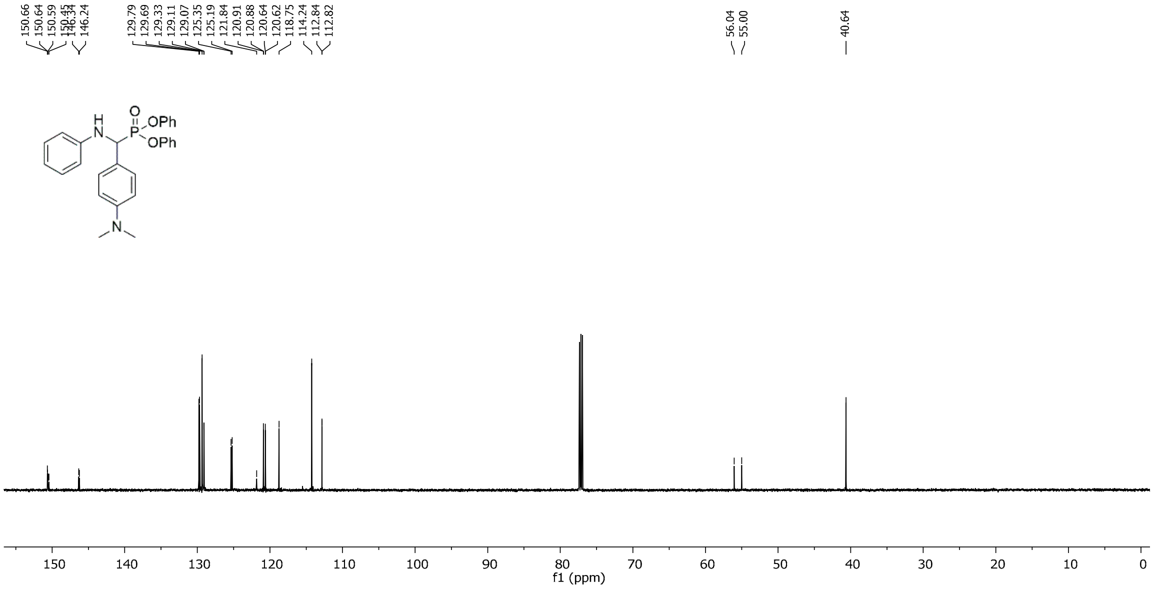

Figure S27. ^{13}C NMR (151 MHz, $CDCl_3$) of diphenyl ((4-(dimethylamino)phenyl)(phenylamino)methyl)phosphonate (**11**)

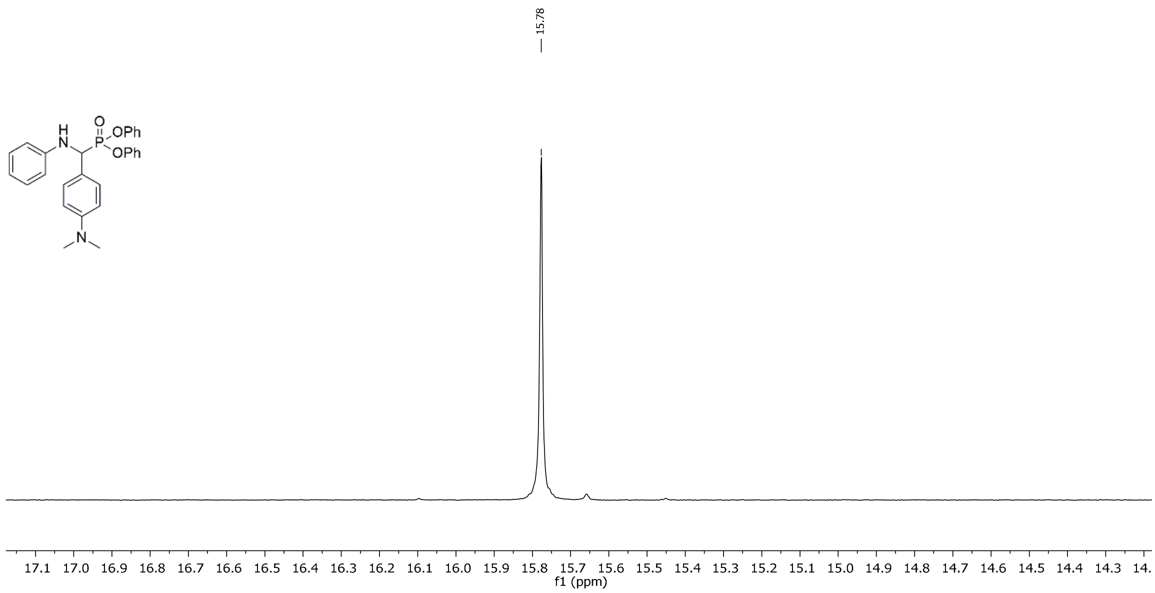

Figure S28. ^{31}P NMR (243 MHz, $CDCl_3$) of diphenyl ((4-(dimethylamino)phenyl)(phenylamino)methyl)phosphonate (**11**)

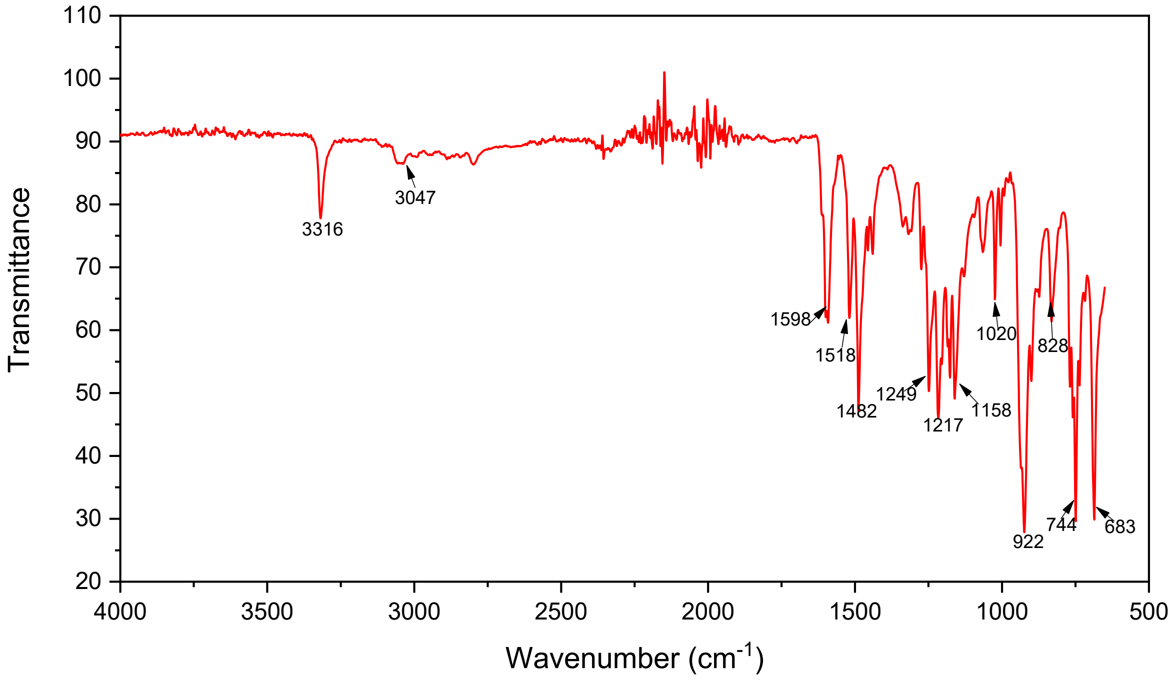

Figure S29. IR spectrum of diphenyl ((4-(dimethylamino)phenyl)(phenylamino)methyl)phosphonate (**11**)

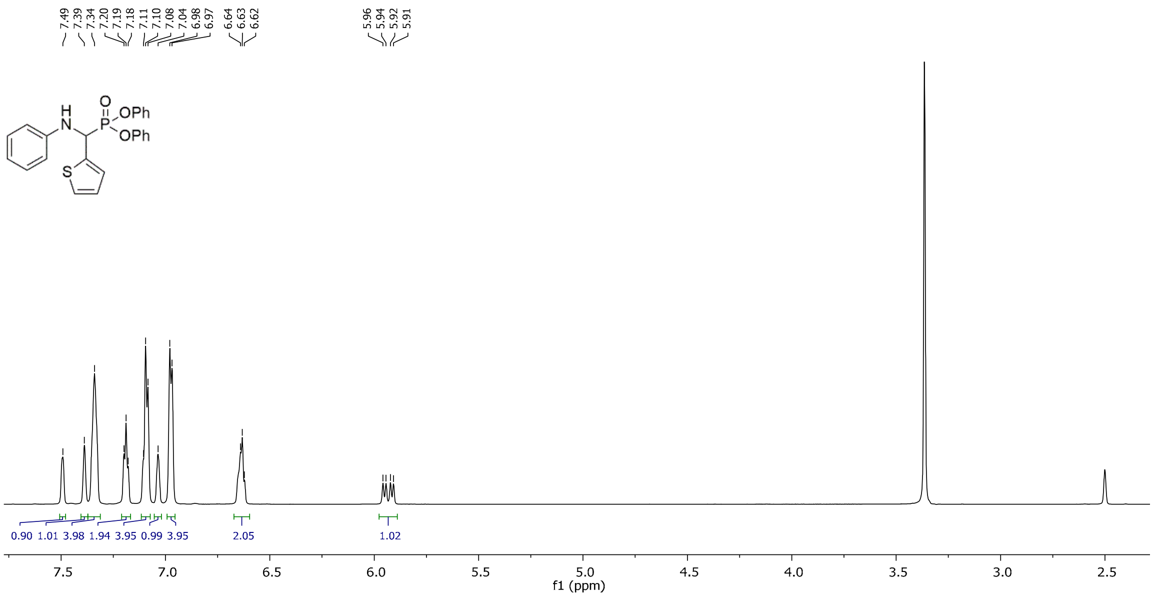

Figure S30. ^{1}H NMR (700 MHz, DMSO-d_6) of diphenyl ((phenylamino)(thiophen-2-yl)methyl)phosphonate (**12**)

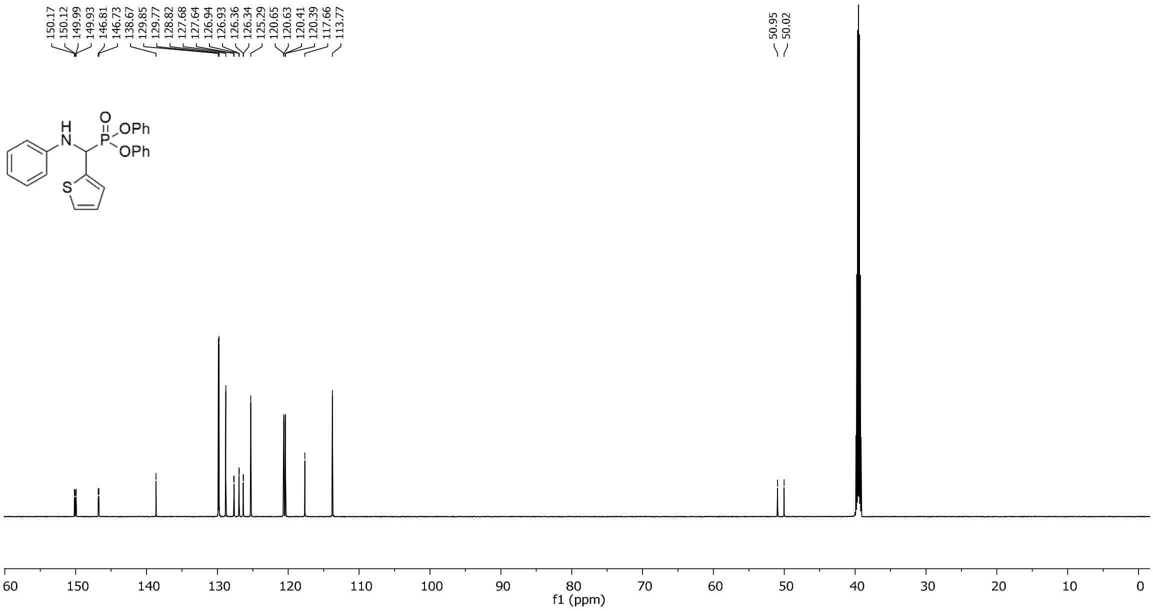

Figure S31. ^{13}C NMR (176 MHz, DMSO-d_6) of diphenyl ((phenylamino)(thiophen-2-yl)methyl)phosphonate (**12**)

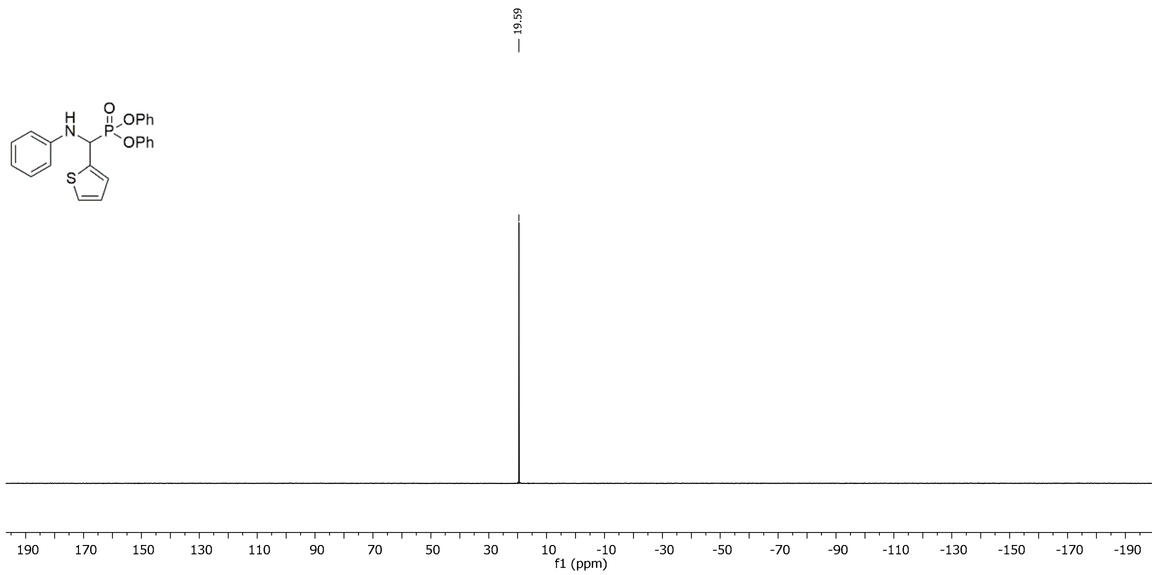

Figure S32. ^{31}P NMR (243 MHz, DMSO-d_6) of diphenyl ((phenylamino)(thiophen-2-yl)methyl)phosphonate (**12**)

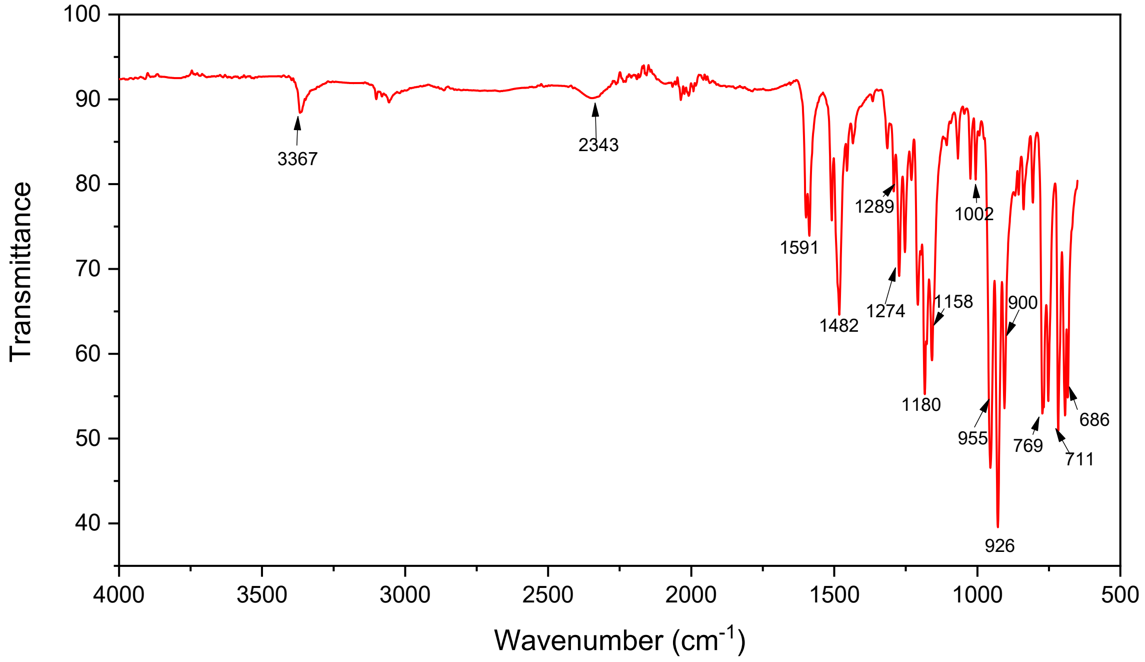

Figure S33. IR spectrum of diphenyl ((phenylamino)(thiophen-2-yl)methyl)phosphonate (**12**)

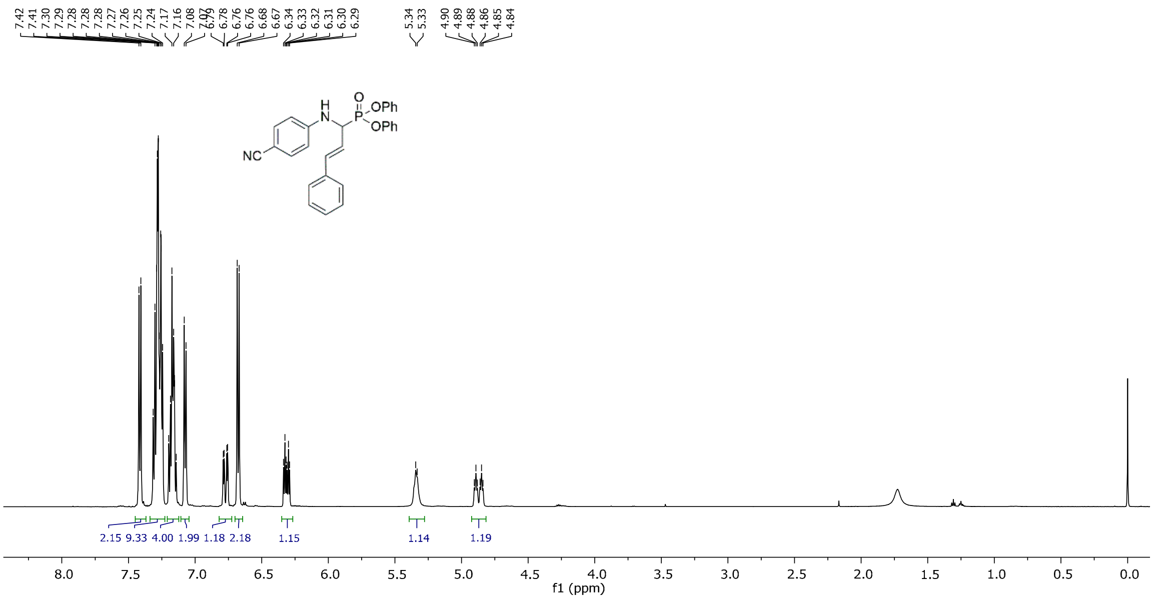

Figure S34. ^{1}H NMR (600 MHz, $CDCl_3$) of (*E*)-diphenyl (1-((4-cyanophenyl)amino)-3-phenylallyl)phosphonate (**13**)

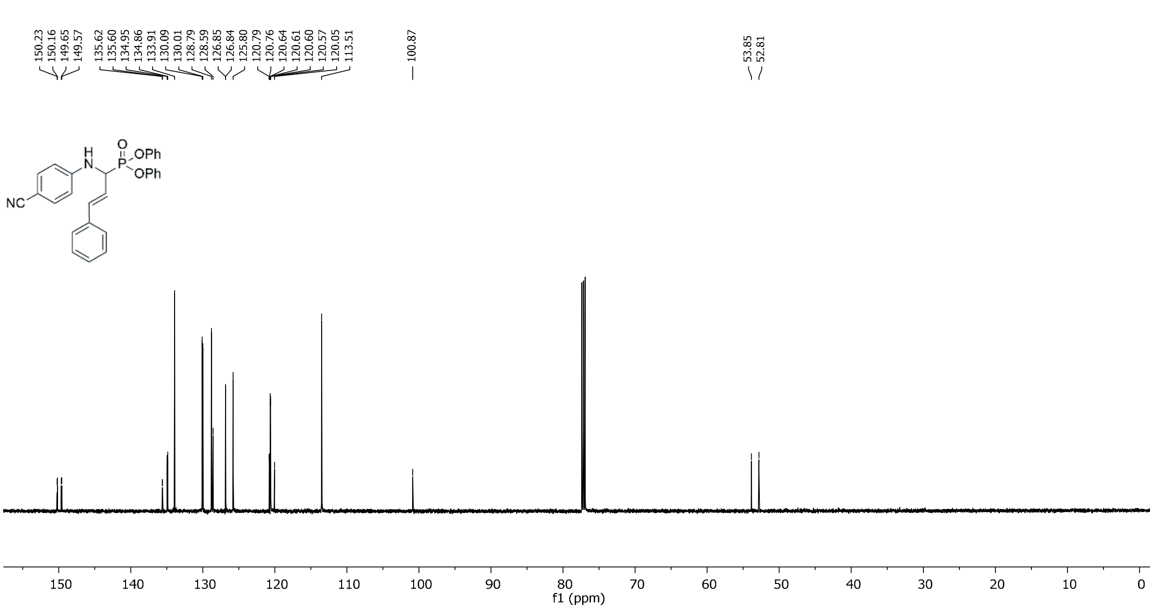

Figure S35. ^{13}C NMR (151 MHz, $CDCl_3$) of (*E*)-diphenyl (1-((4-cyanophenyl)amino)-3-phenylallyl)phosphonate (**13**)

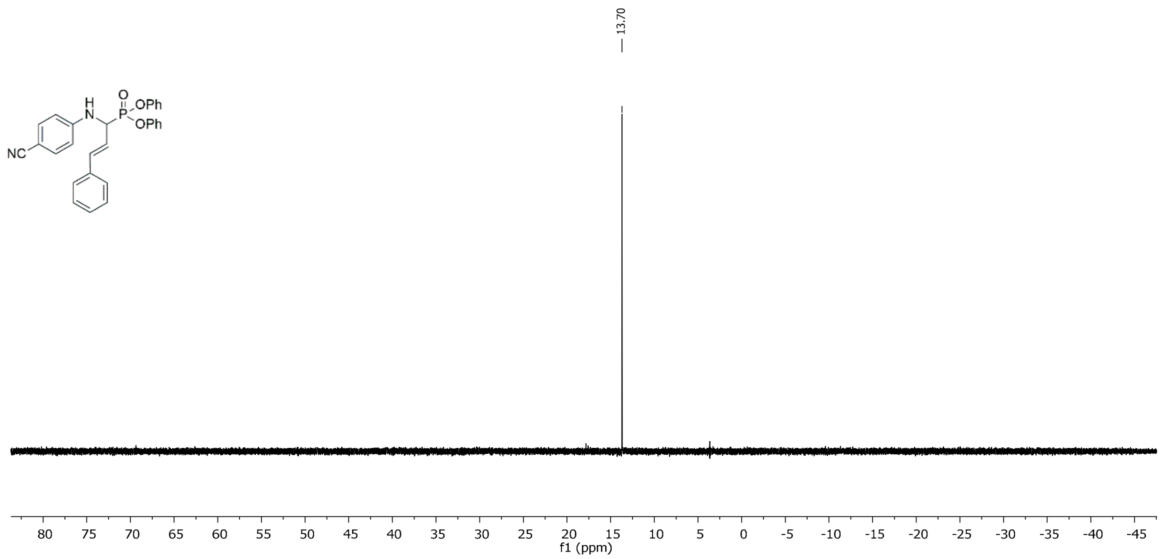

Figure S36. ^{31}P NMR (243 MHz, $CDCl_3$) of (*E*)-diphenyl (1-((4-cyanophenyl)amino)-3-phenylallyl)phosphonate (**13**)

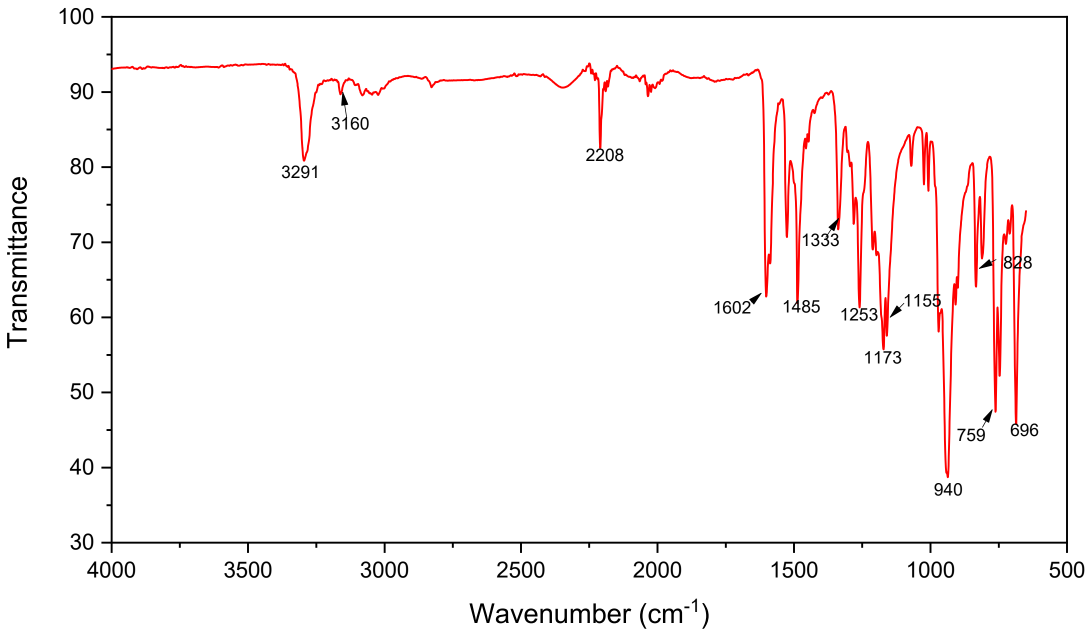

Figure S37. IR spectrum of (*E*)-diphenyl (1-((4-cyanophenyl)amino)-3-phenylallyl)phosphonate (**13**)

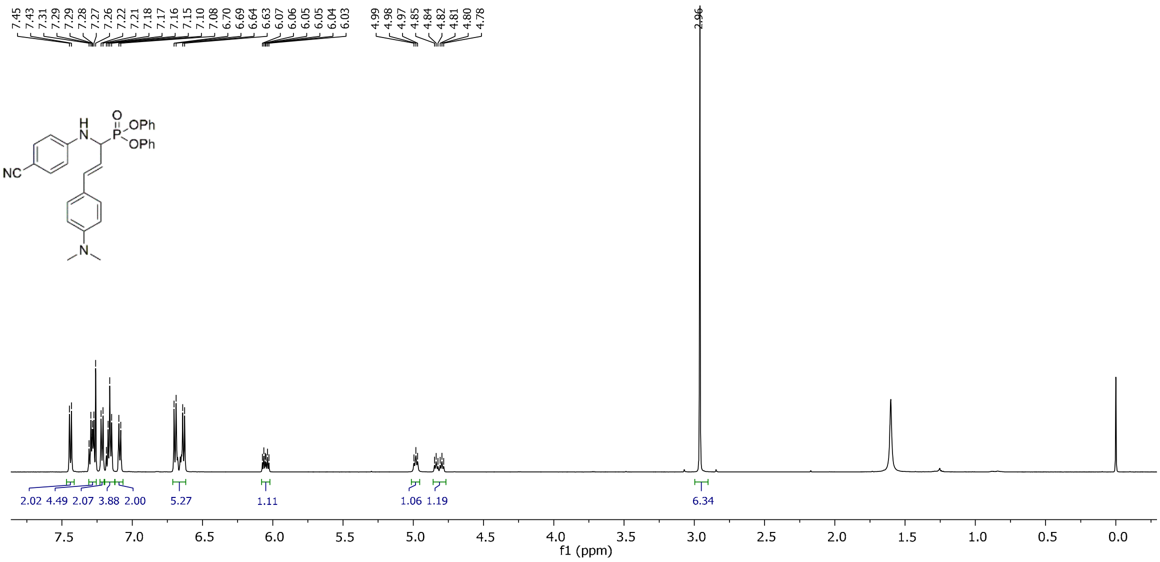

Figure S38. ^{1}H NMR (600 MHz, $CDCl_3$) of (*E*)-diphenyl (1-((4-cyanophenyl)amino)-3-(4-(dimethylamino)phenyl)allyl)phosphonate (**14**)

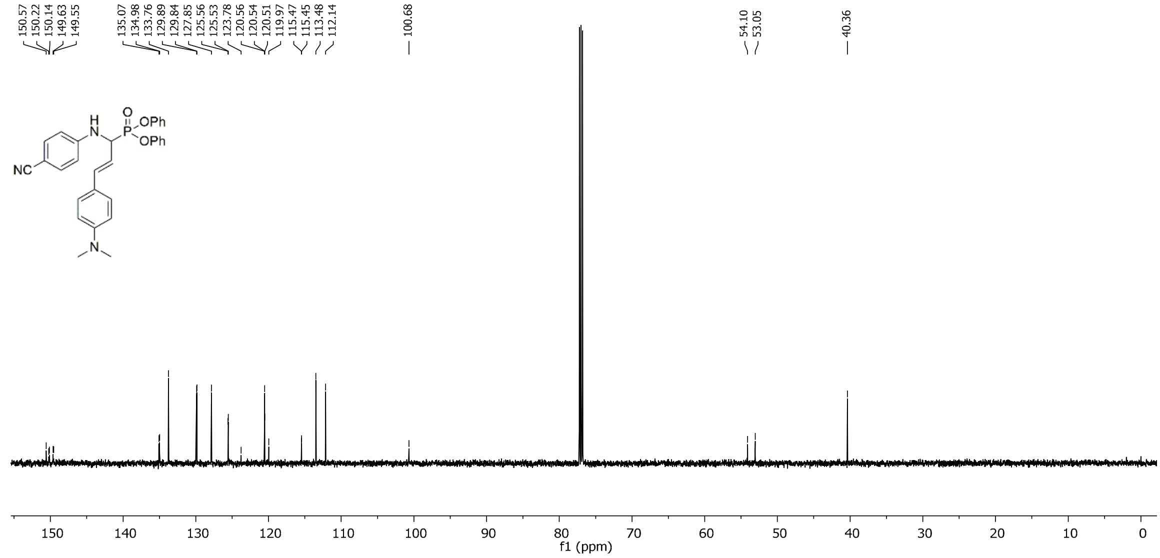

Figure S39. ^{13}C NMR (151 MHz, $CDCl_3$) of (*E*)-diphenyl (1-((4-cyanophenyl)amino)-3-(4-(dimethylamino)phenyl)allyl)phosphonate (**14**)

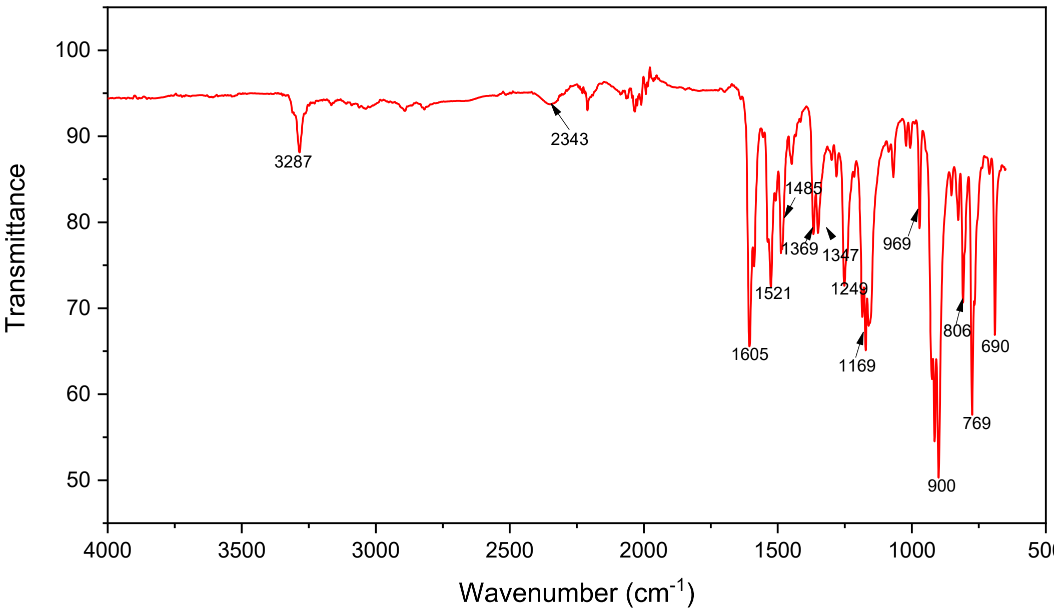

Figure S40. IR spectrum of (*E*)-diphenyl (1-((4-cyanophenyl)amino)-3-(4-(dimethylamino)phenyl)allyl)phosphonate (**14**)

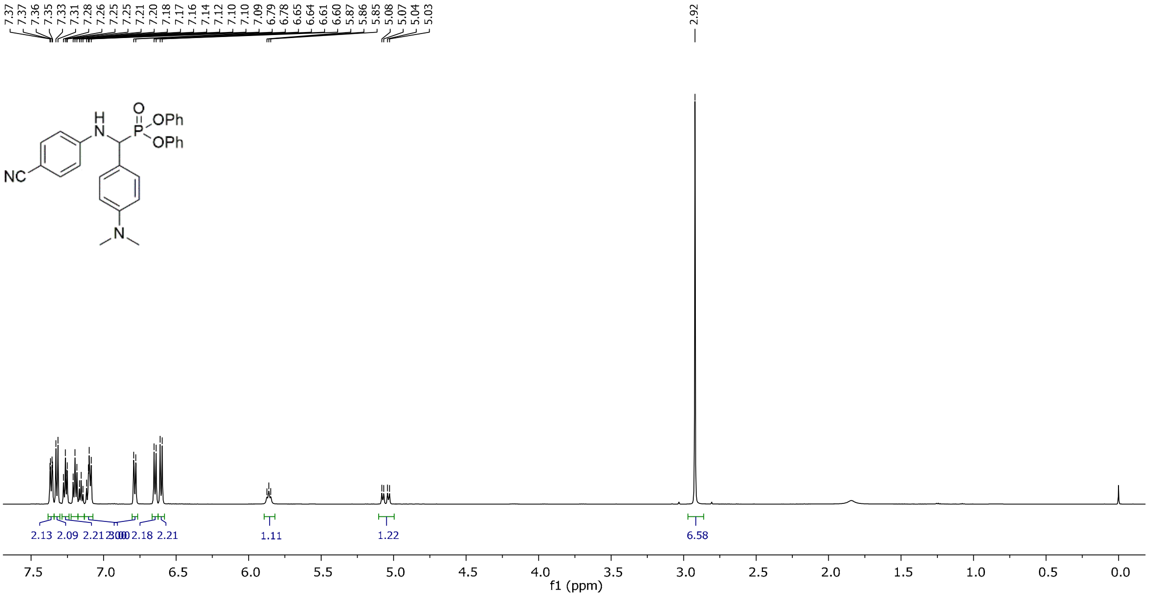

Figure S41. ^{1}H NMR (600 MHz, $CDCl_3$) of diphenyl (((4-cyanophenyl)amino)(4-(dimethylamino)phenyl)methyl)phosphonate (**15**)

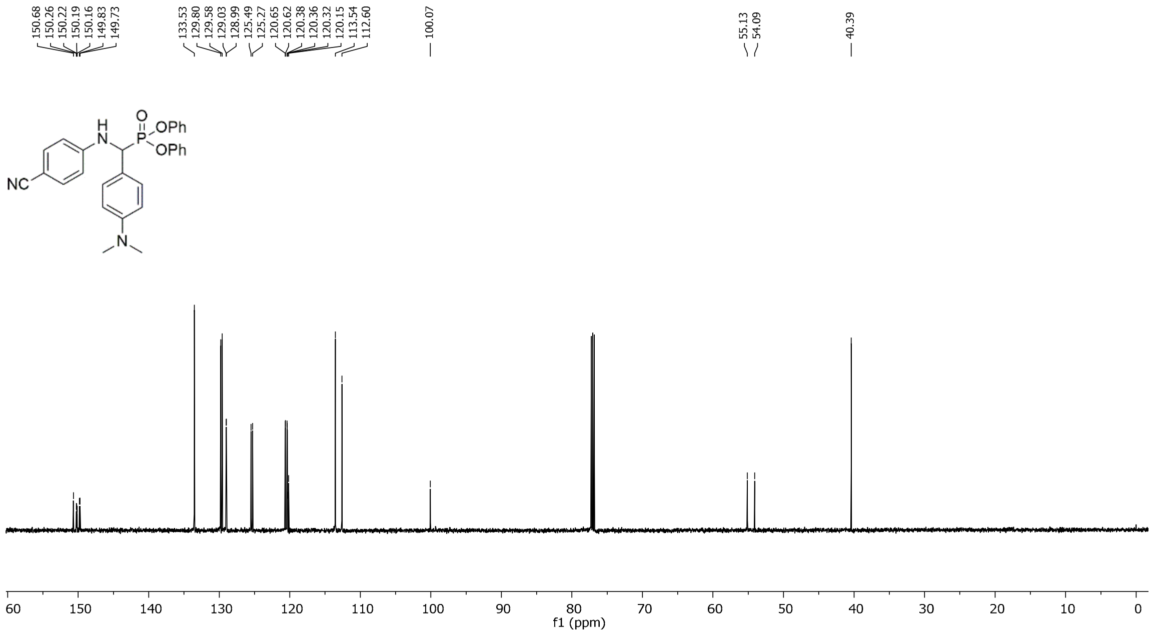

Figure S42. ^{13}C NMR (151 MHz, $CDCl_3$) of diphenyl (((4-cyanophenyl)amino)(4-(dimethylamino)phenyl)methyl)phosphonate (**15**)

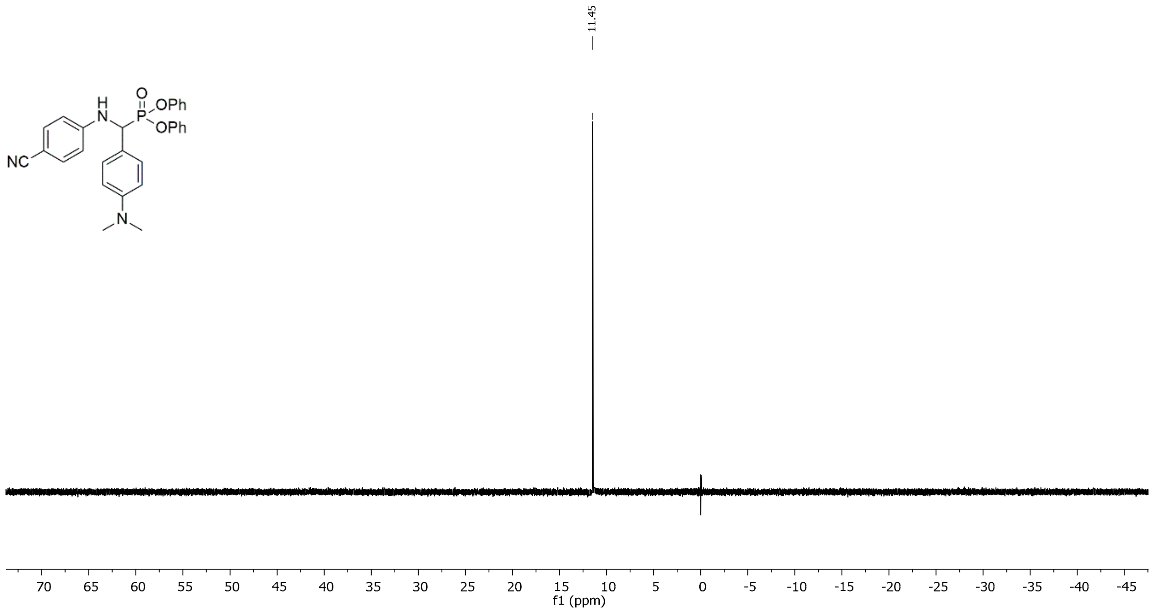

Figure S43. ^{31}P NMR (243 MHz, $CDCl_3$) of diphenyl (((4-cyanophenyl)amino)(4-(dimethylamino)phenyl)methyl)phosphonate (**15**)

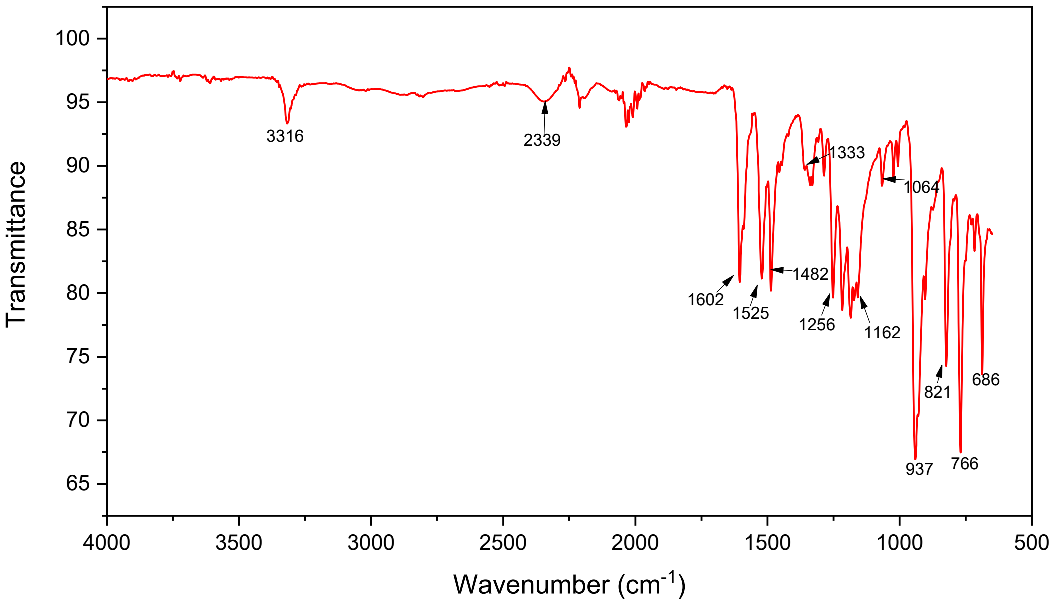

Figure S44. IR spectrum of diphenyl (((4-cyanophenyl)amino)(4-(dimethylamino)phenyl)methyl)phosphonate (**15**)

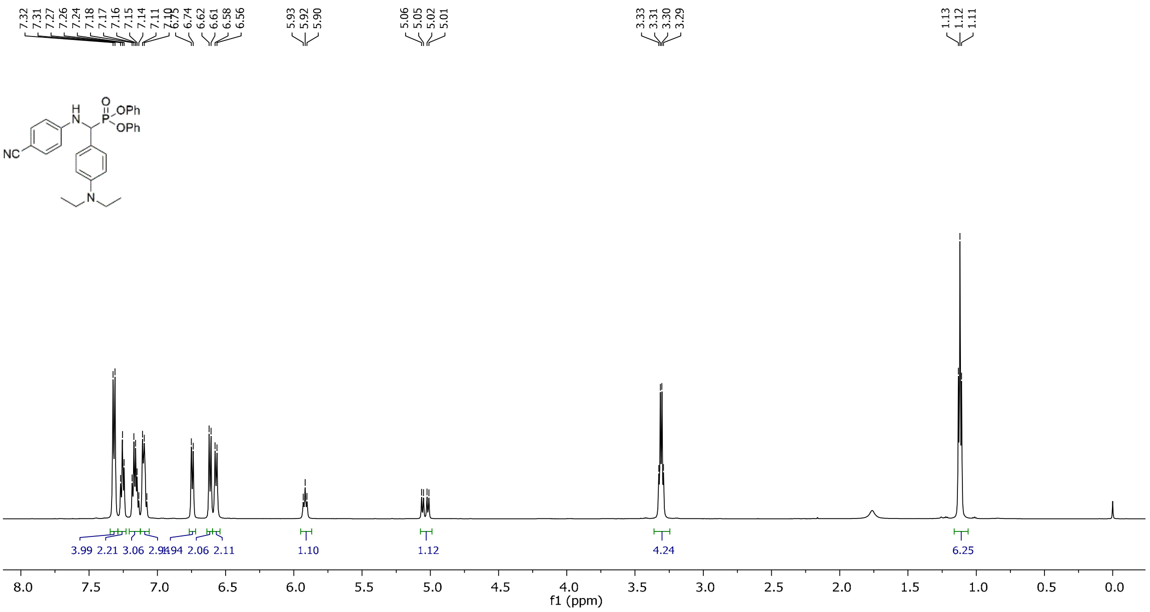

Figure S45. ^{1}H NMR (600 MHz, $CDCl_3$) of diphenyl (((4-cyanophenyl)amino)(4-(diethylamino)phenyl)methyl)phosphonate (**16**)

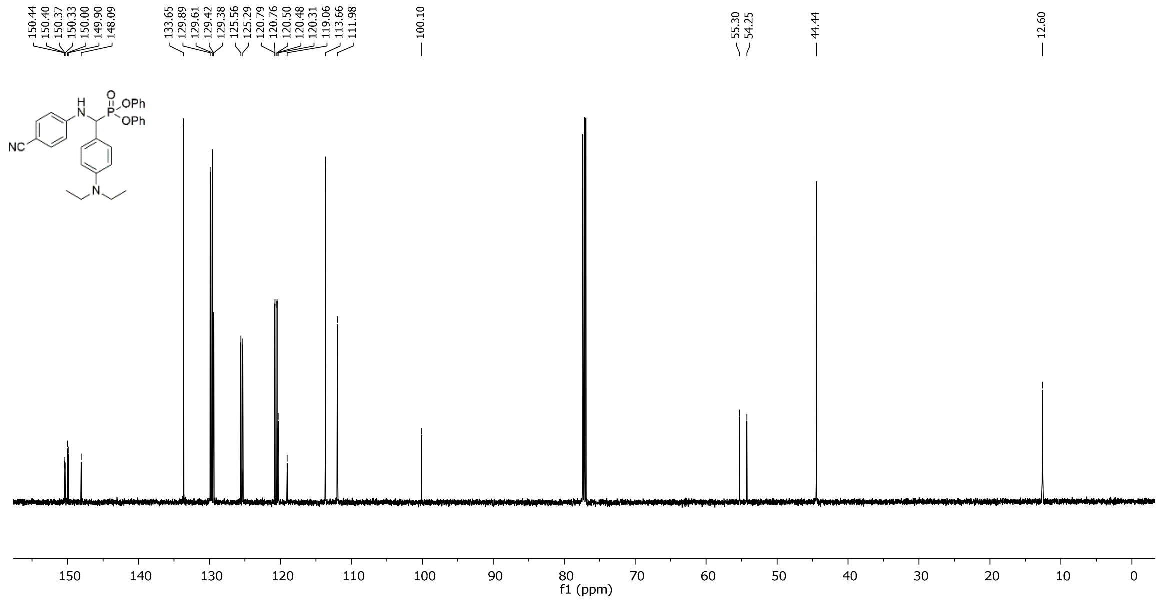

Figure S46. ^{13}C NMR (151 MHz, $CDCl_3$) of diphenyl (((4-cyanophenyl)amino)(4-(diethylamino)phenyl)methyl)phosphonate (**16**)

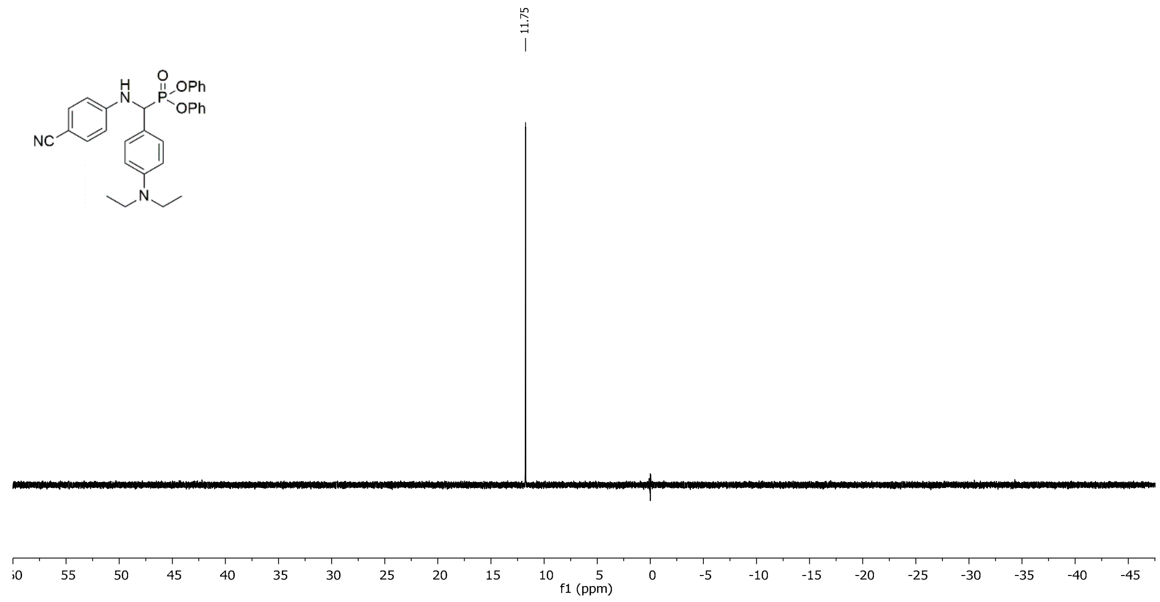

Figure S47. ^{31}P NMR (243 MHz, $CDCl_3$) of diphenyl (((4-cyanophenyl)amino)(4-(diethylamino)phenyl)methyl)phosphonate (**16**)

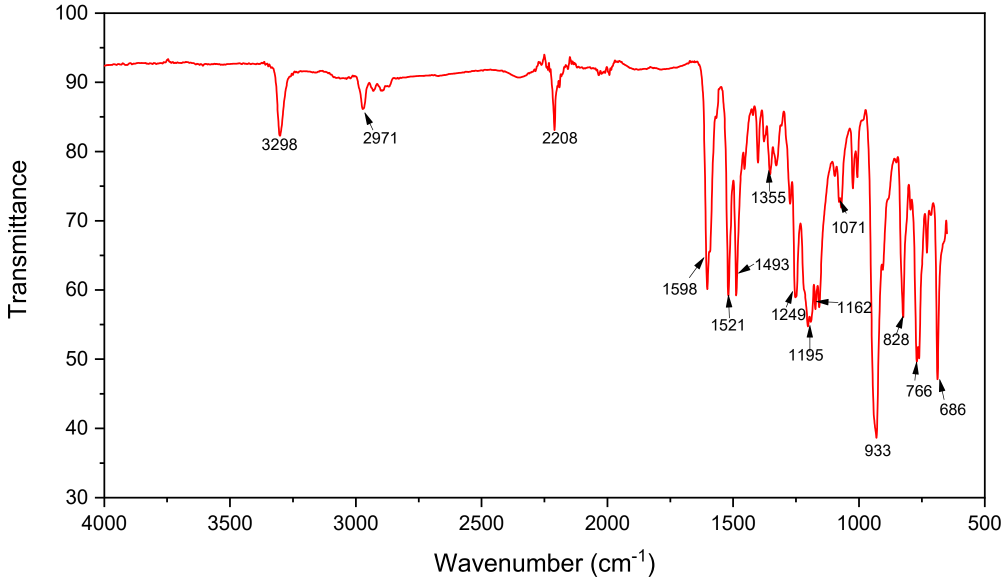

Figure S48. IR spectrum of diphenyl (((4-cyanophenyl)amino)(4-(diethylamino)phenyl)methyl)phosphonate (**16**)

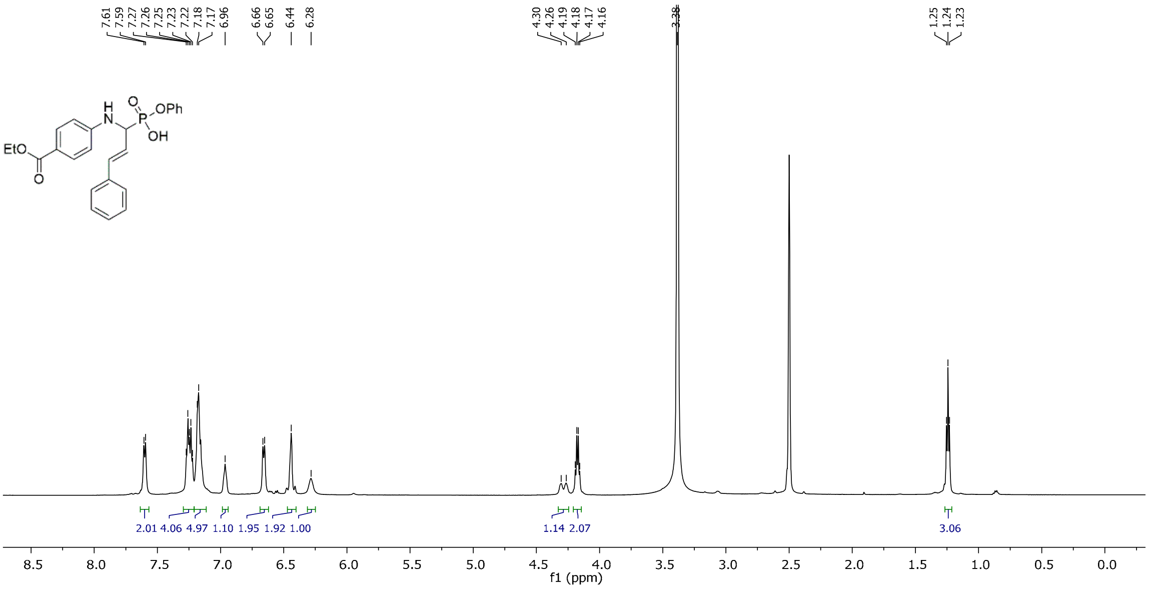

Figure S49. ¹H NMR (600 MHz, DMSO-d_6) of (*E*)-ethyl 4-((1-(hydroxy(phenoxy)phosphoryl)-3-phenylallyl)amino)benzoate (**17**)

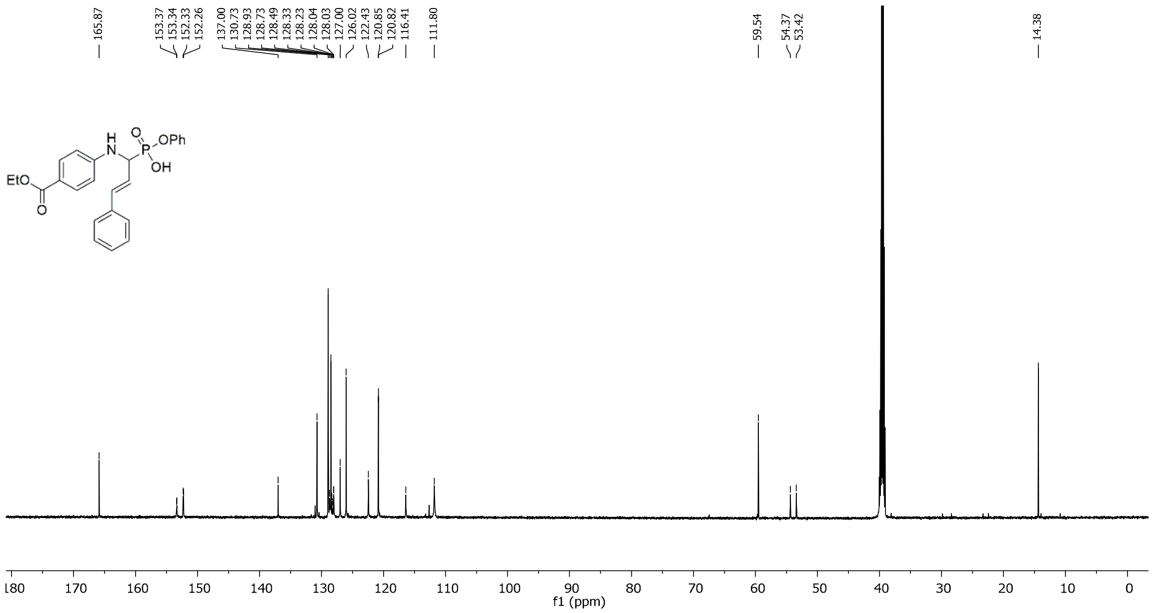

Figure S50. ^{13}C NMR (151 MHz, DMSO-d_6) of (*E*)-ethyl 4-((1-(hydroxy(phenoxy)phosphoryl)-3-phenylallyl)amino)benzoate (**17**)

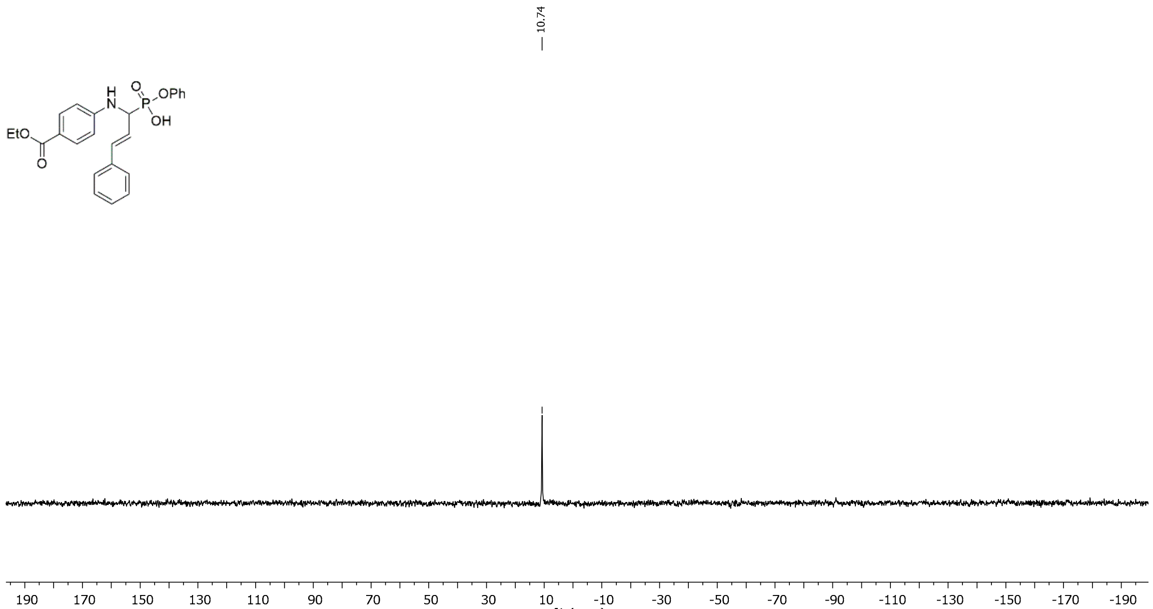

Figure S51. ^{31}P NMR (243 MHz, DMSO-d_6) of (*E*)-ethyl 4-((1-(hydroxy(phenoxy)phosphoryl)-3-phenylallyl)amino)benzoate (**17**)

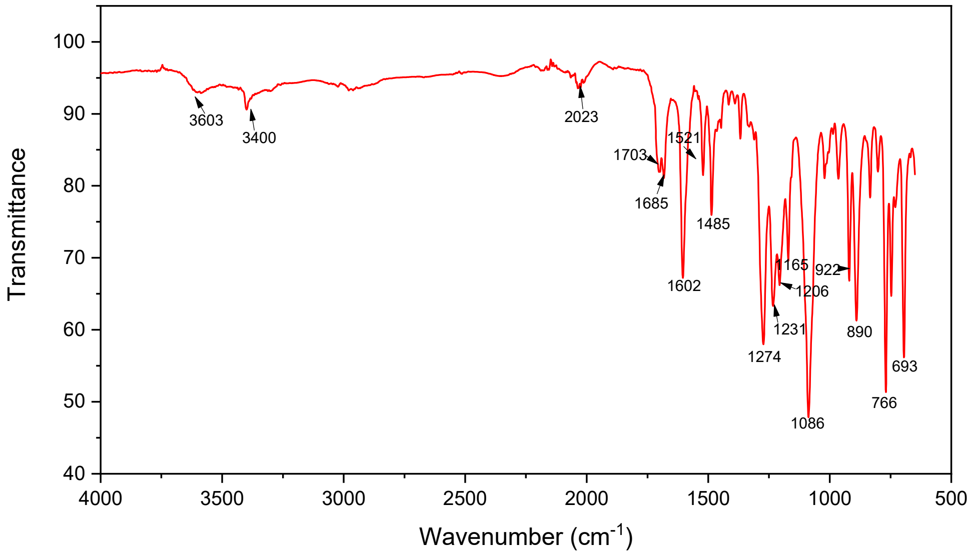

Figure S52. IR spectrum of (*E*)-ethyl 4-((1-(hydroxy(phenoxy)phosphoryl)-3-phenylallyl)amino)benzoate (**17**)

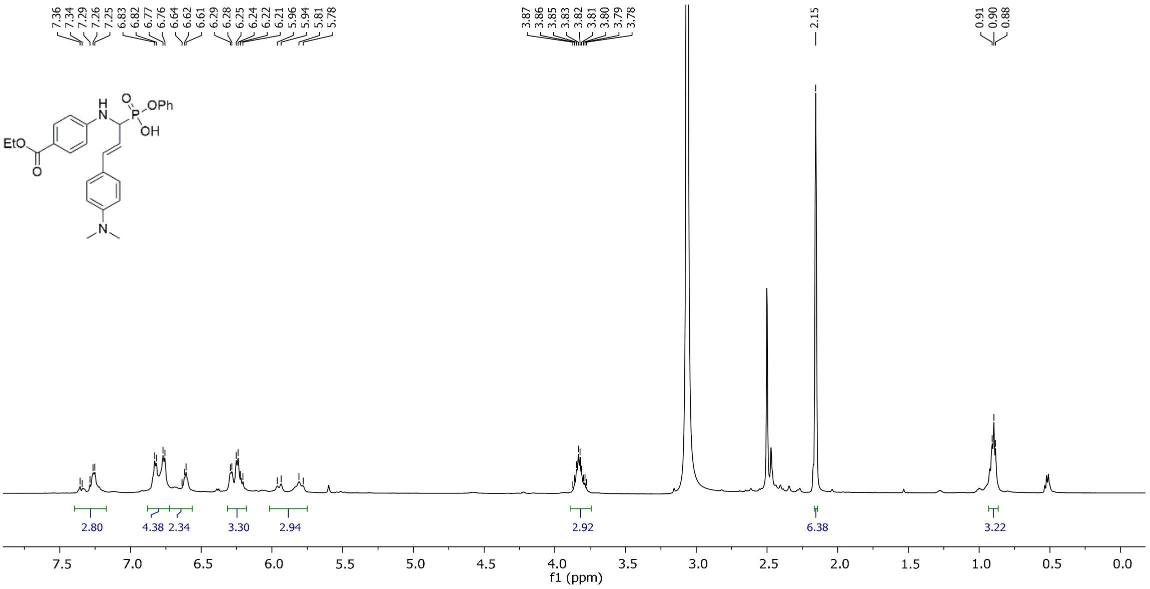

Figure S53. ¹H NMR (600 MHz, DMSO-d_6) of (*E*)-ethyl 4-((3-(4-(dimethylamino)phenyl)-1-(hydroxy(phenoxy)phosphoryl)allyl)amino)benzoate (**18**)

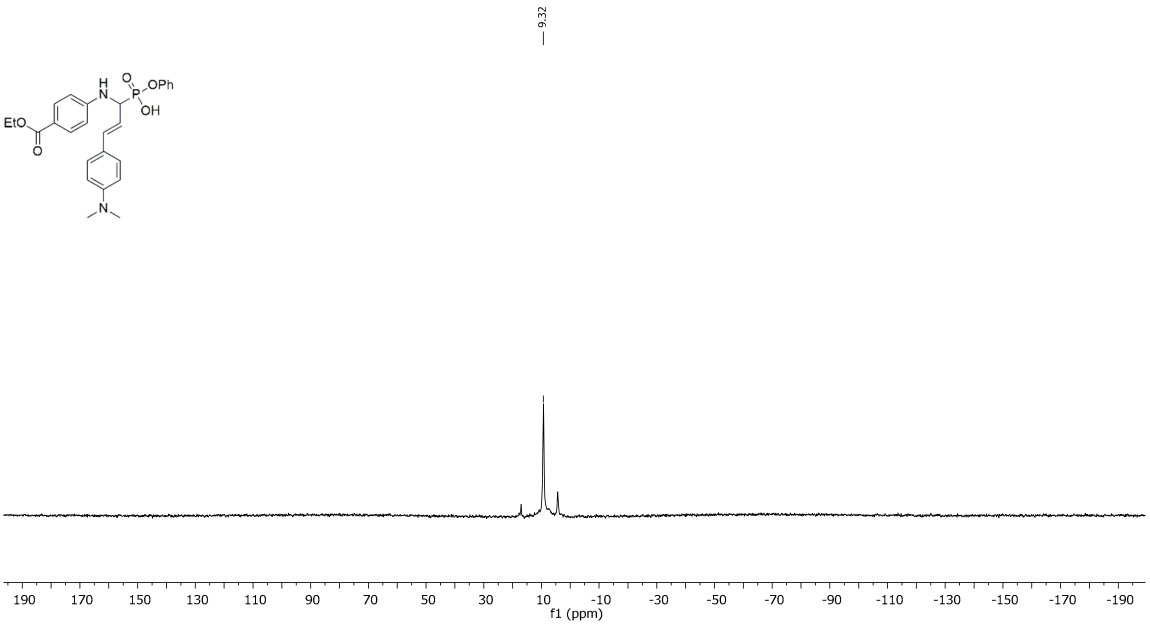

Figure S54. ^{31}P NMR (243 MHz, DMSO-d_6) of (*E*)-ethyl 4-((3-(4-(dimethylamino)phenyl)-1-(hydroxy(phenoxy)phosphoryl)allyl)amino)benzoate (**18**)

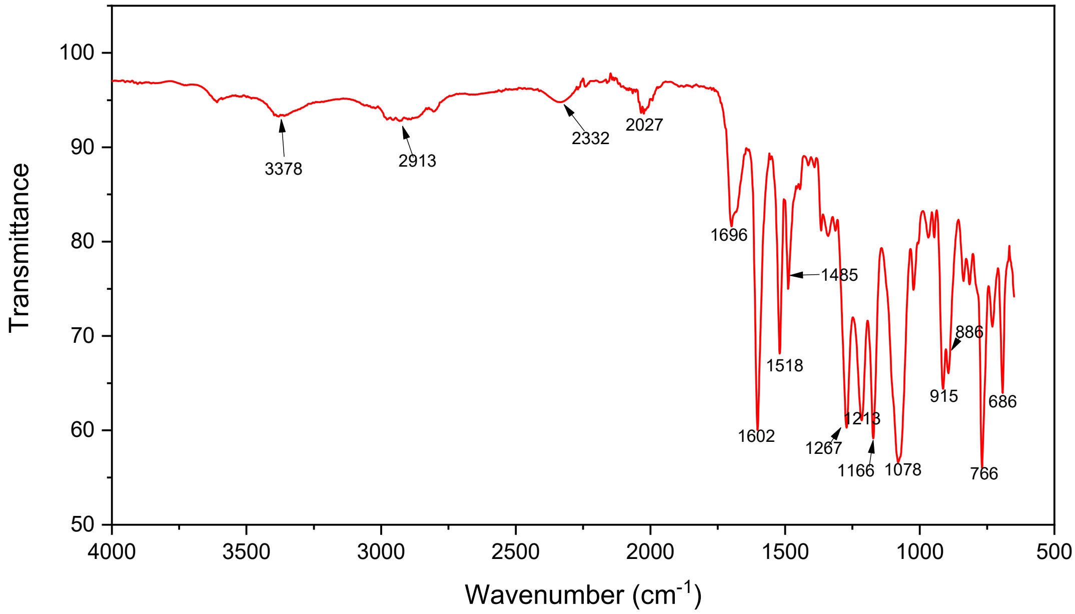

Figure S55. IR spectrum of (*E*)-ethyl 4-((3-(4-(dimethylamino)phenyl)-1-(hydroxy(phenoxy)phosphoryl)allyl)amino)benzoate (**18**)

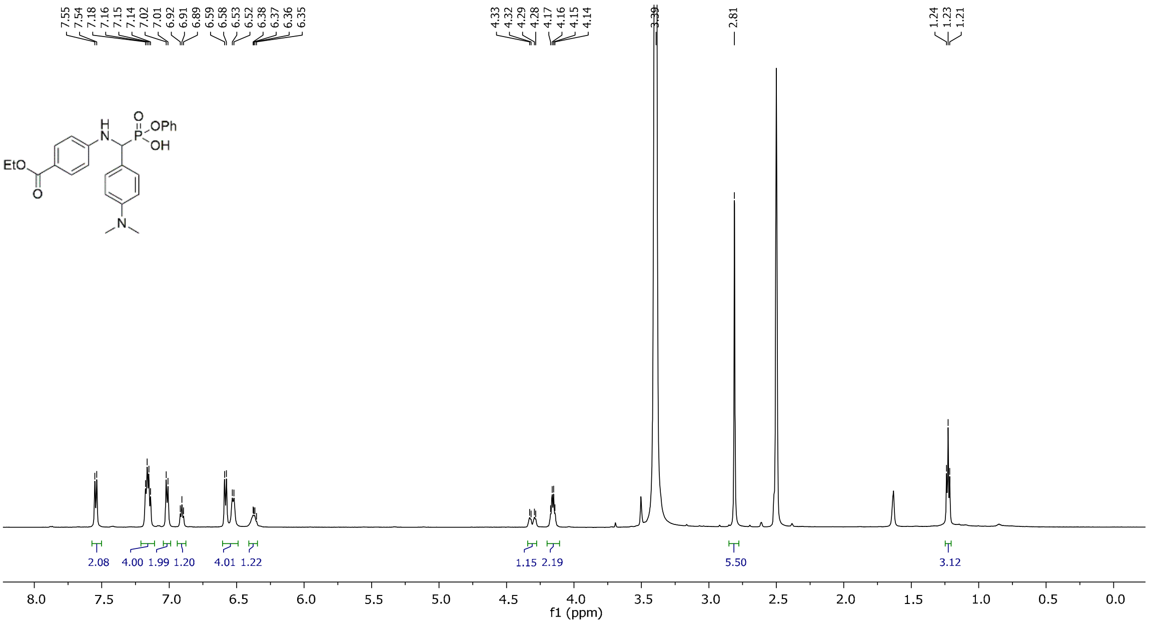

Figure S56. ^{1}H NMR (600 MHz, DMSO-d_6) of ethyl 4-(((4-(dimethylamino)phenyl)(hydroxy(phenoxy)phosphoryl)methyl)amino)benzoate (**19**)

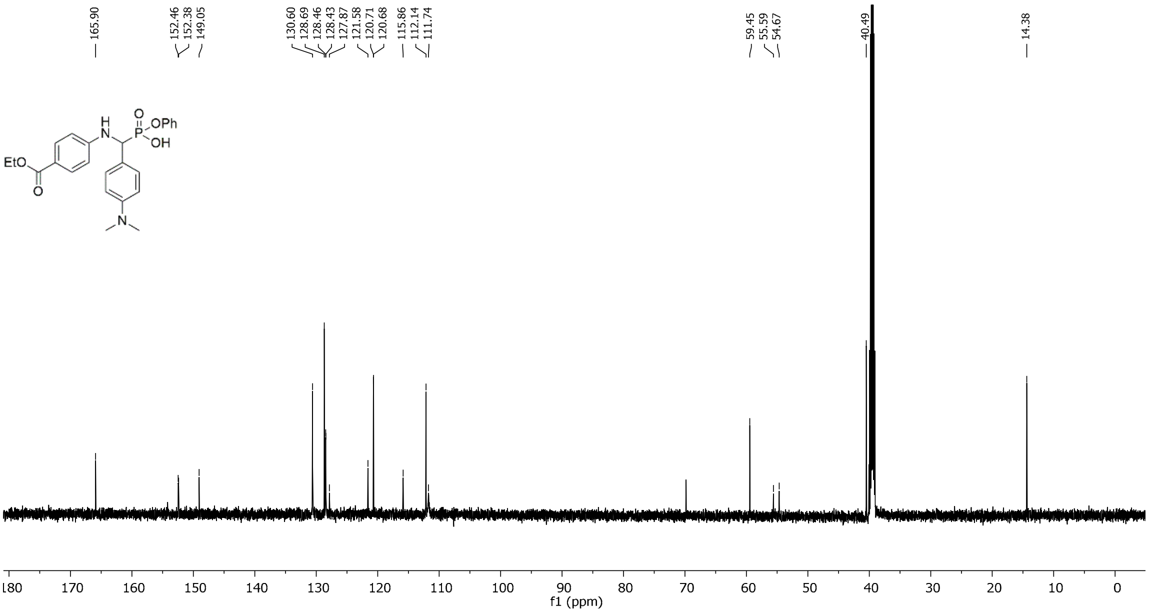

Figure S57. ^{13}C NMR (151 MHz, DMSO-d_6) of ethyl 4-(((4-(dimethylamino)phenyl)(hydroxy(phenoxy)phosphoryl)methyl)amino)benzoate (**19**)

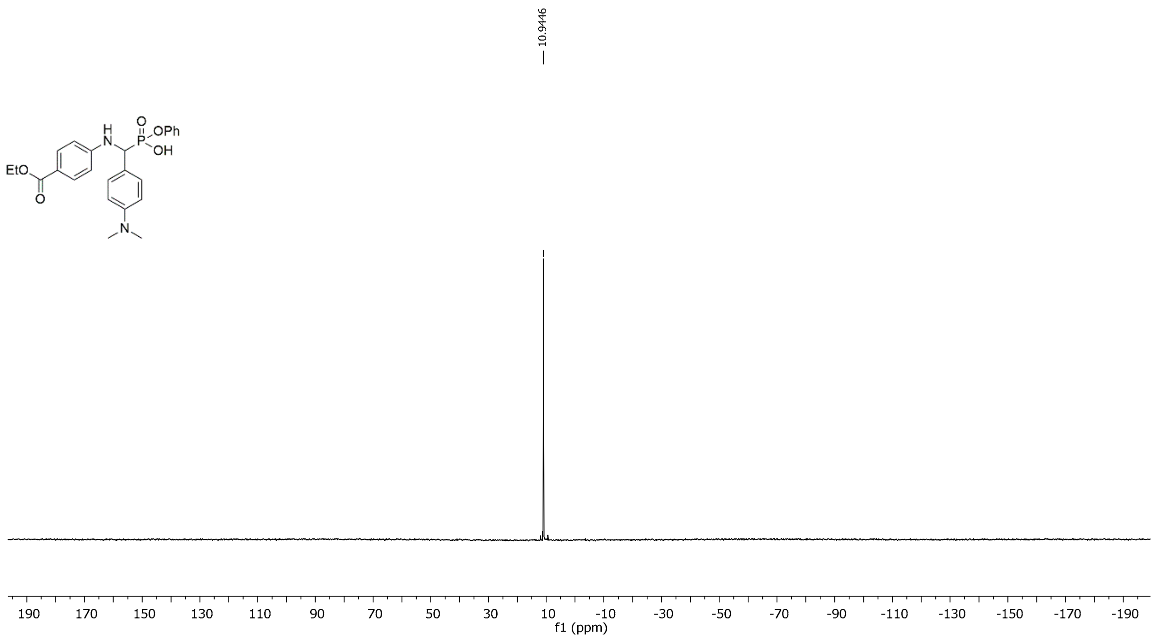

Figure S58. ^{31}P NMR (243 MHz, DMSO-d_6) of ethyl 4-(((4-(dimethylamino)phenyl)(hydroxy(phenoxy)phosphoryl)methyl)amino)benzoate (**19**)

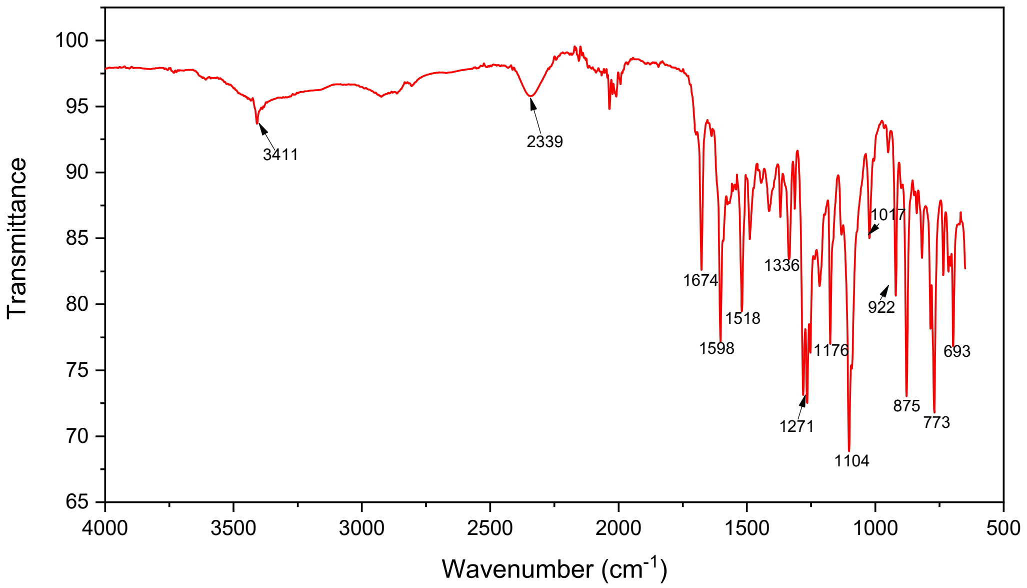

Figure S59. IR spectrum of ethyl 4-(((4-(dimethylamino)phenyl)(hydroxy(phenoxy)phosphoryl)methyl)amino)benzoate (**19**)

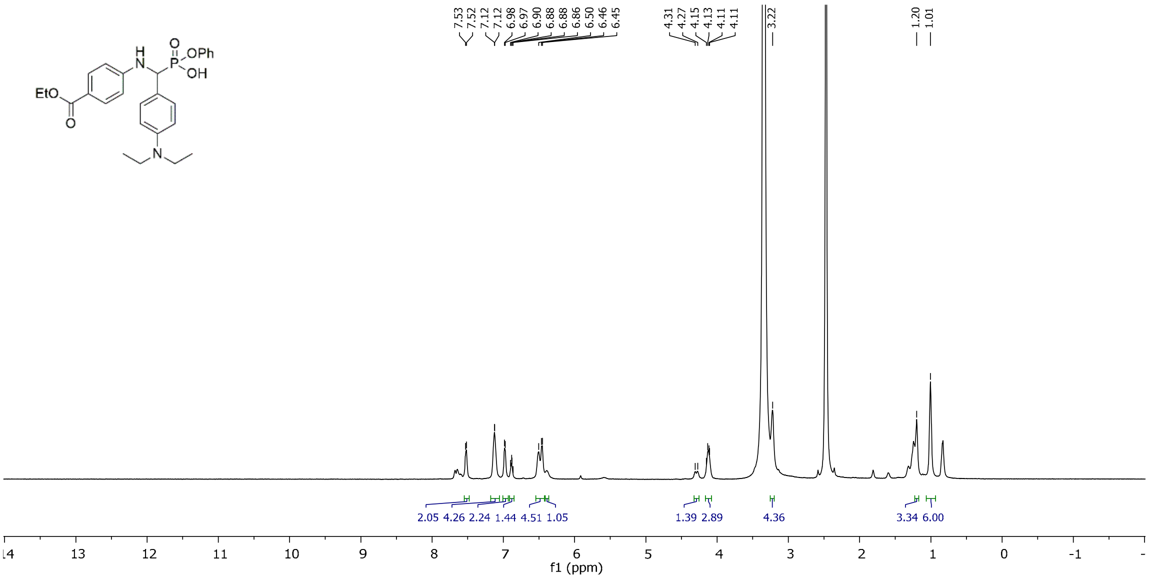

Figure S60. ^{1}H NMR (600 MHz, DMSO-d_6) of ethyl 4-(((4-(diethylamino)phenyl)(hydroxy(phenoxy)phosphoryl)methyl)amino)benzoate (**20**)

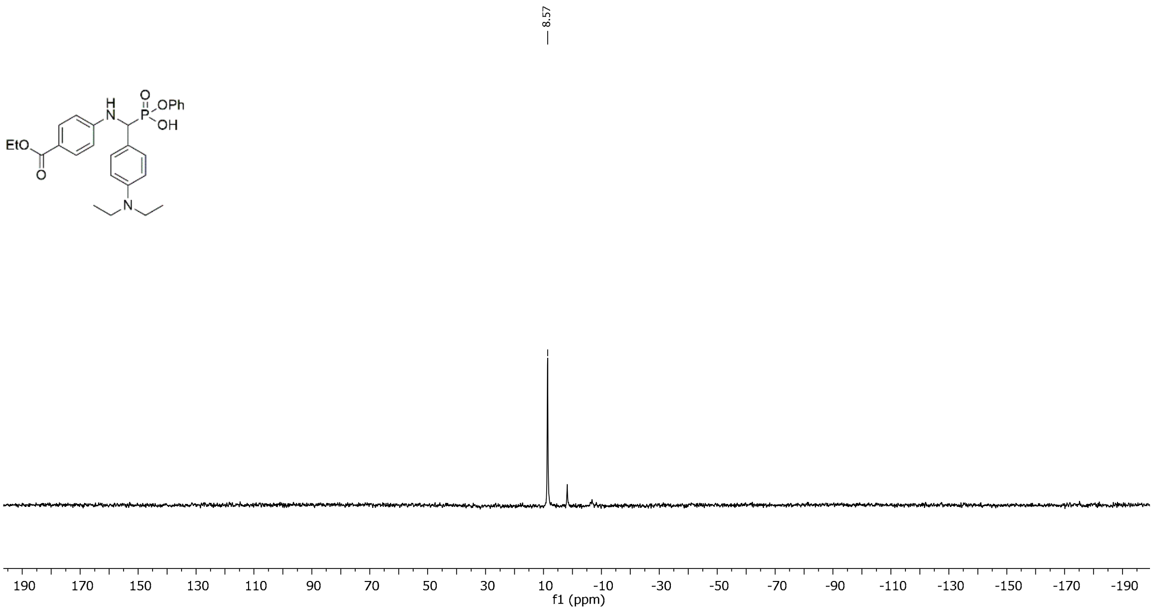

Figure S61. ^{31}P NMR (243 MHz, DMSO-d_6) of ethyl 4-(((4-(diethylamino)phenyl)(hydroxy(phenoxy)phosphoryl)methyl)amino)benzoate (**20**)

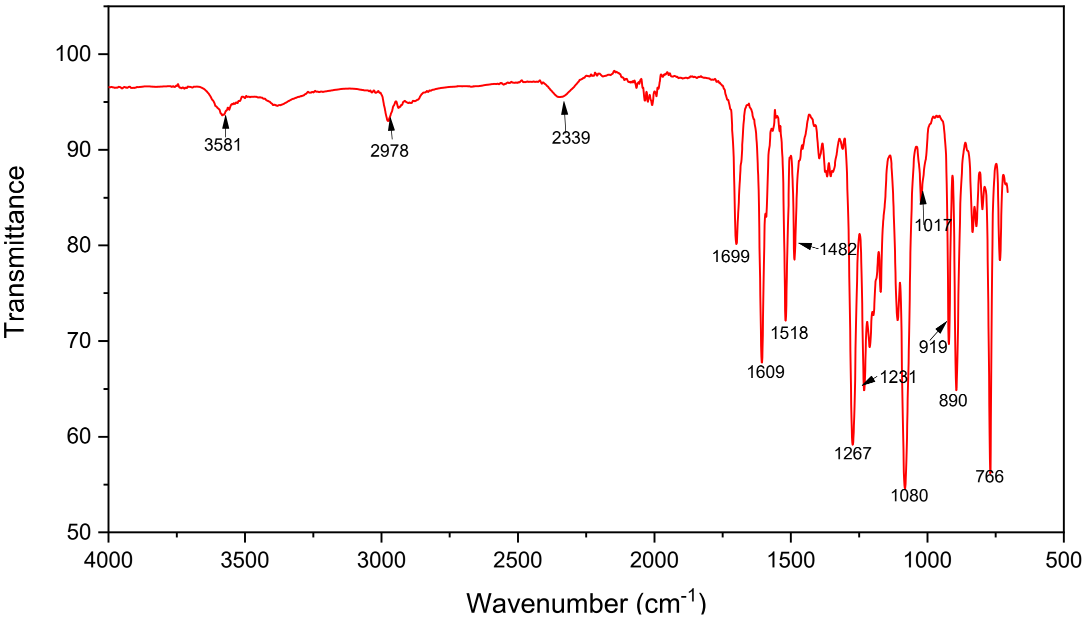

Figure S62. IR spectrum of ethyl 4-(((4-(diethylamino)phenyl)(hydroxy(phenoxy)phosphoryl)methyl)amino)benzoate (**20**)

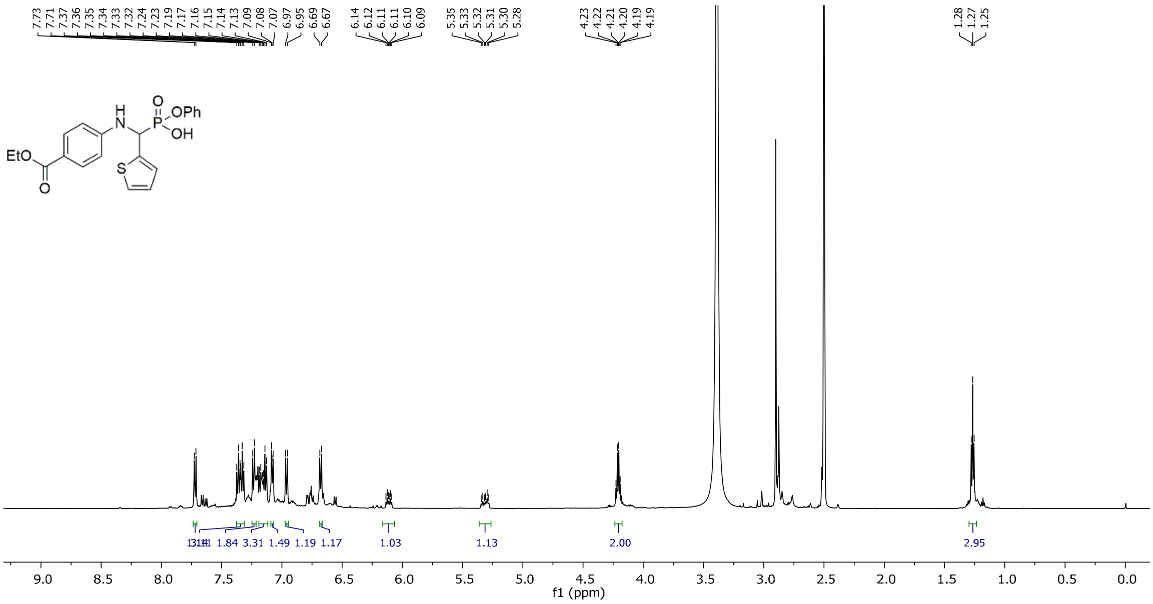

Figure S63. ^{1}H NMR (600 MHz, DMSO-d_6) of ethyl 4-(((hydroxy(phenoxy)phosphoryl)(thiophen-2-yl)methyl)amino)benzoate (**21**)

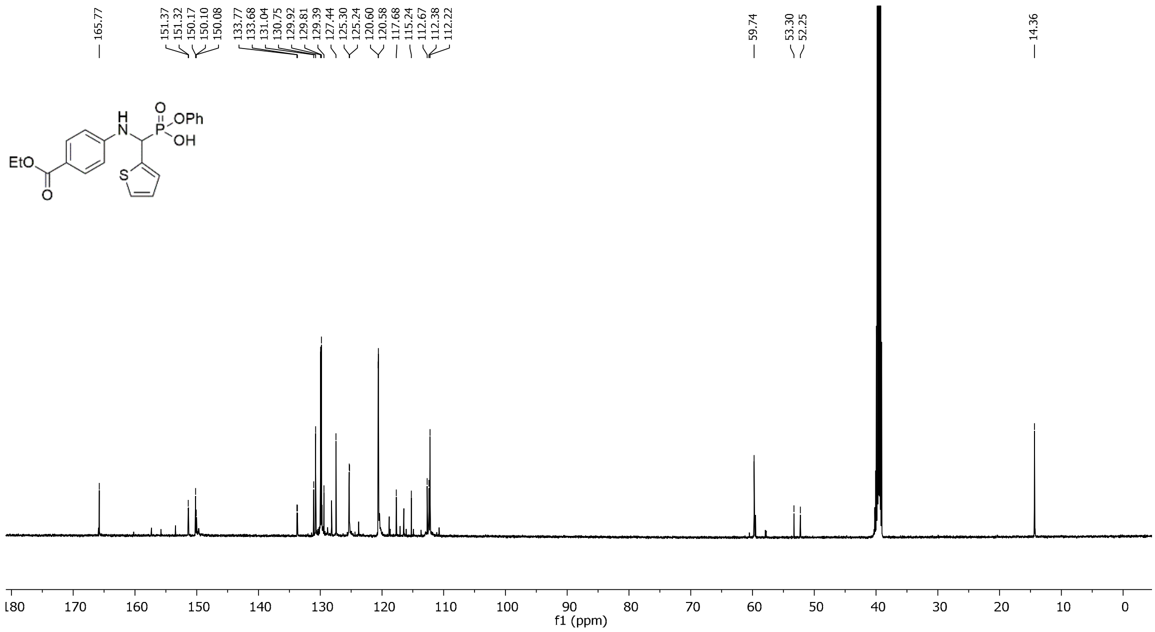

Figure S64. ^{13}C NMR (176 MHz, DMSO-d_6) of ethyl 4-(((hydroxy(phenoxy)phosphoryl)(thiophen-2-yl)methyl)amino)benzoate (**21**)

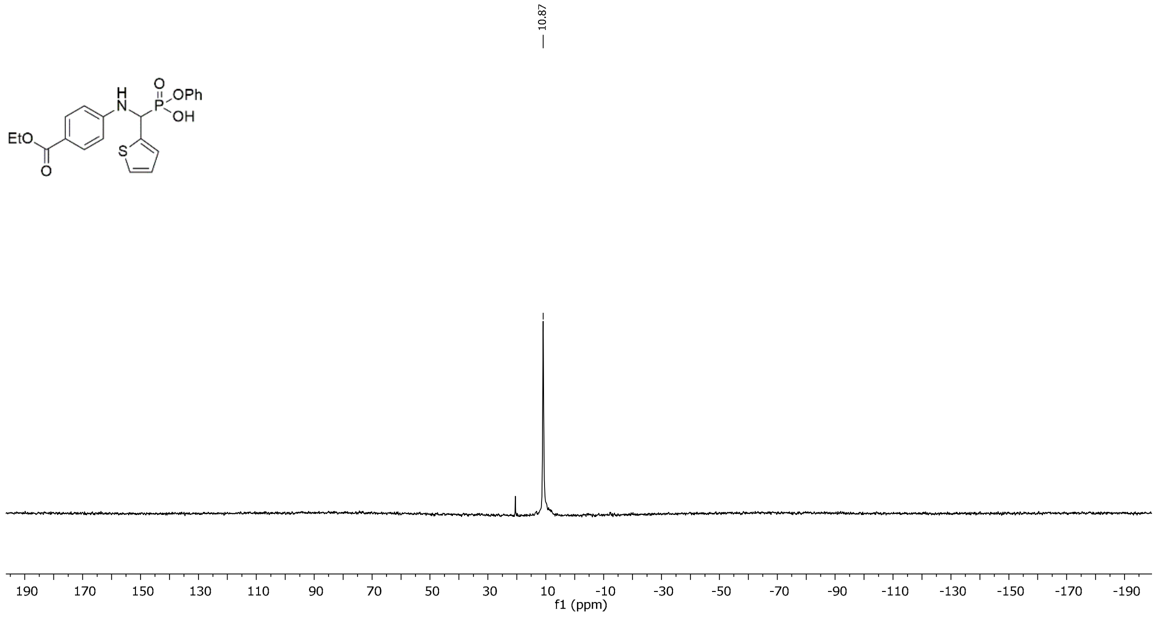

Figure S65. ^{31}P NMR (243 MHz, DMSO-d_6) of ethyl 4-(((hydroxy(phenoxy)phosphoryl)(thiophen-2-yl)methyl)amino)benzoate (**21**)

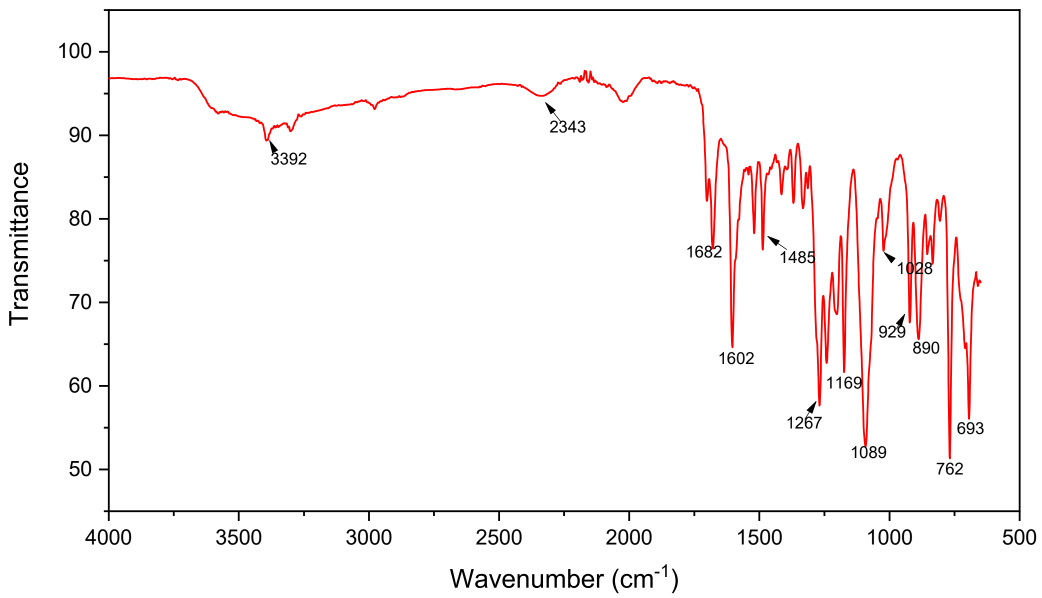

Figure S66. IR spectrum of ethyl 4-(((hydroxy(phenoxy)phosphoryl)(thiophen-2-yl)methyl)amino)benzoate (**21**)

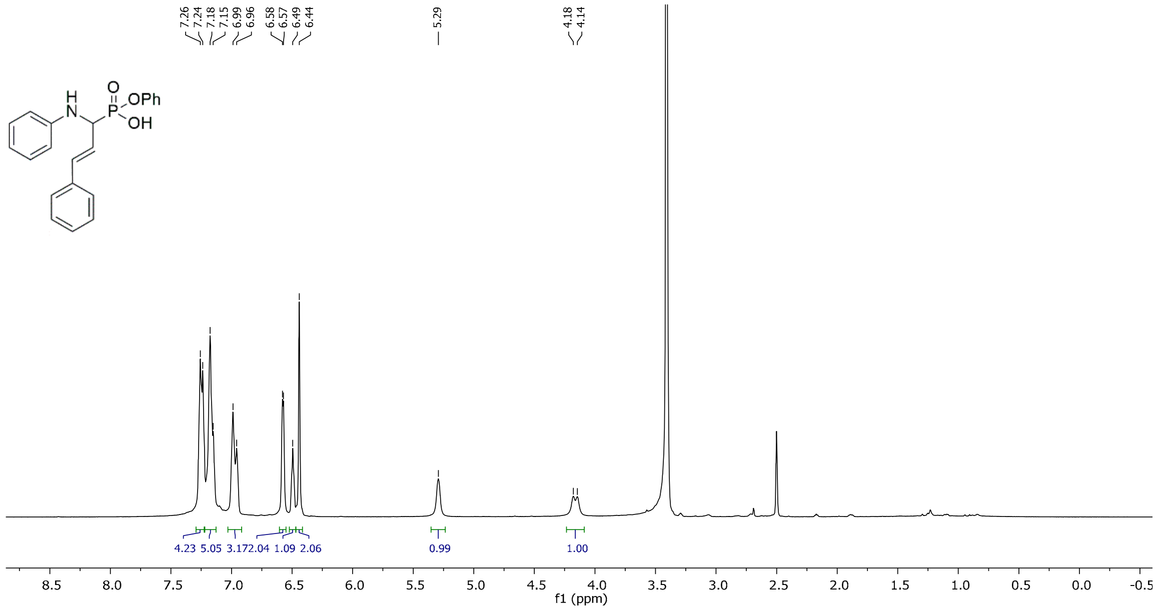

Figure S67. ^{1}H NMR (700 MHz, DMSO-d_6) of (*E*)-phenyl hydrogen (3-phenyl-1-(phenylamino)allyl)phosphonate (**22**)

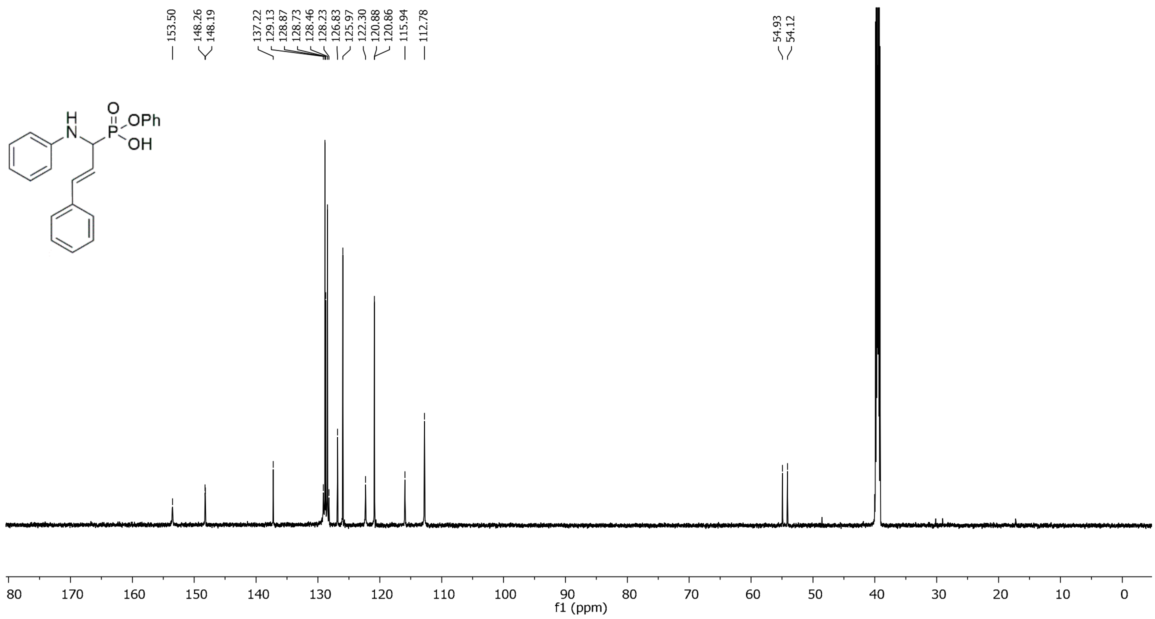

Figure S68. ^{13}C NMR (176 MHz, DMSO-d_6) of (*E*)-phenyl hydrogen (3-phenyl-1-(phenylamino)allyl)phosphonate (**22**)

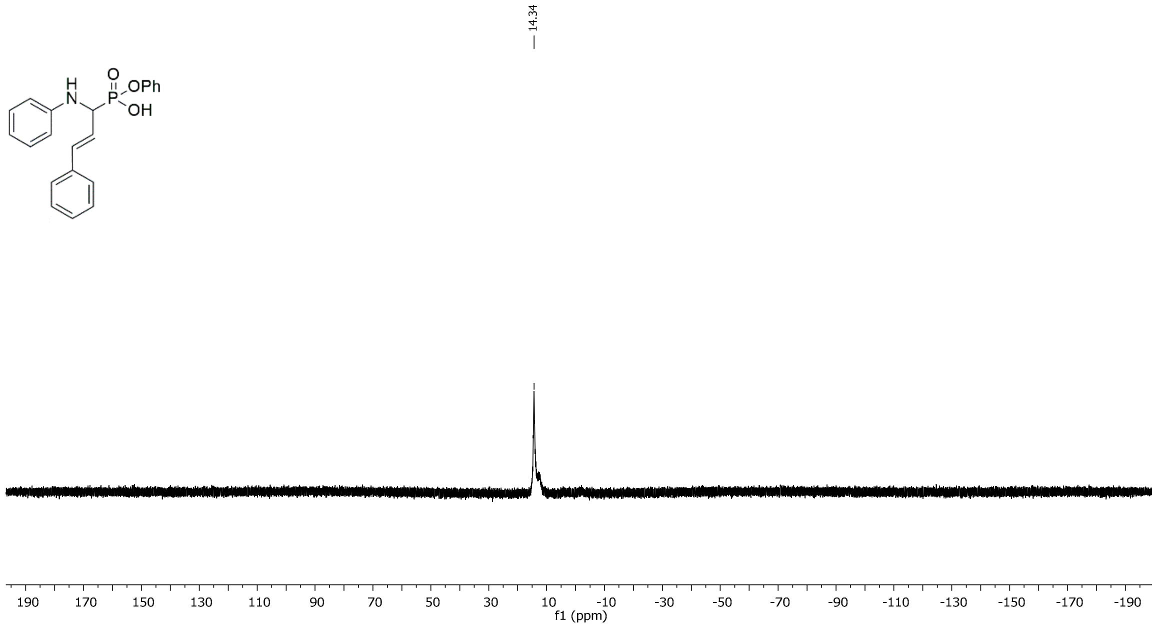

Figure S69. ^{31}P NMR (243 MHz, DMSO-d_6) of (*E*)-phenyl hydrogen (3-phenyl-1-(phenylamino)allyl)phosphonate (**22**)

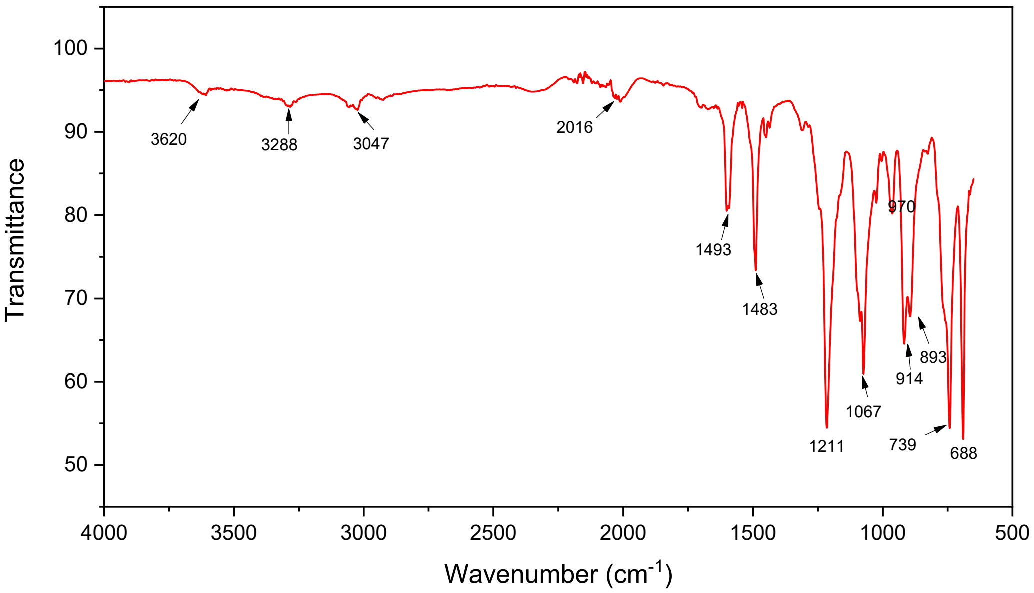

Figure S70. IR spectrum of (*E*)-phenyl hydrogen (3-phenyl-1-(phenylamino)allyl)phosphonate (**22**)

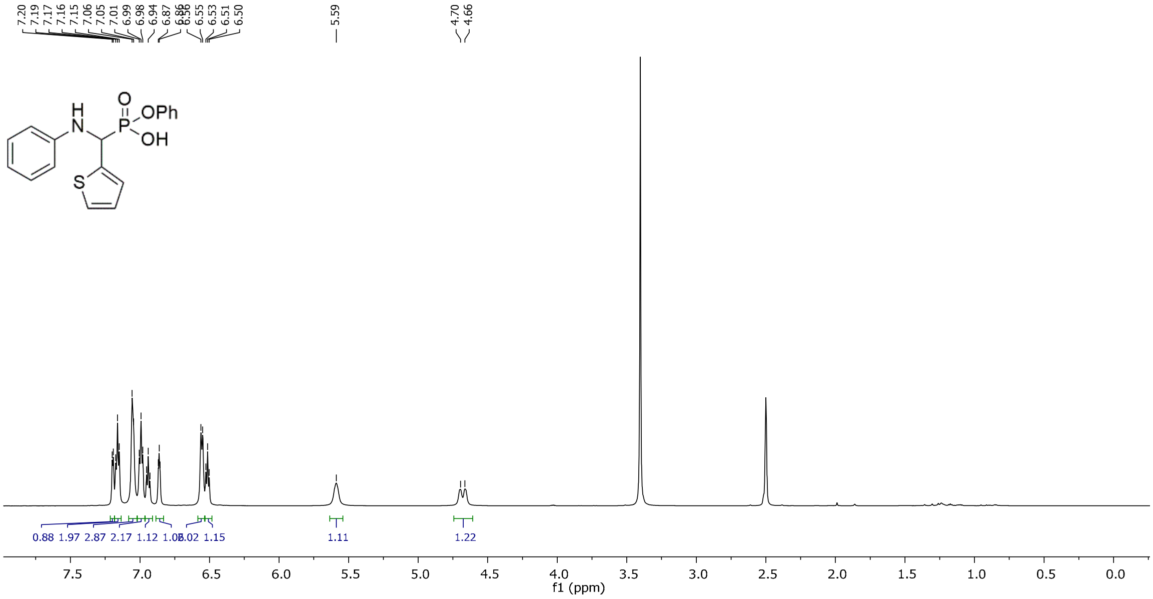

Figure S71. ^{1}H NMR (600 MHz, DMSO-d_6) of phenyl hydrogen ((phenylamino)(thiophen-2-yl)methyl)phosphonate (**23**)

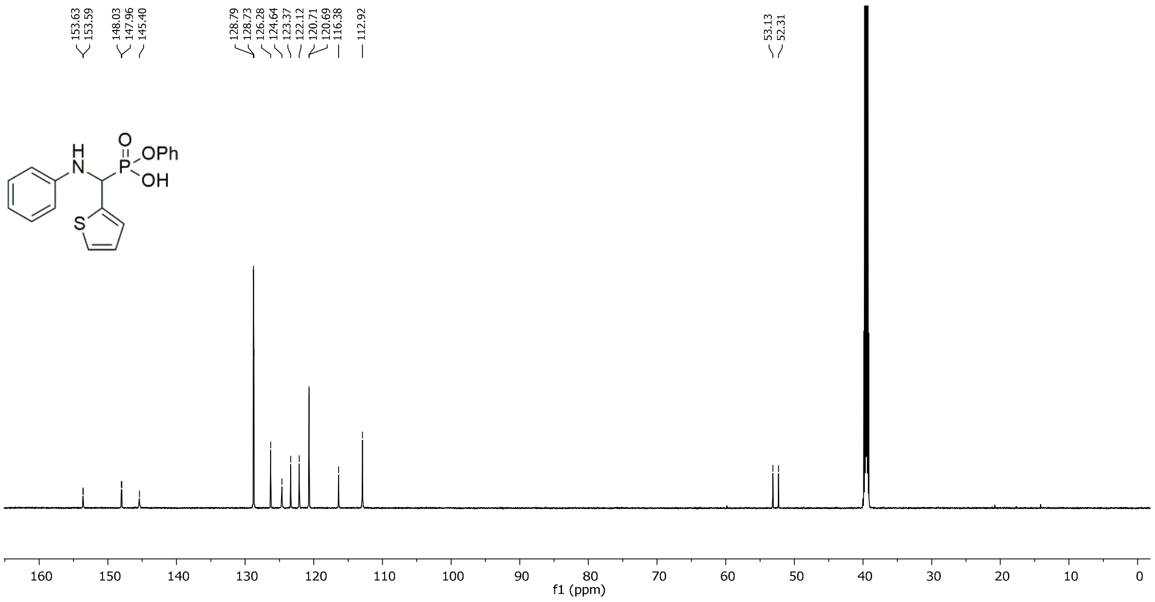

Figure S72. ^{13}C NMR (176 MHz, DMSO-d_6) of phenyl hydrogen ((phenylamino)(thiophen-2-yl)methyl)phosphonate (**23**)

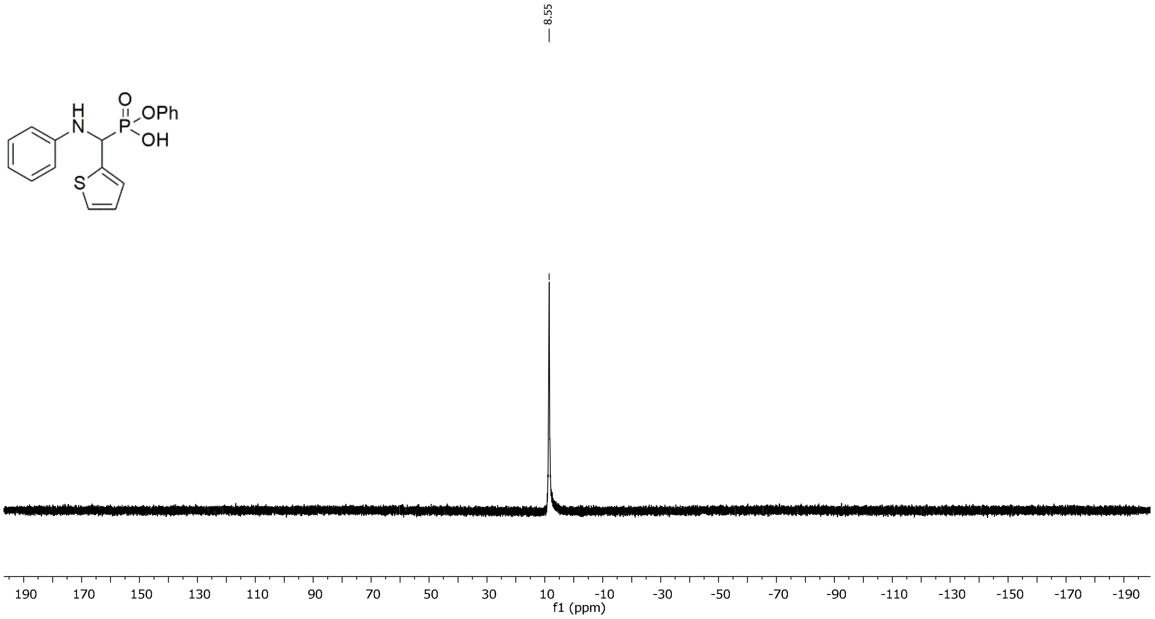

Figure S73. ^{31}P NMR (243 MHz, DMSO-d_6) of phenyl hydrogen ((phenylamino)(thiophen-2-yl)methyl)phosphonate (**23**)

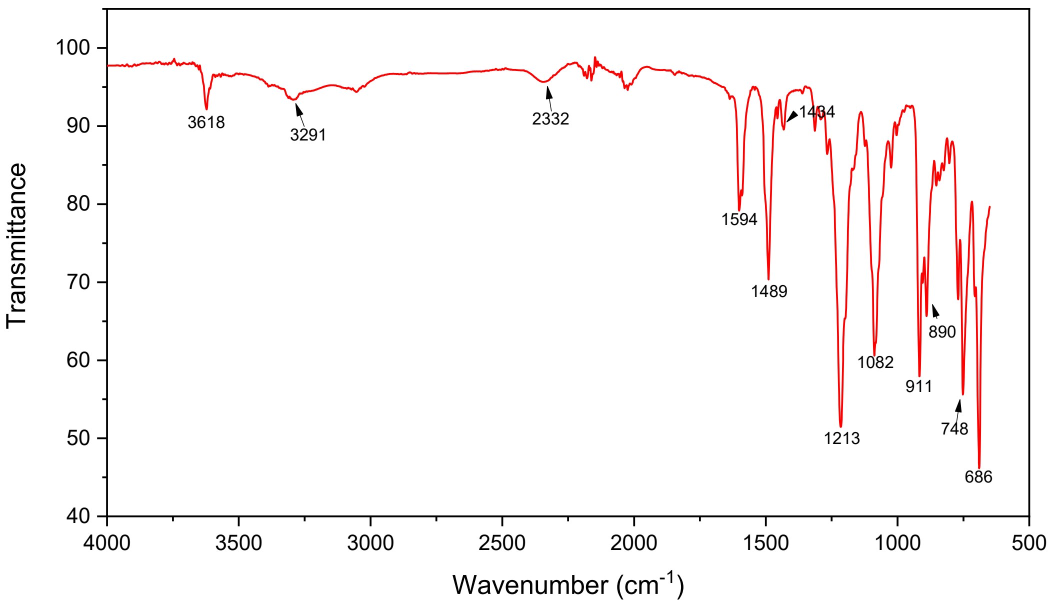

Figure S74. IR spectrum of phenyl hydrogen ((phenylamino)(thiophen-2-yl)methyl)phosphonate (**23**)

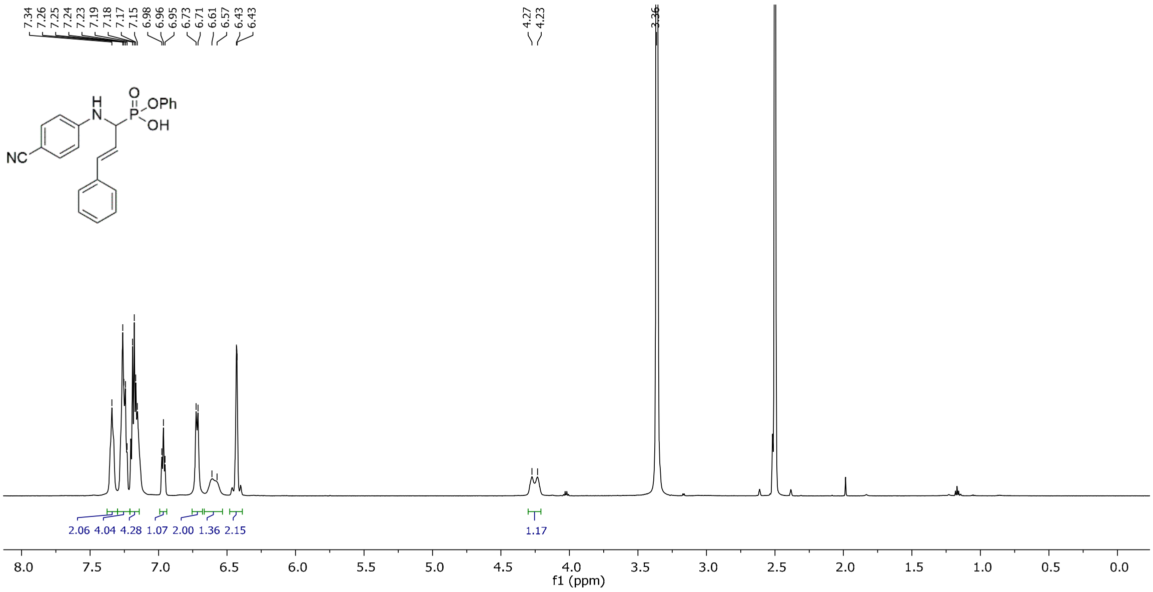

Figure S75. ¹H NMR (600 MHz, DMSO-d_6) of (E)-phenyl hydrogen (1-((4-cyanophenyl)amino)-3-phenylallyl)phosphonate (**24**)

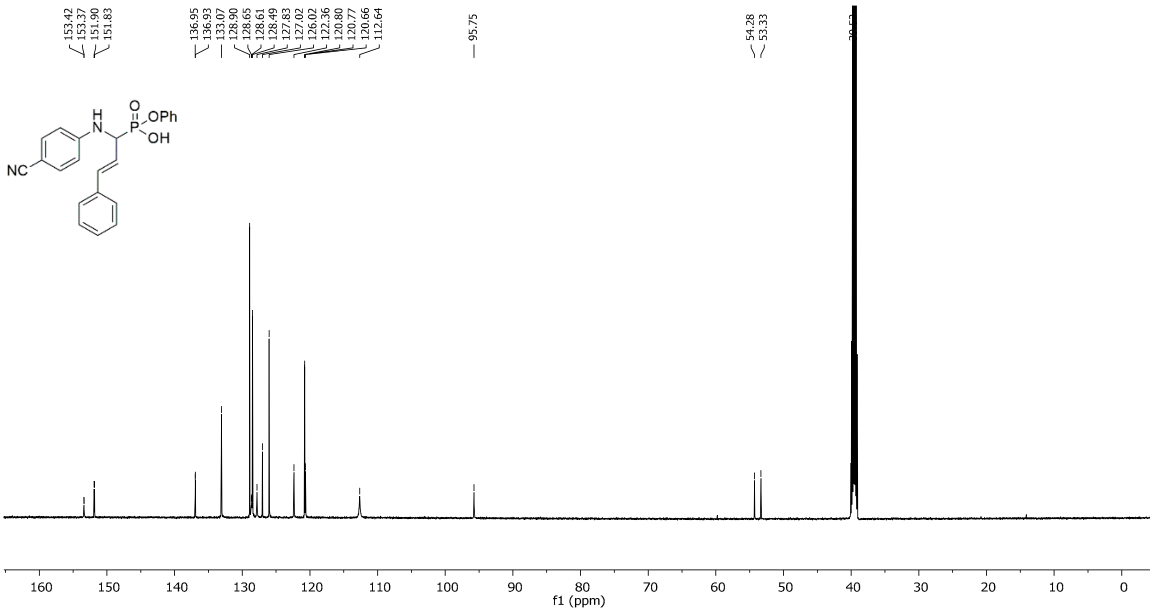

Figure S76. ^{13}C NMR (151 MHz, DMSO-d_6) of (*E*)-phenyl hydrogen (1-((4-cyanophenyl)amino)-3-phenylallyl)phosphonate (**24**)

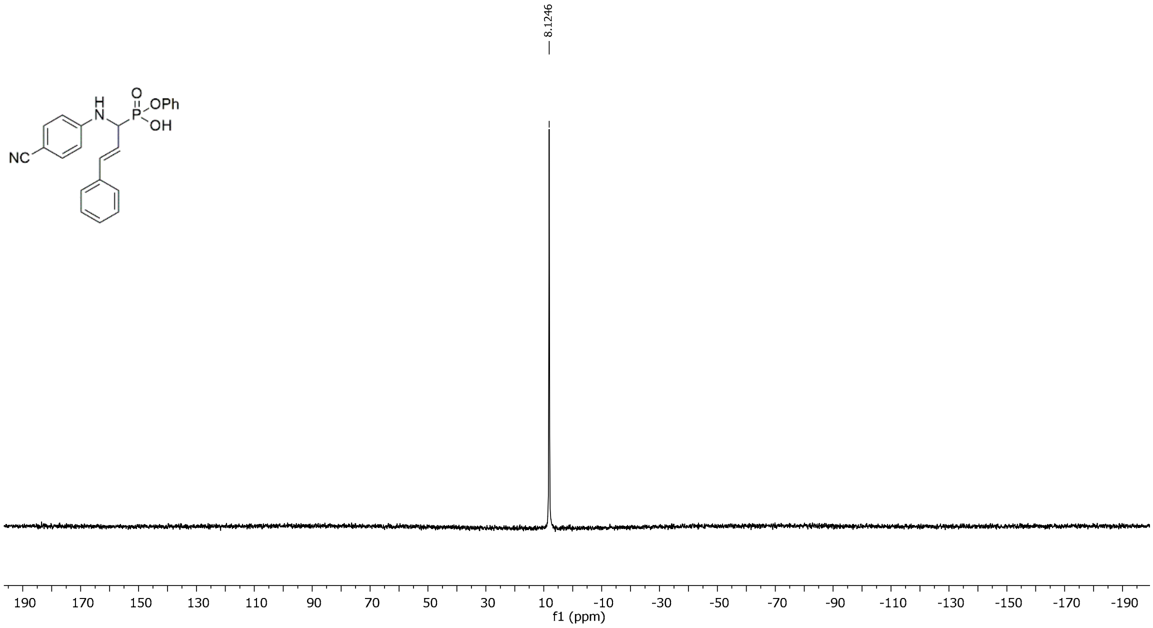

Figure S77. ^{31}P NMR (243 MHz, DMSO-d_6) of (*E*)-phenyl hydrogen (1-((4-cyanophenyl)amino)-3-phenylallyl)phosphonate (**24**)

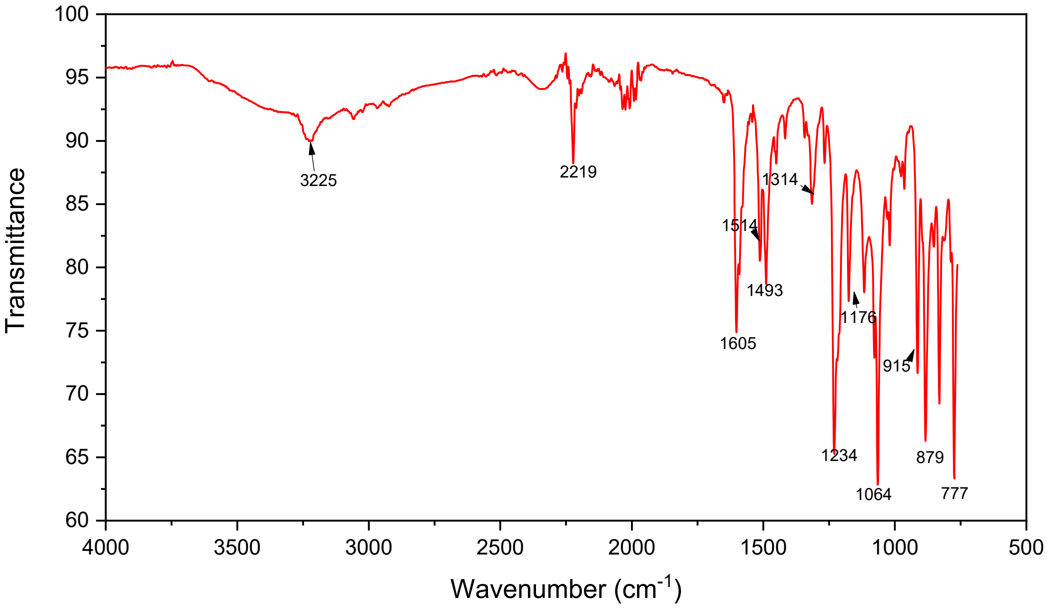

Figure S78. IR spectrum of (*E*)-phenyl hydrogen (1-((4-cyanophenyl)amino)-3-phenylallyl)phosphonate (**24**)

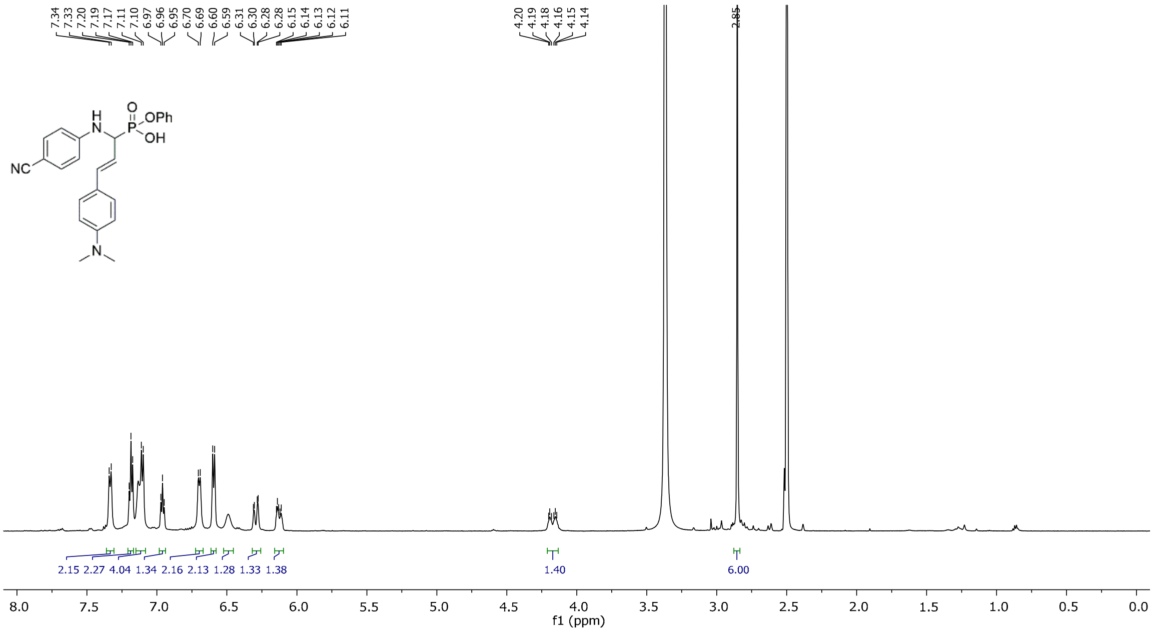

Figure S79. ^{1}H NMR (600 MHz, DMSO-d_6) of (*E*)-phenyl hydrogen (1-((4-cyanophenyl)amino)-3-(4-(dimethylamino)phenyl)allyl)phosphonate (**25**)

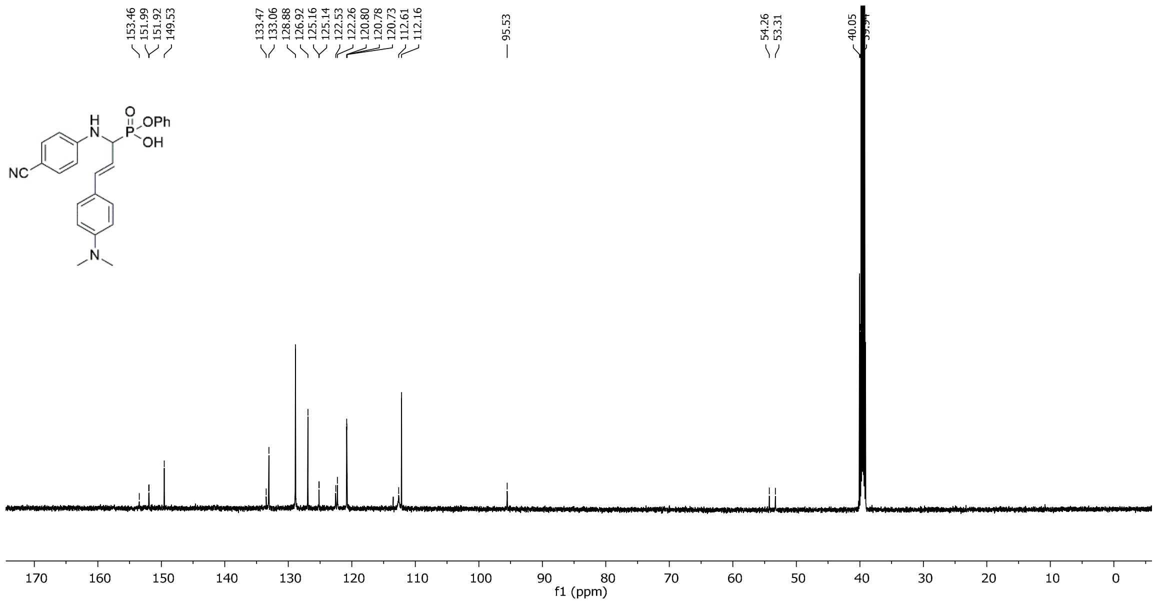

Figure S80. ^{13}C NMR (151 MHz, DMSO-d_6) of (*E*)-phenyl hydrogen (1-((4-cyanophenyl)amino)-3-(4-(dimethylamino)phenyl)allyl)phosphonate (**25**)

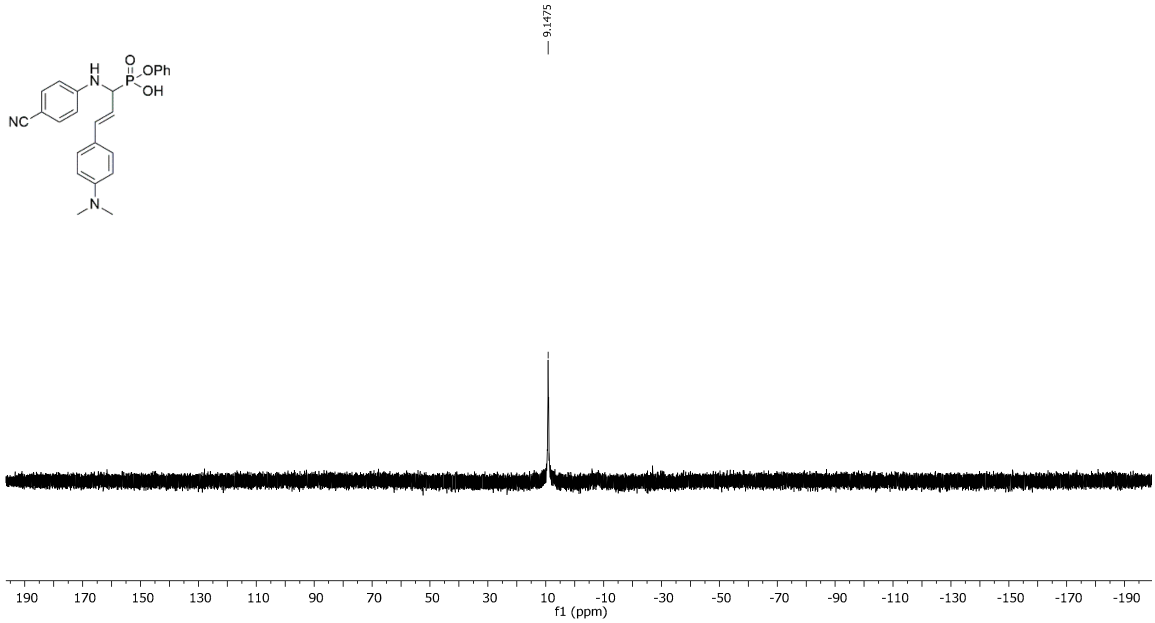

Figure S81. ^{31}P NMR (243 MHz, DMSO-d_6) of (*E*)-phenyl hydrogen (1-((4-cyanophenyl)amino)-3-(4-(dimethylamino)phenyl)allyl)phosphonate (**25**)

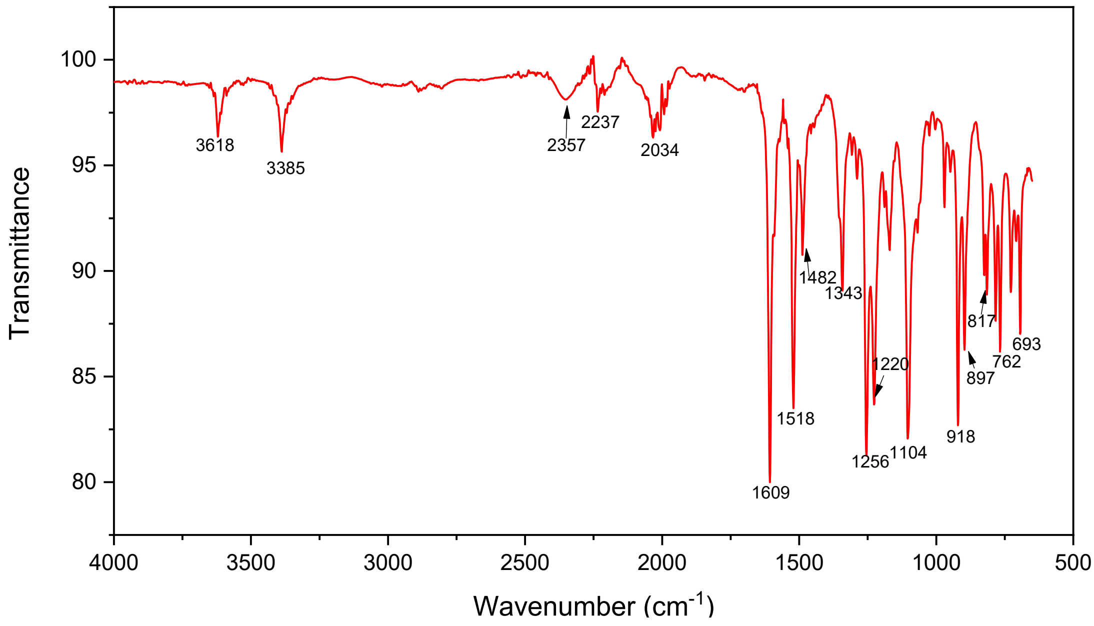

Figure S82. IR spectrum of (*E*)-phenyl hydrogen (1-((4-cyanophenyl)amino)-3-(4-(dimethylamino)phenyl)allyl)phosphonate (**25**)

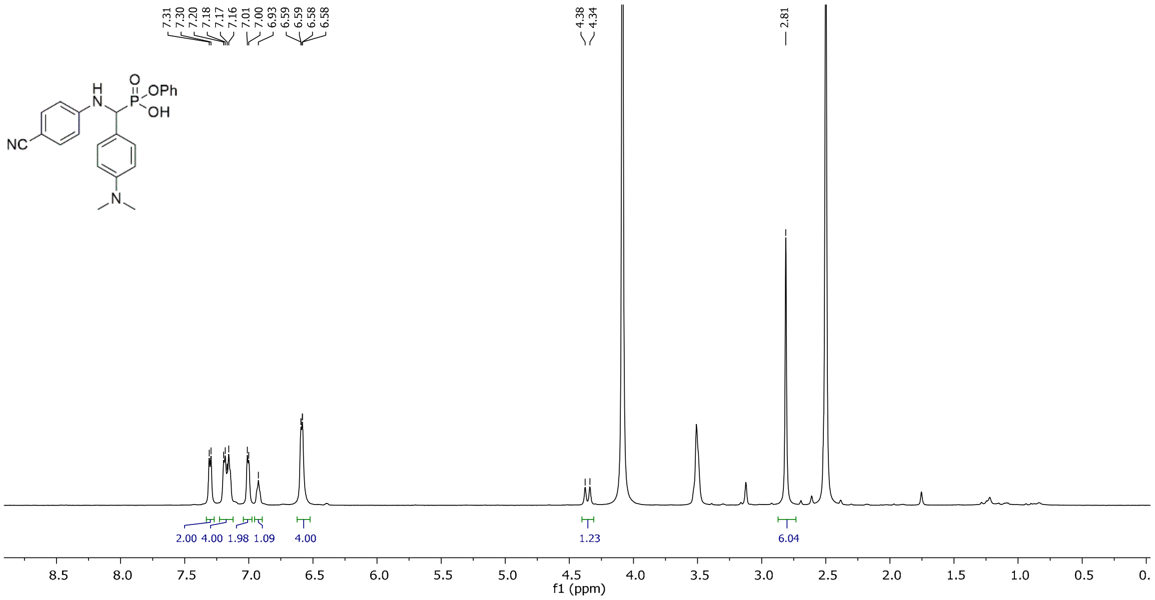

Figure S83. ^{1}H NMR (600 MHz, CD_3OD/DMSO-d_6) of phenyl hydrogen (((4-cyanophenyl)amino)(4-(dimethylamino)phenyl)methyl)phosphonate (**26**)

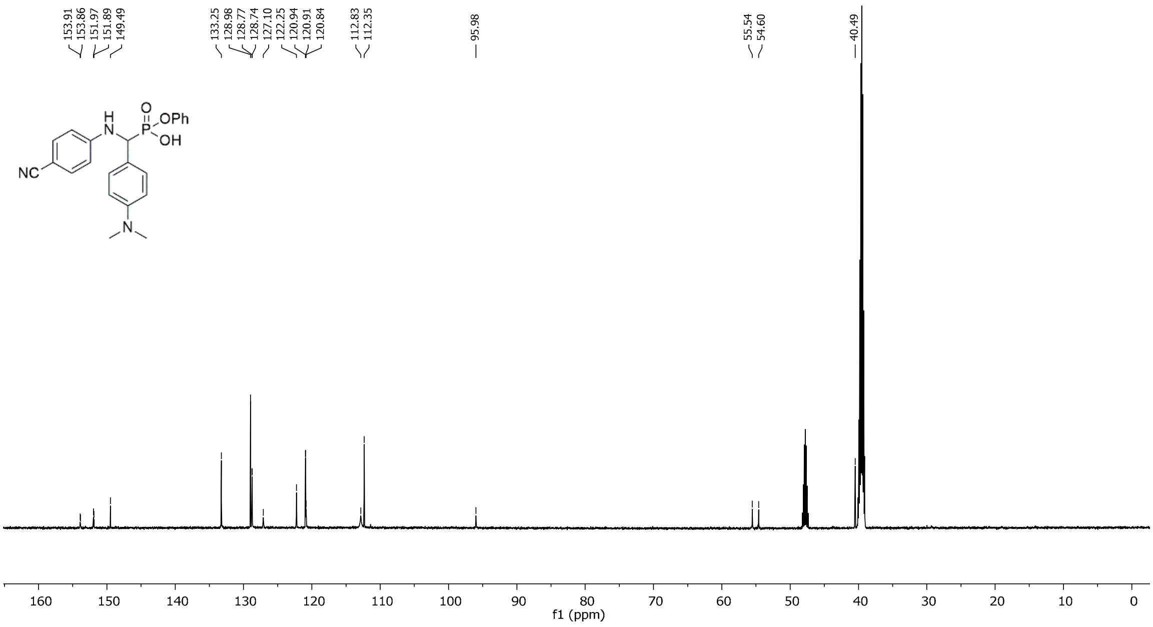

Figure S84. ^{13}C NMR (151 MHz, CD_3OD/DMSO-d_6) of phenyl hydrogen (((4-cyanophenyl)amino)(4-(dimethylamino)phenyl)methyl)phosphonate (**26**)

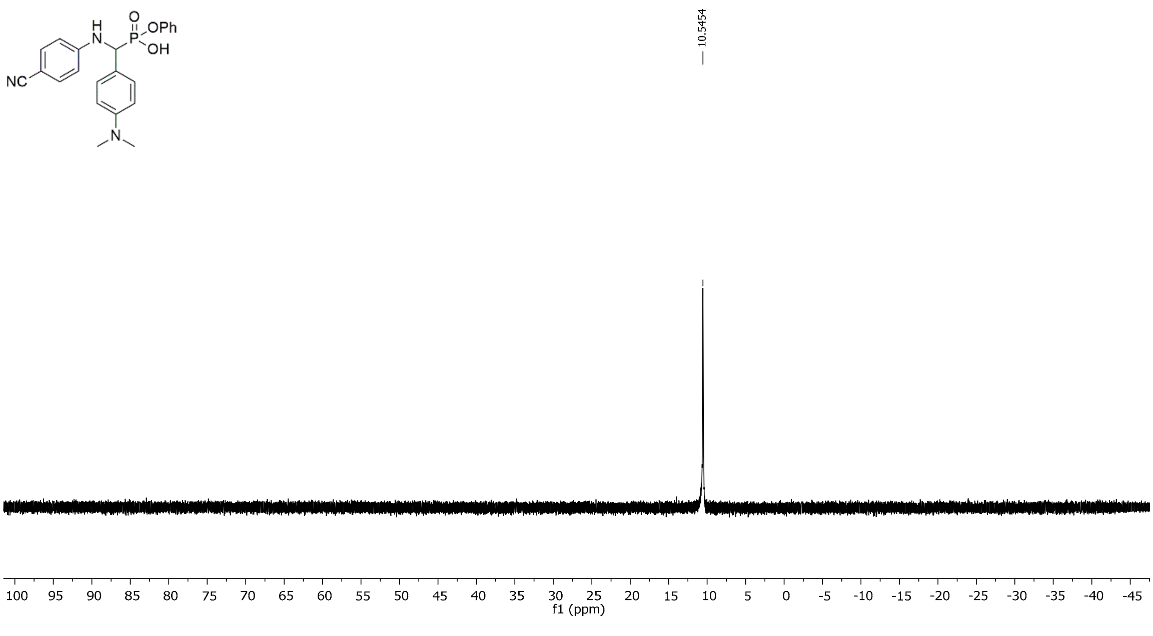

Figure S85. ^{31}P NMR (243 MHz, CD_3OD/DMSO-d_6) of phenyl hydrogen (((4-cyanophenyl)amino)(4-(dimethylamino)phenyl)methyl)phosphonate (**26**)

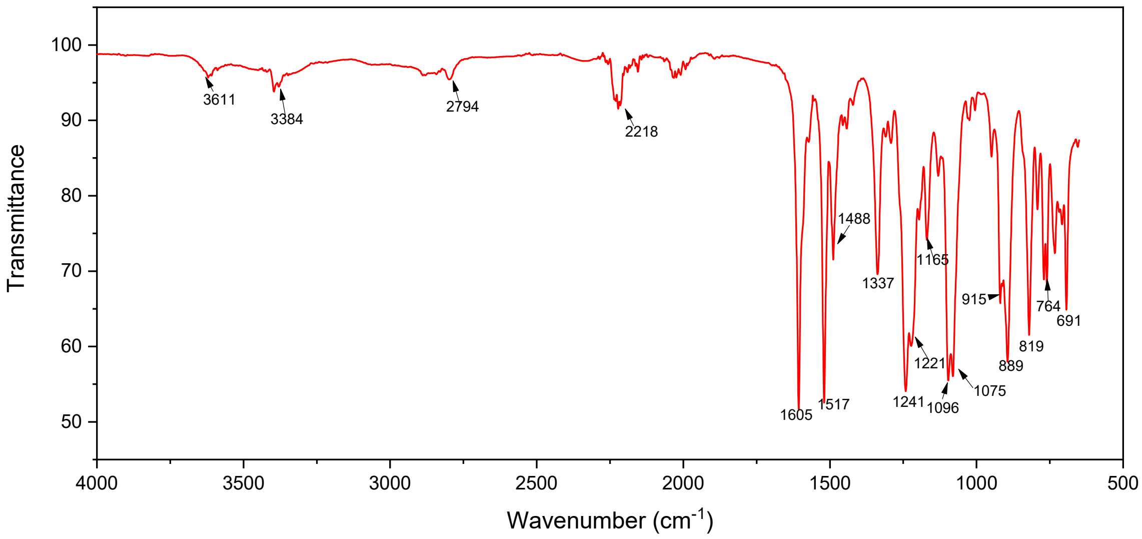

Figure S86. IR spectrum of phenyl hydrogen (((4-cyanophenyl)amino)(4-(dimethylamino)phenyl)methyl)phosphonate (**26**)

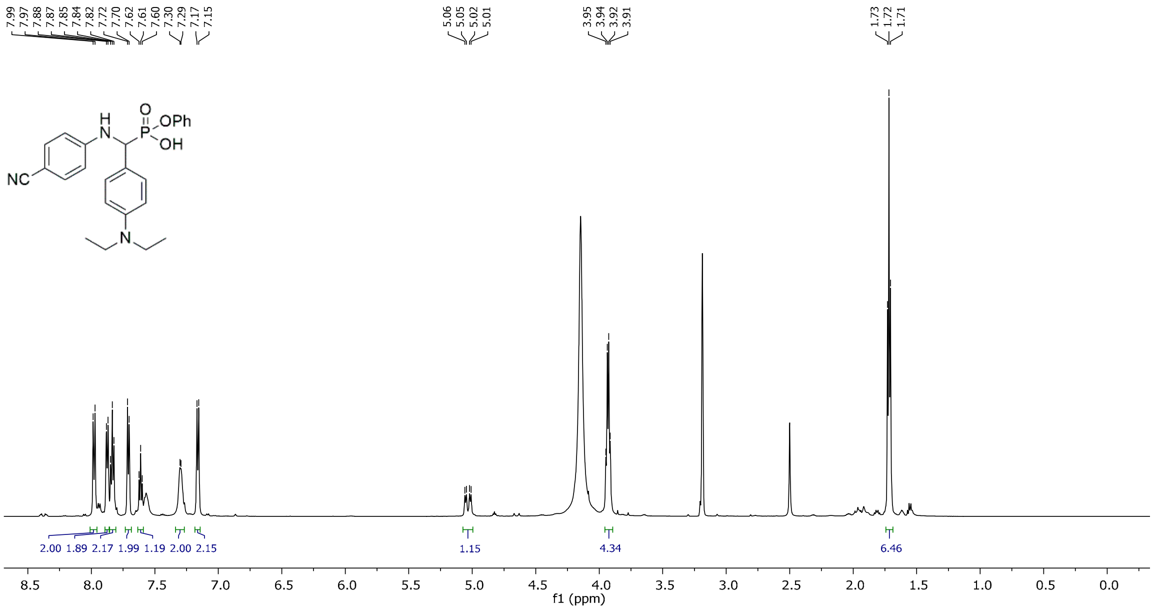

Figure S87. ^{1}H NMR (600 MHz, DMSO-d_6) of phenyl hydrogen (((4-cyanophenyl)amino)(4-(diethylamino)phenyl)methyl)phosphonate (**27**)

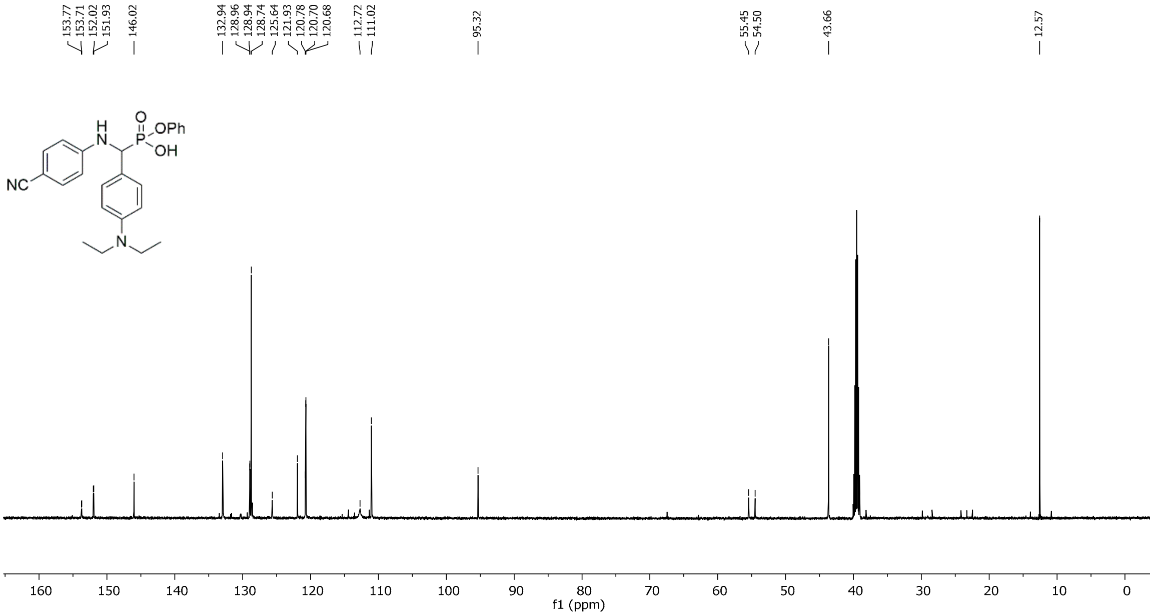

Figure S88. ^{13}C NMR (151 MHz, DMSO-d_6) of phenyl hydrogen (((4-cyanophenyl)amino)(4-(diethylamino)phenyl)methyl)phosphonate (**27**)

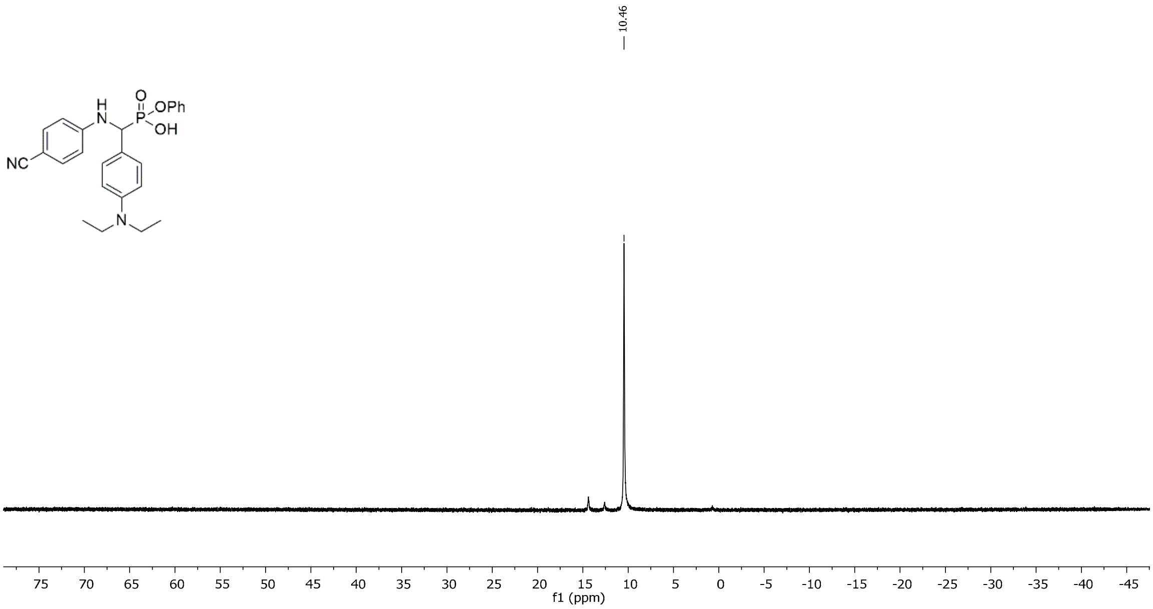

Figure S89. ^{31}P NMR (243 MHz, DMSO-d_6) of phenyl hydrogen (((4-cyanophenyl)amino)(4-(diethylamino)phenyl)methyl)phosphonate (**27**)

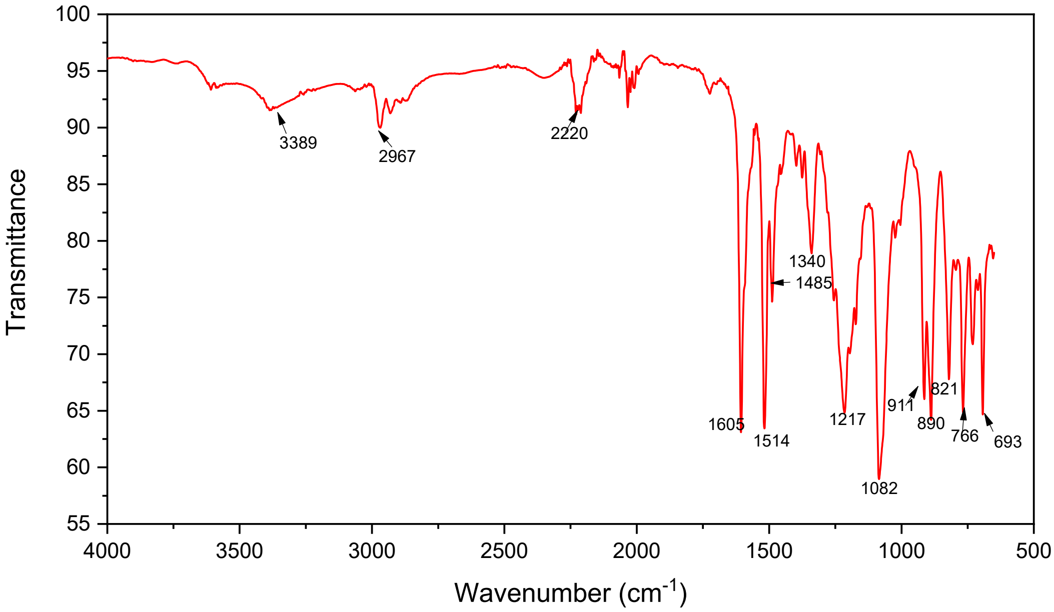

Figure S90. IR spectrum of phenyl hydrogen (((4-cyanophenyl)amino)(4-(diethylamino)phenyl)methyl)phosphonate (**27**)

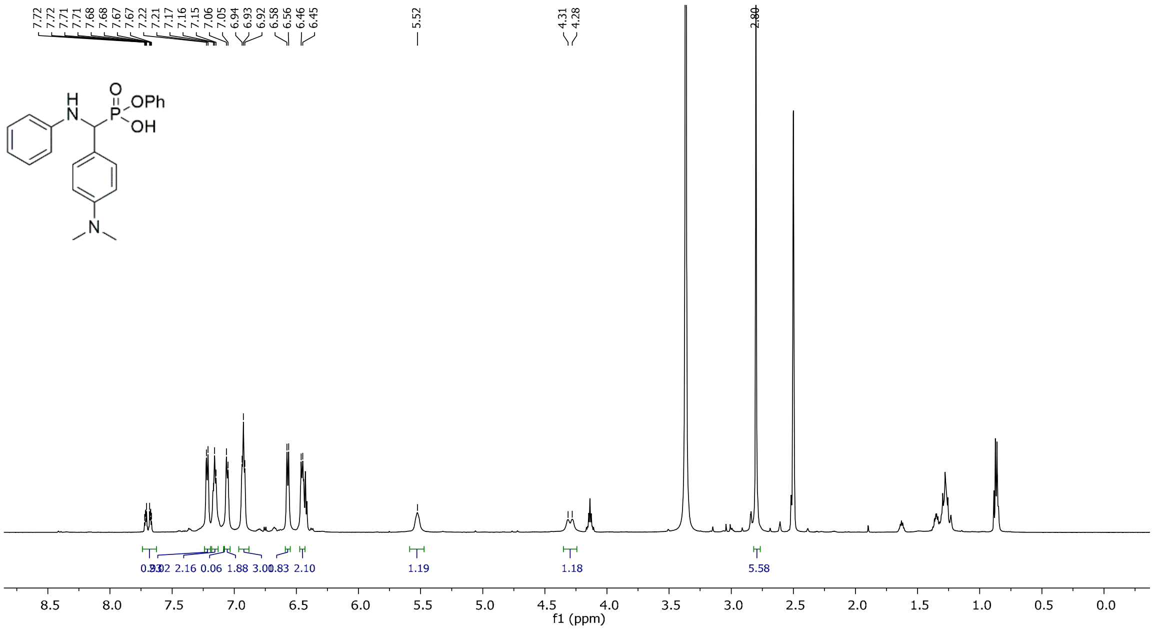

Figure S91. ^{1}H NMR (600 MHz, DMSO-d_6) of phenyl hydrogen ((4-(dimethylamino)phenyl)(phenylamino)methyl)phosphonate (**28**)

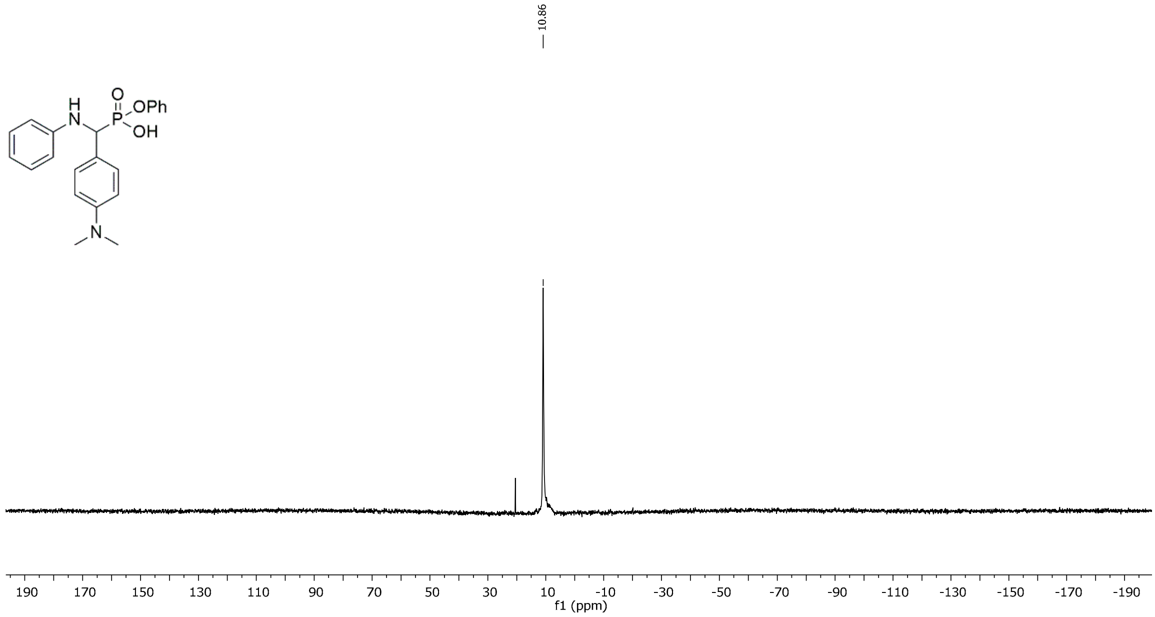

Figure S92. ^{31}P NMR (243 MHz, DMSO-d_6) of phenyl hydrogen ((4-(dimethylamino)phenyl)(phenylamino)methyl)phosphonate (**28**)

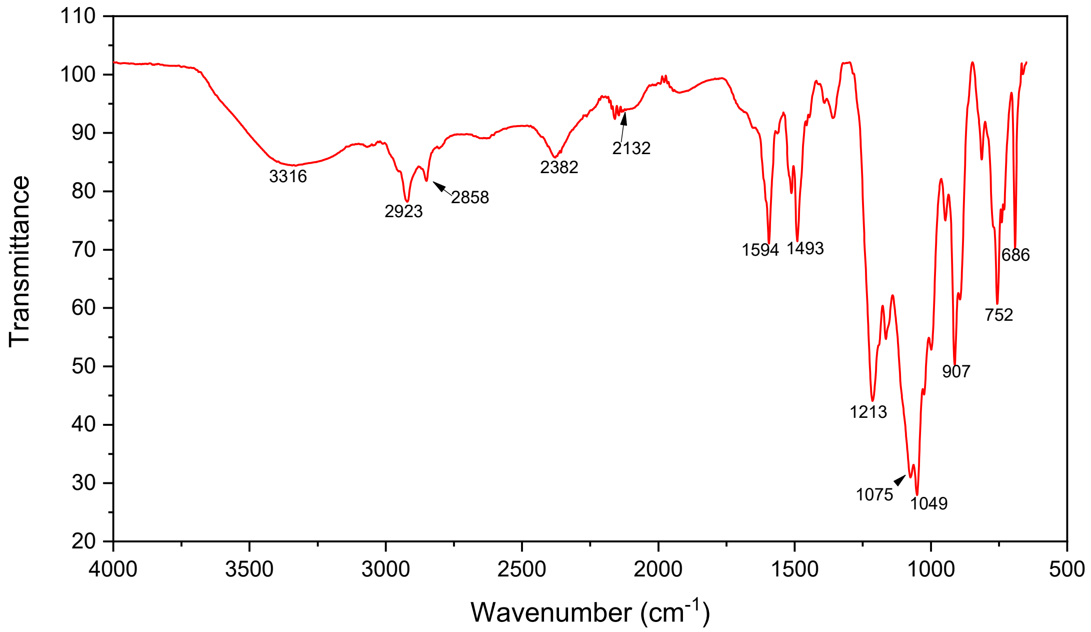

Figure S93. IR spectrum of phenyl hydrogen ((4-(dimethylamino)phenyl)(phenylamino)methyl)phosphonate (**28**)
